# Efficacy of Selenourea Organocatalysts in Asymmetric Michael Reactions under Standard and Solvent-Free Conditions

**DOI:** 10.3390/molecules26237303

**Published:** 2021-12-01

**Authors:** Mariola Zielińska-Błajet, Żaneta A. Mała, Rafał Kowalczyk

**Affiliations:** Faculty of Chemistry, Wrocław University of Science and Technology, Wybrzeże Wyspiańskiego 27, 50-370 Wrocław, Poland; zaneta.ignatiuk@pwr.edu.pl (Ż.A.M.); rafal.kowalczyk@pwr.edu.pl (R.K.)

**Keywords:** *Cinchona* alkaloids, selenoureas, hydrogen-bonding, Michael addition, ball-milling, organocatalysis, asymmetric synthesis

## Abstract

By varying the steric and electronic surroundings of the hydrogen-bonding motif, the novel chiral *Cinchona*-alkaloid based selenoureas were developed. Acting as bifunctional catalysts, they were applied in the Michael reactions of dithiomalonate and nitrostyrene providing chiral adducts with up to 96% ee. The asymmetric Michael–-hemiacetalization reaction of benzylidene pyruvate and dimedone, performed with the assistance of 5 mol% of selenoureas, furnished the product with up to 93% ee and excellent yields. The effectiveness of the new hydrogen-bond donors was also proved in solvent-free reactions under ball mill conditions, supporting the sustainability of the devised catalytic protocol.

## 1. Introduction

The asymmetric Michael addition reaction is widely recognized as one of the most important synthetic methods for the formation of C−C bonds [1]. In recent years, the organocatalytic version of this reaction deserves great attention. After the pioneering work of Jacobsen and Takemoto [2,3], significant progress has been made in the development and application of bifunctional chiral thiourea catalysts in catalytic asymmetric reactions. Subsequently, an inexpensive and readily available *Cinchona* alkaloid has been used as a chiral scaffold for the synthesis of thiourea catalysts and proved effective in several asymmetric reactions with excellent enantioselectivity [4,5]. 

Following the trend of the application of ureas that were successfully replaced by the thioureas, the natural consequence was the application of selenoureas. We were, thus, intrigued by how the change of hydrogen-bonding affinity of selenoureas in a chiral framework would impact the chirality transfer in comparison to well-developed thioureas. Nevertheless, to our surprise, the application of chiral selenoureas as hydrogen-bond donors in organocatalysis has remained rather scarcely reported. Only a single example of the application of a selenourea derivative as a chiral catalyst in the asymmetric Michael addition of α-nitrocyclohexane to aryl nitroalkenes was provided by Bolm et al. [6]. Recently, we have modified the *Cinchona* alkaloid scaffold by substituting the thiourea with a selenourea moiety, reporting the first example of the asymmetric Michael addition catalyzed by *Cinchona*-based selenoureas [7]. Our preliminary attempts at organocatalyzed Michael addition of nitromethane and thioacetic acid to trans-chalcone resulted in low-to-excellent yields and enantioselectivities under mild reaction conditions. For some transformations requiring long reaction times, the catalysts may have degraded, causing a further deterioration of the reaction outcome performed in solution (standard conditions). We hypothesized that the application of solvent-free conditions under ball-milling might substantially shorten the reaction time hampering, and thus decrease the catalyst decomposition.

In this context, we attempted the evaluation of the *Cinchona*-derived selenoureas activity as organocatalysts in the asymmetric Michael reactions under solvent-free conditions, performing reactions under ball-mill conditions. This mechanochemical technique has received significant research interest in synthetic chemistry [8,9,10,11,12], especially in asymmetric organocatalysis, including Michael reactions [13,14,15,16,17]. Nevertheless, little attention has been paid to H-bonding-mediated enantioselective Michael additions using bifunctional *Cinchona*-based organocatalysts [18,19], which is surprising, because the ball-milling proved to be a superior technique for applying the various thiourea-based organocatalysts [20].

Herewith, we would like to report the seminal report on asymmetric Michael additions of dithiomalonate to nitrostyrene and dimedone to β,γ-unsaturated α-keto ester catalyzed by a novel *Cinchona*-derived selenourea catalysts under standard and solvent-free conditions. The efficiency of the selenoureas will be discussed to compare their chirality-transfer abilities with those provided by thioureas. The question of their applicability in the hydrogen-bonding catalysis under solvent-free conditions using ball-milling will also be addressed.

## 2. Results and Discussion

### 2.1. Synthesis of Cinchona Selenoureas 

**Scheme 1:**
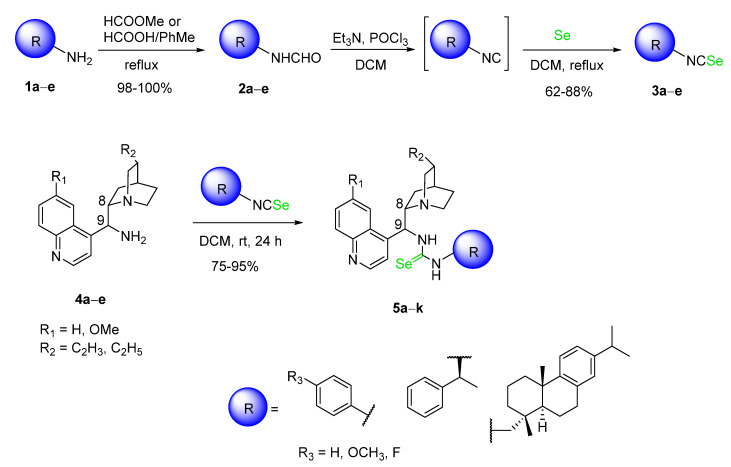
General synthesis of *Cinchona* alkaloid-derived selenoureas.

*Cinchona* alkaloid-based selenoureas **5a–k** were prepared from isoselenocyanates **3a–e** and 9-*epi*-aminoalkaloids **4a–e**, according to a procedure described in our previous publications [7]. The 9-Amino-(9-deoxy)-*epi*alkaloids **4a–e** were obtained in good yields (65–75%) from naturally occurring *Cinchona* alkaloids, following the literature procedure [21,22]. Alkyl and aryl isoselenocyanates **3a–e** were synthesized in a three-step protocol (Scheme 1) that involved the *N*-formylation of the commercially available amines with formic acid in toluene (amines **1a–c**) or in methyl formate (amines **1d** and **1e**), followed by the conversion of the formamides **2a–e** into isocyanides upon dehydration with POCl_3_ in the presence of NEt_3_ in dichloromethane [23]. Next, black selenium powder was added at once and the corresponding mixture was stirred in DCM at 45 °C in darkness, affording isoselenocyanates **3a–e** in good yields (62–88%). 

**Figure 1:**
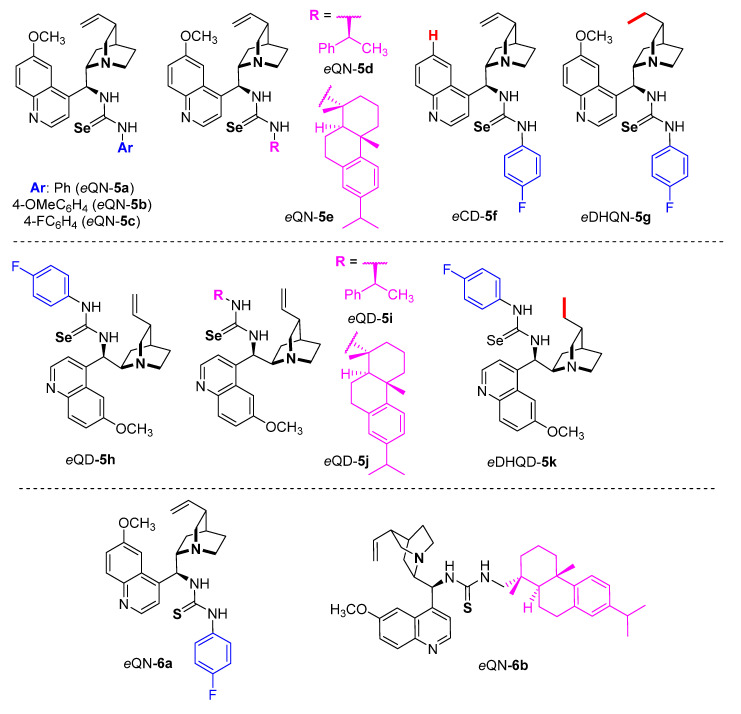
Structure of the synthesized *Cinchona*-based catalysts.

Finally, the desired selenoureas **5a–k** (Figure 1) were obtained in high yields (75–95%), furnishing the potential catalysts derivatizing the selenourea character (**5d** and **5e**), the alkaloid core (**5c** and **5g**) in a quinine-based series, along with analogous modifications of quinidine-based congeners (**5h–k**, Figure 1). In addition, the two thioureas (**6a** and **6b**) were synthesized to provide an alternative hydrogen-bonding unit to compare the effectiveness of the selenoureas versus the thioureas in the tested reactions.

### 2.2. Bifunctional Selenourea-Based Catalysts in Various Conjugate Additions 

In an attempt to evaluate the catalytic activity of the selenoureas **5a–5k**, Michael addition reactions with differing nucleophile structures (acyclic **8** and cyclic dimedone **11**) and the electrophilicity of the Michael acceptor were chosen as test reactions. Moreover, two different approaches for asymmetric reactions under a conventional organic solvents medium and solvent-free reactions driving the reaction progress by mechanical energy (mechanochemistry) were presented. 

**Scheme 2:**
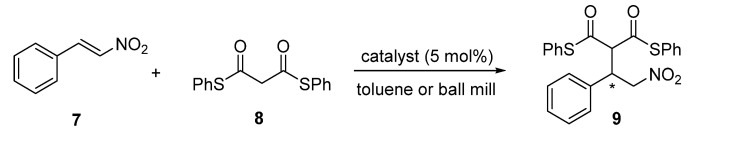
Michael addition of *S,S*′-diphenyl dithiomalonate **8** to trans-β-nitrostyrene **7**.

Initially, the asymmetric Michael addition between *S*,*S*′-diphenyl dithiomalonate **8** and trans-β-nitrostyrene **7** was examined in the presence of 5 mol% of the catalysts **5** and **6** in toluene at 273 K (Scheme 2). The selenourea catalysts **5a–k** afforded Michael adducts in nearly quantitative yields, except for catalyst **5k,** for which application led to the product with an 89% yield. However, a good-to-excellent enantiomeric excess, ranging from 85% to 96% ee, was noted for all the tested catalysts (Table 1, entries 1–11), indicating that the substitution pattern in selenoureas affected the enantioselectivity, albeit to a rather small extent (cf. **5c** and **5d**). 

Moreover, no significant differences were observed in the enantiomeric ratio of the product **9** when quinine or quinidine-based organocatalysts were applied, resulting in the adduct with an opposite configuration formation of 95% for **5c**, or 96% ee in the case of **5h**. Hence, (8*S*,9*S*)-quinine derived selenoureas gave the product **9** of *S* configuration, but for (8*R*,9*R*)-quinidine derivatives, the *R*-**9** was formed. Therefore, the configuration of the alkaloid at the C8/9 positions determined the stereochemical outcome of the addition. Although the small deviations of the performance of the catalysts were noted for the selenourea analogues, the nature of the hydrogen-bonding motif turned out to be crucial for achieving high stereoselectivities. Thereby, a comparison of different hydrogen-bond donor moieties in the catalysts revealed that the application of selenourea **5e** afforded products with higher stereoselectivity than those obtained in the reaction catalyzed by the analogous thiourea **6b** (Table 1, entries 5 vs. 13). On the contrary, both selenoureas **5c** and thiourea **6a** performed well, leading to product *S*-**9** with 95% ee for both catalysts (Table 1, entries 3 vs. 12). Further investigation proved the detrimental effect of temperature on the enantioselectivity for tested catalysts **5c–e** and **5h–j** in the Michael reaction at room temperature (Table 1, entry 3–5 and 8–10). 

The detrimental effect of the temperature to stereoselectivities in the studied reactions was not a crucial factor while performing under mechanochemical conditions. However, the local temperature rising during friction in the ball mill’s chamber suggested a decrease in enantioselectivities. Intrigued by the effects of the milling on reaction outcome, in the second part of our work, the asymmetric Michael addition under solvent-free conditions was explored. The reaction between *S,S*′-diphenyl dithiomalonate **8** and trans-β-nitrostyrene **7**, in the presence of 5 mol% of catalyst, was carried out in the planetary ball mill. The results are presented in Table 2.

Selenourea catalysts proved to be highly efficient catalysts in Michael additions between *S*,*S*′-diphenyl dithiomalonate **8** and trans-β-nitrostyrene **7**, and also under solvent-free conditions. Nearly complete conversions were achieved in a short time (30 min, Table 2). The Michael products **9** were formed in high yields and with moderate-to-good stereoselectivities, ranging from 69% to 93% ee (Table 2, entries 1–11). Nevertheless, among the tested selenourea catalysts, mechanochemical-approach reactions led to lower enantioselectivities, compared to the standard conditions (toluene, 273 K) in all tested transformations, except for the quinidine-derived catalysts **5h–k** and *e*DHQN-**5g** (Table 2, entries 7–11). A significant deterioration in selectivity was observed for quinine-derived thiourea **6a** and **6b**, compared with results obtained in solvent (76 and 95% ee vs. 59 and 77% ee, Table 1 and Table 2, entries 12, 13). Moreover, similar results to reactions with toluene at 298 K were attained by solvent-less mechanochemical ball-milling (Table 1 and Table 2, entries 3–5 and 8–10). Hence, the ball-milling offers an alternative approach to the chiral products, leading to similar results as the standard transformations under solvent conditions. Moreover, the different chiral catalysts’ structures provided the best enantioselectivities, depending on the technique applied, as proved for **5g** and **5i**, for which application offered the best results under ball mill conditions, in contrast to reactions in toluene at both temperatures.

**Scheme 3:**
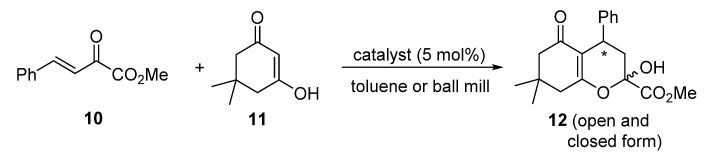
Asymmetric Michael–hemiacetalization reaction of benzylidene pyruvate **10** and dimedone **11**.

Exploring the effectiveness of the selenoureas-based catalysts, we wondered about the reaction outcome when different nucleophiles and electrophiles are applied. Therefore, we were intrigued by the usage of dimedone that acts as a one-binding point nucleophile (through a C-OH/H hydrogen bond), but the two-center electrophile was **10**. Thereby, Michael addition/hemiacetalization of diketones to the benzylidene pyruvate esters, leading to bicyclic compounds with two stereogenic centers, were studied. Particularly, the reaction of dimedone **11** with benzylidene pyruvate **10** has been successfully carried out with the use of various types of catalysts [25,26,27,28,29,30,31,32]. An asymmetric Michael–hemiacetalization cascade reaction (Scheme 3) was used for the screening of chiral selenoureas’ performance under standard and solvent-less conditions. The results of our studies are summarized in Table 3 and Table 4.

All *Cinchona* alkaloid-derived selenourea catalysts **5a–k** efficiently catalyzed the reaction, affording the product **12** with excellent yields (up to 97%) and with good enantioselectivities, ranging from 64–94% (Table 3). Quinidine-derived catalysts bearing electron-withdrawing 4-fluorophenyl groups **5h** and **5k** on the selenourea moiety outperformed the quinine derivatives **5c** and **5g** (73 and 82% ee vs. 65 and 69% ee, Table 3, entries 3, 7, 8, and 11). Both quinine and quinidine series with alkyl groups in the selenourea moiety overcame the aryl-substituted selenoureas in terms of chirality transfer effectiveness. The most effective catalysts, **5e** and **5j**, with a large dehydroabietyl group, afforded the best enantioselectivities (94 and 93% ee, entries 5 and 10, Table 3). However, opposite results were obtained for quinine-derived thiourea **6a** and **6b**. Hence, catalyst **6a** with 4-fluorophenyl group turned out to be more effective than its selenium counterpart **5c** (91% ee vs. 65%, entry 3 and 12, Table 3). Moreover, the stereochemical outcome of the reaction was only determined by the absolute configuration of used catalysts. It might be pointed out the reactions performed at 273 K led to lower enantioselectivities than the respective transformations at 298 K. The results might be rationalized by the assumption that the dimedone in an enol form could create an alternative hydrogen-bonding unit to the catalyst’s surroundings or by the aggregation effects.

An alternative approach investigated the mechanochemical effects induced by ball-milling in the above selenourea-catalyzed asymmetric Michael–hemiacetalization reaction. Considering the aggregation effects, or the dimedone-dimeric forms presented in the reaction, we were intrigued by the reaction outcome when the solvent-less conditions were applied. However, the application of catalysts **5a–5k**, and also thioureas **6a,b**, led to lower conversions (32–98%, Table 4) along with a significant enantioselectivities lowering (34–73% ee, Table 4), compared to the results achieved in the solution-based protocol.

In general, the selectivity trend was analogous to the one observed in solution, and the best result was obtained for quinidine-based catalyst **5j** with dehydroabietyl group in the selenourea moiety (73% ee, Table 4).

## 3. Materials and Methods

### 3.1. General Information

The solvents and reagents were received from commercial suppliers and used without additional purification. Reactions were monitored by thin-layer chromatography (TLC) on silica gel 60 F-254 precoated plates (Merck, Darmstadt, Germany), and spots were visualized with a UV lamp. Products were purified by standard column chromatography on silica gel 60 (230–400 mesh) (Merck, Darmstadt, Germany). ^1^H and ^13^C NMR spectra (400 and 125 MHz or 600 and 151 MHz) were recorded in CDCl_3_ on a JEOL ECZ400S instrument (Jeol Ltd., Tokyo, Japan) and Bruker Avance II 600 instrument (Bruker, Billerica, MA, USA), respectively. High-resolution mass spectra (HRMS) were measured on a Waters LCT Premier XE TOF instrument (Waters Corporation, Milford, MA, USA) with electrospray ionization. Optical rotations were measured using an Optical Activity Ltd. Model AA-5 automatic polarimeter (Optical Activity Ltd., Huntington, UK). Melting points were determined using a Boëtius hot-stage apparatus (PHMK VEB Analytic, Dresden, Germany). HPLC analysis was performed on SHIMADZU NEXERA X2 apparatus (Chiral Technologies INC. West Chester, PA, USA) using CHIRALPAK IA-3 and IB-3 chiral columns (4.6 mm × 25 cm) without a guard column. Each HPLC analysis was controlled by comparison with the pure sample and the racemate. Ball-milling was realized in a planetary ball mill PM-200 (Retsch GmbH, Haan, Germany) in stainless steel 12-mL containers with stainless steel grinding balls with a diameter of 10 mm.

### 3.2. Preparation of Starting Compounds 

The 9-Amino-(9-deoxy)-*epi*alkaloids **4a–e** were synthesized from commercially available *Cinchona* alkaloids (QN, CD, QD, DHQN, and DHQD) (Buchler GmbH) according to a procedure described in the literature [7,21,22,33]. 

#### 3.2.1. Preparation of Formamides **2a–e**

Formamide **2a–c** were obtained by *N*-formylation of amines **1a–c** with formic acid in toluene, according to reported procedures [23]. Amine **1d–e** (30.0 mmol, 1 eq) was placed in a Teflon-capped ampoule and dissolved in 40 mL of methyl formate (600.0 mmol, 20 eq). The mixture was heated to 45 °C for 2–3 days. The solvent was removed in vacuo, yielding compound **2d–e** as solidifying oil quantitatively.

*N-Phenylformamide***2a**. Yield 98%, beige solid. The mixture of two rotamers (ratio 1.1:1) was observed in the NMR spectra. ^1^H NMR (400 MHz, CDCl_3_): δ 7.05–7.16 (m, 2H), 7.17–7.25 (m, 2H), 7.29–7.37 (m, 5H), 7.55 (d, *J* = 8.0 Hz, 2H), 8.18 (s, 1H), 8.34 (br s, 1H), and 8.69 (d, *J* = 11.3 Hz, 1H) ppm. The spectral data are in agreement with the reported values [34].
*N-(4-Methoxyphenyl)formamide***2b**. Yield 99%, yellow solid. The mixture of two rotamers (ratio 1:1) was observed in the NMR spectra. ^1^H NMR (400 MHz, CDCl_3_): δ 3.79 (s, 3H), 3.80 (s, 3H), 6.90–6.84 (m, 4H), 7.03 (d, *J* = 8.9 Hz, 2 H), 7.32 (br s, 1H) 7.44 (d, *J* = 8.9 Hz, 2H), 7.97 (br s, 1H), 8.32 (s, 1H), and 8.50 (d, *J* = 11.4 Hz) ppm. The spectral data are in agreement with the reported values [34].
*N-(4-Fluorophenyl)formamide***2c**. Yield 100%, yellow solid. The mixture of two rotamers (ratio 1.7:1) was observed in the NMR spectra. ^1^H NMR (400 MHz, CDCl_3_): δ, 6.99–7.18 (m, 5H), 7.34–7.66 (m, 5 H), 8.34 (m, 1H), and 8.56 (d, *J* = 12.6 Hz, 1H) ppm. The spectral data are in agreement with the reported values [35].
*N-[(1S)-1-Phenylethyl]formamide***2d**. Yield 99%, brown oil. The mixture of two rotamers (ratio 5:1) was observed in the NMR spectra. ^1^H NMR (600 MHz, CDCl_3_): δ 1.52 (d, *J* = 6.9 Hz, 3H), 1.56 (d, *J* = 6.9 Hz, 3H), 4.69 (quint, *J* = 6.9 Hz, 1H), 5.21 (quint, *J* = 7.0 Hz, 1H), 5.95 (br s, 1H), 6.09 (br s, 1H), 7.24–7.38 (m, 10 H), and 8.15 (m, 2H) ppm. The spectral data are in agreement with the reported values [36].
*N-{[(1R,4aS,10aR)-1,4a-Dimethyl-7-isopropyl-1,2,3,4,4a,9,10,10a-octahydrophenanthren-1-yl] methyl}formamide***2e**. Yield 100%, brown oil. The mixture of two rotamers (ratio 2.8:1) was observed in the NMR spectra. ^1^H NMR (600 MHz, CDCl_3_): major rotamer, δ 0.95 (s, 3H), 1.22–1.29 (m, 10H), 1.35–1.44 (m, 3H), 1.66–1.78 (m, 3H), 1.87–1.90 (m, 1H), 2.30 (d, *J* = 12.8 Hz, 1H), 2.80–2.87 (m, 2H), 2.90–2.94 (m, 1H), 3.13 (dd, *J* = 6.6, 13.8 Hz, 1H), 3.29 (dd, *J* = 13.7, 6.7 Hz, 1H), 5.55 ( br s, 1H), 6.89 (dd, *J* = 1.0, 7.2 Hz, 1H), 6.99 (dd, *J* = 1.6, 8.2 Hz, 1H), 7.16 (dd, *J* = 2.5, 8.2 Hz, 1H), and 8.20 (s, 1H) ppm. HRMS (ESI): *m*/*z* calculated for [C_21_H_31_NO + Na]^+^: 336.2303, found: 336.2324. The spectral data are in agreement with the reported values [37].

#### 3.2.2. Preparation of Isoselenocyanates **3a–e**

According to the procedure described in [38,39]: to a cooled to −10 °C mixture of the formamide **2a–e** (0.100 mol, 1.00 equiv.), Et_3_N (33.4 g, 46.0 mL, 0.330 mol, 3.30 equiv.) in CH_2_Cl_2_ (100 mL) was added dropwise POCl_3_ (16.9 g, 10.3 mL, 0.110 mmol, 1.10 equiv.) for over 15 min. The resulting mixture was stirred at −10 °C for 1 h, and additionally, at room temperature for 1 h (until the consumption of formamide, monitored by TLC). The reaction mixture was neutralized using a saturated solution of NaHCO_3_ (200 mL) and extracted with CH_2_Cl_2_. Combined organic phases were dried over anhydrous Na_2_SO_4_ and concentrated under a vacuum. The obtained crude isocyanides (10.0 mmol, 1.00 equiv.) were placed in a Teflon-capped ampoule and dissolved in CH_2_Cl_2_ (3.00 mL). Next, selenium (1.579 g, 20.0 mmol, 2.00 equiv.) was added to the ampoule. The reaction mixture was heated to 45 °C for 24 h and then it was filtered through a Celite pad and the solvent was removed in vacuo. The crude product was purified by column chromatography (SiO_2_, CH_2_Cl_2_/petroleum ether), affording isoselenocyanate **3a–e**.

*N-Phenylisoselenocyanate***3a**. Yield 62%, beige oil. ^1^H NMR (600 MHz, CDCl_3_): δ 3.81 (s, 3H), 6.86 (d, *J* = 8.2 Hz, 2H), and 7.23 (d, *J* = 8.3 Hz, 2H) ppm. The spectral data are in agreement with the reported values [38].
*N-(4-Methoxyphenyl)isoselenocyanate***3b**. Yield 68%, light yellow solid. ^1^H NMR (600 MHz, CDCl_3_): δ 3.81 (s, 3H), 6.86 (d, *J* = 8.2 Hz, 2H), and 7.23 (d, *J* = 8.3 Hz, 2H) ppm. The spectral data are in agreement with the reported values [38].
*N-(4-Fluorophenyl)isoselenocyanate***3c**. Yield 69%, solidifying yellow oil. ^1^H NMR (600 MHz, CDCl_3_): δ 6.93–7.01 (m, 2H), and 7.15–7.23 (m, 2H) ppm. The spectral data are in agreement with the reported values [38].
*[(1S)-1-Isoselenocyanatoethyl]benzene***3d**. Yield 82%, dark yellow oil. ^1^H NMR (600 MHz, CDCl_3_): δ = 1.71 (d, *J* = 6.8 Hz, 3H), 4.99 (q, *J* = 6.8 Hz, 1H), 7.31–7.36 (m, 3H), and 7.37–7.42 (m, 2H) ppm. ^13^C NMR (150 MHz, CDCl_3_): δ 24.7, 57.7, 125.5 (2C overlapped), 128.6, 129.0, 129.1 (2C overlapped), and 139.1 ppm. HRMS (ESI): m/z calculated for [C_9_H_9_NSe + H]^+^: 211.9973, found: 211.9969.
*(1R,4aS,10aR)-1,4a-Dimethyl-7-isopropyl-1-(isoselenocyanatomethyl)-1,2,3,4,4a,9,10,10a-octahydrophenanthrene***3e**. Yield 88%, yellow oil. ^1^H NMR (600 MHz, CDCl_3_): δ 0.99 (s, 3H), 1.22–1.23 (m, 9H), 1.44–1.46 (m, 3H), 1.56 (td, *J* = 10.3, 2.2Hz, 2H), 1.65–1.68 (m, 1H), 1.72–1.80 (m, 2H), 2.31 (m, 1H), 2.83 (hept. *J* = 6.9 Hz, 1H), 2.89–2.92 (m, 1H), 3.09–3.10 (m, 1H), 3.40 (d, *J* = 14.4 Hz, 1H), 3.51 (d, *J* = 14.4 Hz, 1H), 6.89 (dd, *J* = 1.1 Hz, 1H), 7.01 (dd, *J* = 8.1, 1.8 Hz, 1H), and 7.16 (d, *J* = 8.2 Hz, 1H) ppm. ^13^C NMR (150 MHz, CDCl_3_): δ 18.1, 18.6, 19.2, 24.1, 25.3, 30.2, 33.5, 36.5, 37.7, 38.2, 38.5, 45.9, 57.3, 124.2, 124.4, 126.9, 134.3, 145.9, and 146.3 ppm. HRMS (ESI): *m*/*z* calculated for [C_21_H_29_NSe + H]^+^: 376.1544, found: 376.1541.

### 3.3. General Procedure for the Synthesis of Selenourea Catalysts ***5a–k***

The syntheses of the catalysts **5a–c, 5f–h**, and **5k** were previously reported [7]. Compounds **5d**, **5e**, **5i**, and **5j** are novel. 9-Amino-(9-deoxy)-*epi*alkaloid **4** (1.00 mmol, 1.00 equiv.) was dissolved in CH_2_Cl_2_ (5.0 mL), and a solution of appropriate aryl isoselenocyanate **3** (1.00 mmol, 1.00 equiv.) in CH_2_Cl_2_ (1.0 mL) was added. The mixture was stirred for 15 h at room temperature under argon and in darkness. The solvent was evaporated in vacuo and the residue was purified by chromatography on silica gel (CH_2_Cl_2_/MeOH, 10:1) to afford the desired product **5**. The structure of catalysts (**5d**, **5e**, **5i**, and **5j**) were confirmed by spectroscopic methods (^1^H, and ^13^C NMR) and high-resolution mass spectrometry (data available in Supplementary Materials).

*N-[(8S,9S)-6′-Methoxycinchonan-9-yl]-N′-[(S)-1-phenylethyl]selenourea eQN-* **5d**
Yield 94%, pale yellow solid, mp 130−132 °C, *R*_f_ = 0.35 (CH_2_Cl_2_/MeOH 10:1). ${\left[\alpha \right]}_{D}^{23}\;$ = −136.6 (c 0.22, CH_2_Cl_2_). ^1^H NMR (400 MHz, CDCl_3_): δ 0.91 (s, 1H), 1.25–1.61 (m, 8H), 2.12–3.06 (m, 6H), 3.94 (s, 3H), 4.88–4.93 (m, 2H), 5.33 (br s, 1H), 5.55–5.64 (m, 1H), 7.29–7.40 (m, 9H), 7.56 (br s, 1H), 8.04 (d, *J* = 6.7 Hz, 1H), and 8.74 (d, *J* = 4.5 Hz, 1H) ppm. ^13^C NMR (101 MHz, CDCl_3_): δ 25.6, 27.3, 27.4, 27.6, 29.8, 39.3, 40.4, 50.8, 55.0, 55.8, 61.2, 102.3, 115.0, 120.0, 122.0, 126.1, 127.9 (2C overlapped), 129.1 (2C overlapped), 132.0, 140.9, 142.0 (2C overlapped), 144.8, 147.7 (2C overlapped), 158.1, and 179.8 ppm. HRMS (ESI): *m*/*z* calculated for C_29_H_35_N_4_O^80^Se [M + H]^+^: 535.1971, found: 535.1985.
*N-{[(1R,4aS,10aR)-1,4a-Dimethyl-7-izopropyl-1,2,3,4,4a,9,10,10a-octahydrophenanthrene-1-yl]methyl}-N′-[(8S,9S)-6′-methoxycinchonan-9-yl]selenourea eQN-* **5e**
Yield 92%, pale yellow solid, mp 137−139 °C, *R*_f_ = 0.52 (CH_2_Cl_2_/MeOH 10:1). ${\left[\alpha \right]}_{D}^{23}\;$ = −66.3 (c 0.23, CH_2_Cl_2_). ^1^H NMR (600 MHz, CDCl_3_): δ 0.68–0.79 (m, 4H), 0.83–1.18 (m, 6H), 1.20–12.00 (m, 16H), 1.96–2.24 (s, 1H), 2.35 (s, 1H), 2.53 (s, 1H), 2.66–3.43 (m, 8H), 3.95–4.00 (m, 4H), 4.83–5.08 (m, 2H), 5.62–5.67 (m, 1H), 6.40 (br s, 1H), 6.83 (s, 1H), 6.93–7.17 (m, 2H), 7.24–7.69 (m, 3H), 7.74–8.20 (m, 2H), and 8.50 (br s, 1H) ppm. ^13^C NMR (151 MHz, CDCl_3_): δ 18.4 (2C overlapped), 18.7, 19.1 (2C overlapped), 24.0, 24.1 (2C overlapped), 25.4, 27.2, 29.7, 30.0 (2C overlapped), 33.5 (3C overlapped), 36.2, 37.2, 37.5, 37.6, 55.2, 55.3, 59.2, 100.8, 115.6, 122.3, 123.7 (2C overlapped), 124.1 (2C overlapped), 126.9, 127.1, 132.2, 134.6, 140.1, 145.0, 145.4, 146.8, 147.7, 158.4, and 180.5 ppm. HRMS (ESI): *m*/*z* calculated for C_41_H_55_N_4_O^80^Se [M + H]^+^: 699.3541, found: 699.3556.
*N-[(8R,9R)-6′-Methoxycinchonan-9-yl]-N′-[(S)-1-phenylethyl]selenourea eQD-* **5i**
Yield 86%, pale yellow solid, mp 123−125 °C, *R*_f_ = 0.33 (CH_2_Cl_2_/MeOH 10:1). ${\left[\alpha \right]}_{D}^{23}\;$ = +302.5 (c 0.24, CH_2_Cl_2_). ^1^H NMR (600 MHz, CDCl_3_): δ 0.88 (s, 1H), 1.12–1.65 (m, 8H), 2.32 (d, *J* = 8.2 Hz, 1H), 2.58–3.24 (m, 5H), 3.95 (s, 3H), 4.72–4.80 (m, 1H), 5.13 (m, 3H), 5.72–5.84 (m, 1H), 6.68–7.76 (m, 10H), 7.96 (s, 1H), and 8.15–8.84 (m, 1H) ppm. ^13^C NMR (151 MHz, CDCl_3_): δ 24.8, 26.0, 27.2, 38.6, 47.0, 48.7, 50.7, 53.4, 55.7 (2C overlapped), 57.7, 61.8, 102.1, 115.3, 118.8, 122.4, 126.0 (3C overlapped), 128.3, 128.8, 131.9, 139.4, 141.8, 144.6, 147.6 (2C overlapped), 158.2, and 178.7 ppm. HRMS (ESI): *m*/*z* calculated for C_29_H_35_N_4_O^80^Se [M + H]^+^: 535.1971, found: 535,1984.
*N-{[(1R,4aS,10aR)-1,4a-Dimethyl-7-izopropyl-1,2,3,4,4a,9,10,10a-octahydrophenanthrene-1-yl]methyl}-N′-[(8R,9R)-6′-methoxycinchonan-9-yl]selenourea eQD-* **5j**
Yield 89%, pale yellow solid, mp 144−146 °C, *R*_f_ = 0.40 (CH_2_Cl_2_/MeOH 10:1). ${\left[\alpha \right]}_{D}^{23}\;$ = +204.4 (c 0.23, CH_2_Cl_2_). ^1^H NMR (600 MHz, CDCl_3_): δ 0.48–0.66 (m, 4H), 0.71–1.16 (m, 8H), 1.16–1.68 (m, 13H), 1.68–1.92 (m, 2H), 1,98–2.23 (m, 1H), 2.35 (s, 1H), 2.46–3.43 (m, 9H), 3.57–4.14 (m, 3H), 5.00–5.24 (m, 2H), 5.76–6.00 (m, 1H), 6.37 (br s, 1H), 6.88 (s, 1H), 6.97 (d, *J* = 8.1 Hz, 1H), 7.08 (d, *J* = 8.0 Hz, 1H), 7.24–7.79 (m, 3H), 7.89–8.29 (m, 2H), and 8.58–9.00 (br s, 1H) ppm. ^13^C NMR (151 MHz, CDCl_3_): δ 18.0, 18.2, 19.0 (2C overlapped), 24.0 (3C overlapped), 25.2, 26.2, 27.2, 30.2, 33.5 (2C overlapped), 36.0, 37.0, 37.3, 37.9, 46.2, 46.8, 49.1 (2C overlapped), 55.7, 60.0, 99.4, 115.1, 122.9, 123.9 (2C overlapped), 124.2 (2C overlapped), 126.8 (2C overlapped), 132.3, 134.4, 139.9, 145.1, 145.6, 146.8, 147.8, 158.6, and 180.2 ppm. HRMS (ESI): *m*/*z* calculated for C_41_H_55_N_4_O^80^Se [M + H]^+^: 699.3541, found: 699.3564.

### 3.4. Preparation of Thiourea Catalysts ***6a*** and ***6b***

Synthesis of thiourea **6a** was performed as described in our previous publication [7]. Preparation of derivative **6b** was prepared according to a known procedures [40,41]. 

### 3.5. General Procedure for Michael Addition of S,S′-Diphenyl Dithiomalonate ***8*** to trans-β-Nitrostyrene ***7*** in a Solution 

A mixture of trans-β-nitrostyrene **7** (0.1 mmol), a catalyst **5** (5 mol%), and *S,S*′-diphenyl dithiomalonate **8** (0.1 mmol) in 0.5 ml of toluene was stirred for 5–90 min at 273 K (ice-water bath) or at 298 K. After the reaction was completed (monitored by TLC), the reaction mixture was filtered through a silica gel to remove the catalyst and concentrated in vacuo. The crude product **9** was analyzed by ^1^H NMR and HPLC. Some products (high ees) were purified by column chromatography on silica gel.

*(S)-2-(2-Nitro-1-phenylethyl)-1,3-bis(phenylsulfanyl)propane-1,3-dione***9**. The following product was obtained as an off-white solid, 108 mg, 99%, ${\left[\alpha \right]}_{D}^{23\;}$ = +123.0 (c 1.0, DCM), 95% ee. Mp 160.0–161.5 °C. ^1^H NMR (CDCl_3_, 400 MHz): δ 4.42–4.37 (m, 1H), 4.49 (d, *J* = 9.8 Hz, 1H), 4.89–4.80 (m, 2H), 7.16–7.13 (m, 2H), 7.28–7.26 (m, 2H), 7.39–7.31 (m, 6H), and 7.47–7.40 (m, 5H). ^13^C NMR (CDCl_3_, 125 MHz): δ 44.5, 69.4, 77.2, 126.2, 128.5, 128.7, 129.2, 129.5, 129.7, 130.2, 130.4, 134.3, 134.4, 135.3, 189.7, 190.5 ppm. HRMS (ESI) *m*/*z* calcd for C_23_H_19_O_4_S_2_ [M − H]^+^ 436.0677, found 436.0661. HPLC (Chiralcel IB-3, n-hexane/2-propanol, 9:1, flow rate 1 mL/min, λ = 205 nm): *t*_R_ = 17.1 min (major), *t*_R_ = 20.1 min (minor). The spectral data are in agreement with those reported in the literature [24].

### 3.6. General Procedure for Michael Addition of S,S′-Diphenyl Dithiomalonate ***8*** to trans-β-Nitrostyrene ***7*** Using Planetary Ball-Mill 

Trans-β-nitrostyrene **7** (0.25 mmol), the catalyst **5** (0.0125 mmol) and *S*, *S*′-diphenyl dithiomalonate **8** (0.25 mmol) were placed in a 12-mL stainless steel container and were milled in a planetary ball mill with two stainless steel grinding balls with a diameter of 10 mm for 30 min at 400 rpm. The reaction mixture was then dissolved in ethyl acetate and filtered through a plug of silica gel to remove the catalyst and concentrated in vacuo. The crude product **9** was analyzed by ^1^H NMR and HPLC. Some products (high ees) were purified by column chromatography on silica gel. 

### 3.7. General Procedure for the Asymmetric Michael-Hemiacetalization Reaction of Benzylidene Pyruvate ***10*** and Dimedone ***11*** in a Solution 

A mixture of benzylidene pyruvate **10** (0.1 mmol), a catalyst **5** (5 mol%), and dimedone **11** (0.1 mmol) in 0.5 mL of toluene was stirred for 30 min at 298 K. The reaction mixture was filtered through a silica gel to remove the catalyst and concentrated in vacuo. The crude product **12** was analyzed by ^1^H NMR and HPLC. Some products (high ees) were purified by column chromatography on silica gel.

*(4R)-4-Methyl-2-hydroxy-7,7-dimethyl-5-oxo-4-phenyl-3,4,5,6,7,8-hexahydro-2H-chromene-2-carboxylate***12**. The following **12** product was obtained as a colorless oil, 33 mg, 99%, 94%ee. ^1^H NMR (CDCl_3_, 400 MHz): δ 1.10 (d, *J* = 8.9 Hz, 3H), 1.19 (d, *J* = 9.5 Hz, 3H), 2.24–2.21 (m, 2H), 2.58–2.26 (m, 4H), 3.73 (d, *J* = 2.1 Hz, 2H), 3.84 (s, 1H), 3.91–3.86 (m, 1H), 4.65 (bs, 1H), 7.18–7.13 (m, 3H), 7.28–7.23 (m, 2H) ppm. HPLC (Chiralcel IA-3, n-hexane/2-propanol, 7:3, flow rate 1 mL/min, λ = 254 nm): *t*_R_ = 4.6 min (major), *t*_R_ = 6.0 min (minor). The spectral data are in agreement with those reported in the literature [25].

### 3.8. General Procedure for the Asymmetric Michael-Hemiacetalization Reaction of Benzylidene Pyruvate ***10*** and Dimedone ***11*** Using Planetary Ball-Mill 

Benzylidene pyruvate **10** (0.25 mmol), the catalyst **5** (5 mol%), and dimedone **11** (0.25 mmol) were placed in a 12-mL stainless steel container and were milled in a planetary ball mill with two stainless steel grinding balls with a diameter of 10 mm for 30 min at 400 rpm. The reaction mixture was then dissolved in ethyl acetate and filtered through a plug of silica gel to remove the catalyst and concentrated in vacuo. The crude product **12** was analyzed by ^1^H NMR and HPLC. Some products (high ees) were purified by column chromatography on silica gel.

## 4. Conclusions

Several novel chiral bifunctional hydrogen-bond donors were synthesized by applying the *Cinchona* alkaloid structures and a variety of selenoureas substituted moieties. We have demonstrated the first successful application of the obtained catalysts in a Michael addition of *S,S′*-diphenyl dithiomalonate to trans-β-nitrostyrene and a catalytic Michael–hemiacetalization reaction. The tested reactions, significantly differing in a nucleophile’s and electrophile’s structures, led to respective Michael adducts with excellent yields in a short time, generating the chiral products with up to 96% and 94% ees. Moreover, driving more sustainable processes, the application of catalysis under solvent-less conditions proved the effectiveness of the selenoureas in reactions performed in a ball mill. Hence, the products were obtained in comparable stereoselectivities and with higher yields than those generated at the solution. Albeit that the moderate enantioselectivities were achieved in the reaction of dimedone under mechanochemical conditions, the standard transformations in a solvent provided both high yields and stereoselectivities exceeding 90% ee. Therefore, we believe the proposed novel bifunctional chiral hydrogen-bonding systems would successfully complete the family of organocatalysts.

## Figures and Tables

**Table 1:** Stereoselective Michael addition of *S*,*S*′-diphenyl dithiomalonate **8** to trans-β-nitrostyrene **7** [6] ^a^.

Entry	Catalyst	Time (min)	Conv. (%) ^b^	ee (%) ^c^	Config. ^d^
1	*e*QN-**5a**	15	>99	87	*S*
2	*e*QN-**5b**	15	99 (94)	**96**	*S*
3	*e*QN-**5c**	30	99 (96)	95 (72) ^e^	*S*
4	*e*QN-**5d**	5	>99 (95)	**96** (78) ^f^	*S*
5	*e*QN-**5e**	30	>99 (97)	93 (69) ^e^	*S*
6	*e*CD-**5f**	30	>99	85	*S*
7	*e*DHQN-**5g**	15	99	86	*S*
8	*e*QD-**5h**	5	>99 (98)	**96** (87) ^f^	*R*
9	*e*QD-**5i**	5	99 (95)	94 (89) ^f^	*R*
10	*e*QD-**5j**	90	94	87 (72) ^e^	*R*
11	*e*DHQD-**5k**	15	89 (83)	94	*R*
12	*e*QN-**6a**	5	>99 (93)	95	*S*
13	*e*QN-**6b**	90	96	76	*S*

^a^ Reactions were performed at 273 K on a 0.1-mmol scale in toluene (0.5 mL). ^b^ Conversion was determined by ^1^H NMR of a crude reaction mixture. The isolated yield is given in parentheses. ^c^ The ee was determined by chiral HPLC analysis using a Chiralcel IB-3 column. ^d^ Determined by comparison with available literature HPLC data [24]. ^e^ Performed at 298 K for 15 min. ^f^ Performed at 298 K for 5 min.

**Table 2:** Asymmetric Michael addition of *S*,*S*′-diphenyl dithiomalonate **8** to trans-β-nitrostyrene **7** performed in planetary ball mill ^a^.

Entry	Catalyst	Conv. (%) ^b^	ee (%) ^c^	Config. ^d^
1	*e*QN-**5a**	96	73	*S*
2	*e*QN-**5b**	94	71	*S*
3	*e*QN-**5c**	98	71	*S*
4	*e*QN-**5d**	>99 (96)	77	*S*
5	*e*QN-**5e**	>99	69	*S*
6	*e*CD-**5f**	94	76	*S*
7	*e*DHQN-**5g**	93 (89)	90	*S*
8	*e*QD-**5h**	95 (95)	84	*R*
9	*e*QD-**5i**	98 (95)	**93**	*R*
10	*e*QD-**5j**	91 (87)	87	*R*
11	*e*DHQD-**5k**	95 (92)	**93**	*R*
12	*e*QN-**6a**	99	77	*S*
13	*e*QN-**6b**	>99	59	*S*

^a^ Reactions were performed on a 0.25-mmol scale with 5 mol% of the respective catalyst for 30 min. Milling setup: 2 balls × 10 mm (ball diameter). Milling speed: 400 rpm. ^b^ Conversion was determined by ^1^H NMR of a crude reaction mixture. The isolated yield is given in parentheses. ^c^ The ee was determined by chiral HPLC analysis using a Chiralcel IB-3 column. ^d^ Determined by comparison with available literature and HPLC data [24].

**Table 3:** Asymmetric Michael–hemiacetalization reaction of benzylidene pyruvate **10** and dimedone **11** [25] ^a^.

Entry	Catalyst	Conv. (%) ^b^	ee (%) ^c^	Config. ^d^
1	*e*QN-**5a**	>99	67	*R*
2	*e*QN-**5b**	>99	64	*R*
3	*e*QN-**5c**	>99	65 (69) ^e^	*R*
4	*e*QN-**5d**	>99 (95)	84 (76) ^e^	*R*
5	*e*QN-**5e**	>99 (97)	**94** (89) ^e^	*R*
6	*e*CD-5f	>99	66	*R*
7	*e*DHQN-**5g**	>99	69	*R*
8	*e*QD-**5h**	>99	73 (80) ^e^	*S*
9	*e*QD-**5i**	>99 (94)	85 (80) ^f^	*S*
10	*e*QD-**5j**	>99 (96)	**93** (85) ^f^	*S*
11	*e*DHQD-**5k**	>99 (93)	82	*S*
12	*e*QN-**6a**	>99 (94)	91	*R*
13	*e*QN-**6b**	>99	76	*R*

^a^ Reactions were performed at 298 K on a 0.1-mmol scale in toluene (0.5 mL) with 5 mol% of the respective catalyst for 30 min. ^b^ Conversion was determined by ^1^H NMR of a crude reaction mixture. The isolated yield is given in parentheses. ^c^ The ee was determined by chiral HPLC analysis using a Chiralcel IA-3 column. ^d^ Determined by comparison with available literature HPLC data [25]. ^e^ Performed at 273 K for 15 min. ^f^ Performed at 273 K for 30 min.

**Table 4:** Asymmetric Michael–hemiacetalization reaction of benzylidene pyruvate **10** and dimedone **11** performed in planetary ball mill ^a^.

Entry	Catalyst	Conv. (%) ^b^	ee (%) ^c^	Config. ^d^
1	*e*QN-**5a**	82	50	*R*
2	*e*QN-**5b**	82	35	*R*
3	*e*QN-**5c**	88	34	*R*
4	*e*QN-**5d**	67 (65)	58	*R*
5	*e*QN-**5e**	78	53	*R*
6	*e*CD-**5f**	92	50	*R*
7	*e*DHQN-***5g***	98	53	*R*
8	*e*QD-**5h**	32	39	*S*
9	*e*QD-**5i**	96 (94)	64	*S*
10	*e*QD-**5j**	78 (74)	**73**	*S*
11	*e*DHQD-**5k**	36	39	*S*
12	*e*QN-**6a**	38	48	*R*
13	*e*QN-**6b**	49 (46)	61	*R*

^a^ Reactions were performed on a 0.25-mmol scale with 5 mol% of the respective catalyst for 30 min. Milling setup: 2 balls × 10 mm (ball diameter). Milling speed: 400 rpm. ^b^ Conversion was determined by ^1^H NMR of a crude reaction mixture. The isolated yield is given in parentheses. ^c^ The ee was determined by chiral HPLC analysis using a Chiralcel IA-3 column. ^d^ Determined by comparison with available literature HPLC data [25].

## Data Availability

The data are available upon request from the corresponding author.

## Supplementary Materials

The following are available online. Figures S1–S8: copies of ^1^H and ^13^C NMR spectra, Figures S9–S74: HPLC plots for the Michael reactions.

## Sample Availability

Samples of the compounds **5a**–**k** and **6a**,**b** are available from the author (M.Z.-B).
