# Supplementary material for: Efficacy of Selenourea Organocatalysts in Asymmetric Michael Reactions under Standard and Solvent-Free Conditions
Source: Molecules. 2021 Dec 1;26(23):7303. doi: 10.3390/molecules26237303 (PMC8658871; doi:10.3390/molecules26237303)
Supplement: Supplementary file 1 [file molecules-26-07303-s001.zip › molecules-1475123-supplementary.pdf]

Supplementary Materials

Efficacy of Selenourea Organocatalysts in Asymmetric Michael Reactions under Standard and Solvent-Free Conditions

Mariola Zielińska-Błajet*, Żaneta A. Mała, Rafał Kowalczyk

Faculty of Chemistry, Wrocław University of Science and Technology, Wybrzeże Wyspiańskiego 27, 50-370 Wrocław, Poland;
*Correspondence: mariola.zielinska-blajet@pwr.edu.pl; Tel.: +48-71-3202128

Contents

S1. General..3

S2. Catalysts……………………………………………………………………………………3

S3. Spectral data…………………………………………………………………...….. 4

S4. HPLC chromatograms 9

S1. General

^{1}H and ^{13}C NMR spectra (400 and 125 MHz or 600 and 151 MHz) were recorded in $CDCl_3$ on a JEOL ECZ400S instrument (Jeol Ltd., Tokyo, Japan) and Bruker Avance II 600 instrument (Bruker, Billerica, MA, USA), respectively. ^{1}H NMR chemical shifts are reported in ppm (δ) relative to tetramethylsilane (TMS) and $CDCl_3$ (δ 7.26 ppm). Data are reported as follows: chemical shift, multiplicity (s = singlet, br s = broad singlet, d = doublet, t = triplet, q = quartet, m = multiplet, etc.), coupling constants (Hz) and integration.

^{13}C NMR chemical shifts are reported in ppm from tetramethylsilane (TMS) or $CDCl_3$ (δ 77.16 ppm). High resolution mass spectra (HRMS) were measured on a Waters LCT Premier XE TOF instrument (Waters Corporation, Milford, MA, USA) with electrospray ionization.

HPLC analysis was performed on SHIMADZU NEXERA X2 apparatus (Chiral Technologies INC. West Chester, PA, USA) using CHIRALPAK IA-3, IB-3 and IC-3 chiral columns (4.6 mm × 25 cm) without guard column. Each HPLC analysis has been controlled by comparison with the pure sample and the racemate.

Optical rotations were measured using an Optical Activity Ltd. Model AA-5 automatic polarimeter (Optical Activity Ltd., Huntington, UK) at λ = 589 nm (c g/100 mL).

Melting points were determined using a Boëtius hot-stage apparatus (PHMK VEB Analytic, Dresden, Germany).

Reactions were monitored by thin-layer chromatography (TLC) on silica gel 60 F-254 precoated plates (Merck, Darmstadt, Germany), and spots were visualized with a UV lamp or KMnO4 dip.

Catalytic reactions were performed in standard reaction tubes with PTFE stopper without any protection against moisture or air.

Ball milling was realized in a planetary ball mill PM-200 (Retsch GmbH, Haan, Germany) in a stainless steel 12 mL containers with stainless steel grinding balls with diameter 10 mm.

S2. Catalysts

Selenoureas **5a–c**, **5f–h**, **5k** and thiourea **6a** were described in our previous publication [7]. Thiourea **6b** were prepared following the literature precedents [40,41]. Compounds **5d**, **5e**, **5i** and **5j** are novel.

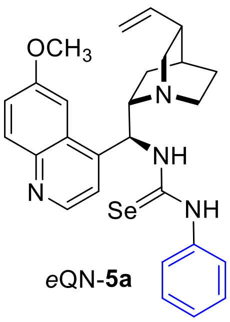

*e*QN-**5a**

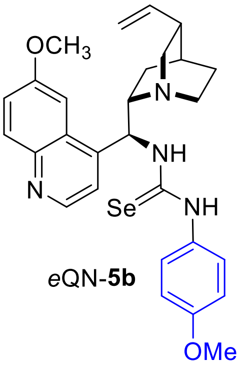

*e*QN-**5b**

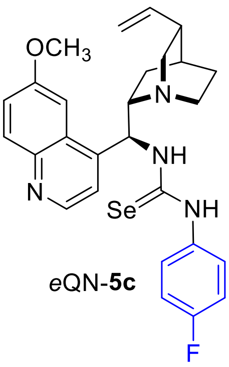

*e*QN-**5c**

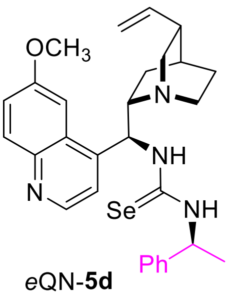

*e*QN-**5d**

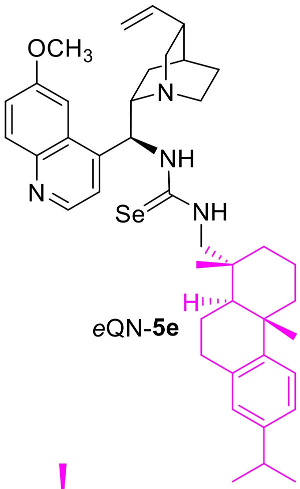

*e*QN-**5e**

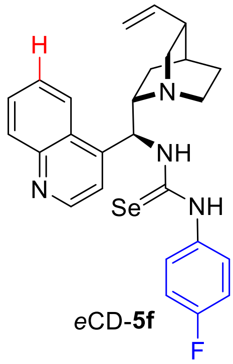

*e*CD-**5f**

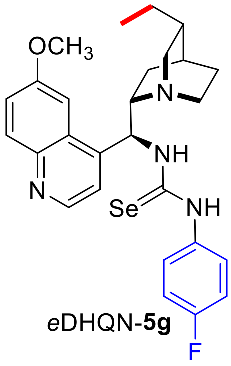

*e*DHQN-**5g**

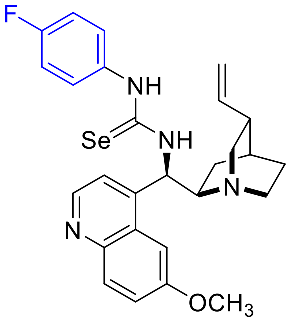

*e*QD-**5h**

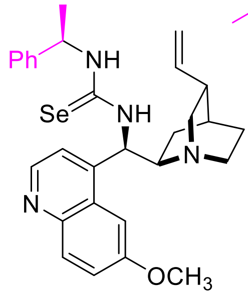

*e*QD-**5i**

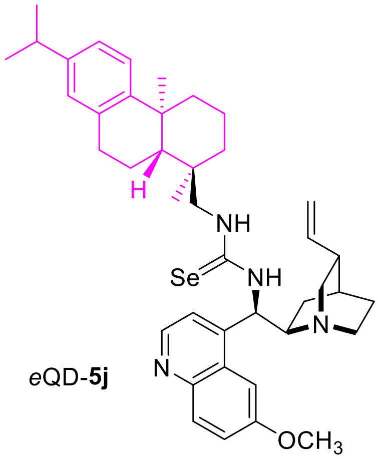

*e*QD-**5j**

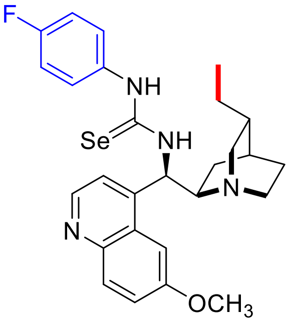

*e*DHQD-**5k**

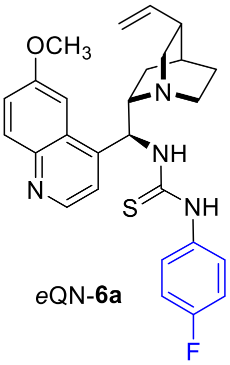

*e*QN-**6a**

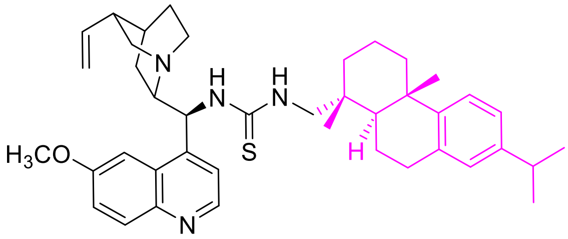

*e*QN-**6b**

S3. Spectral data

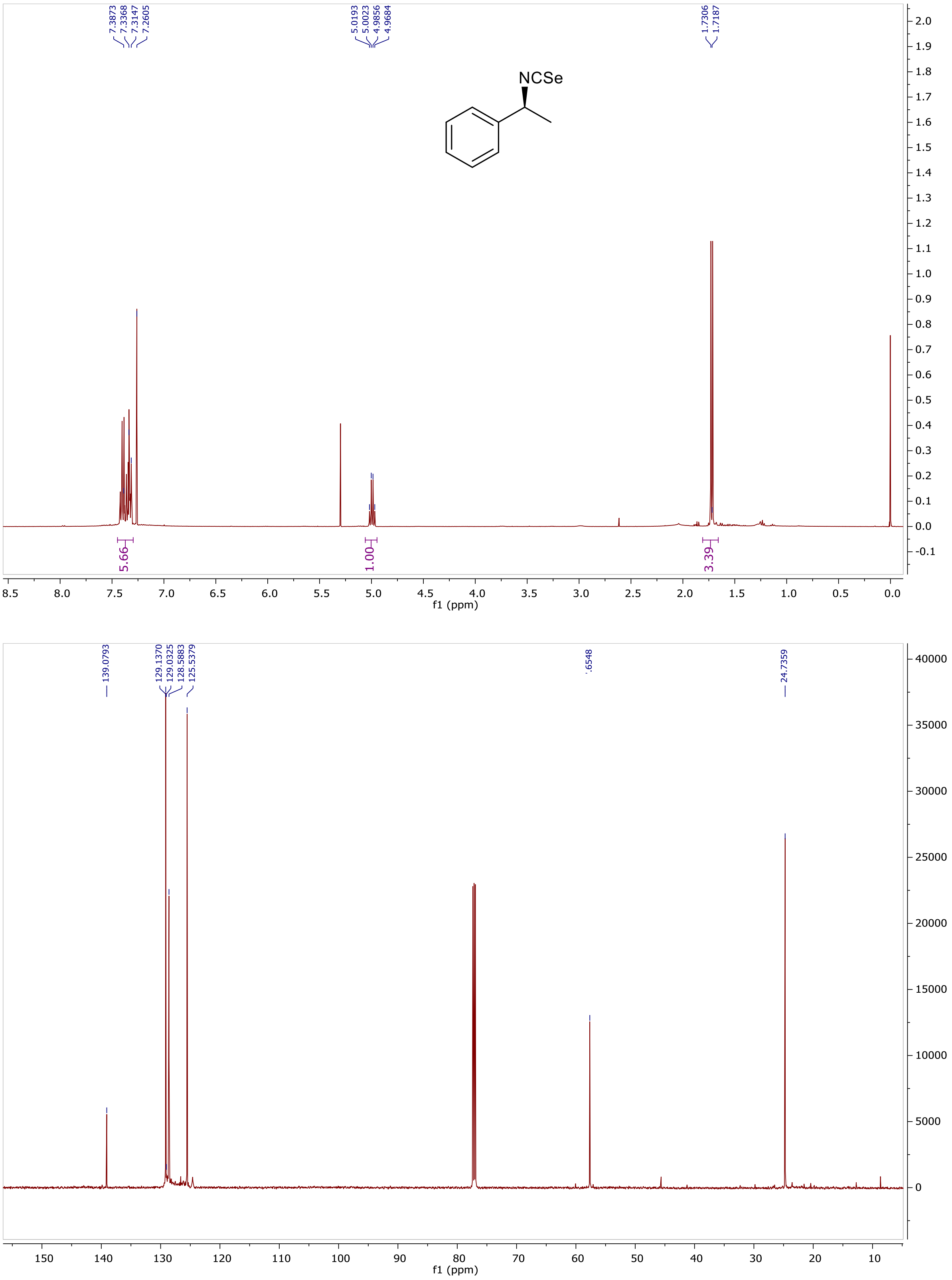

Figure S1. ^{1}H and ^{13}C NMR spectra of [(1*S*)-1-isoselenocyanatoethyl]benzene **3d**.

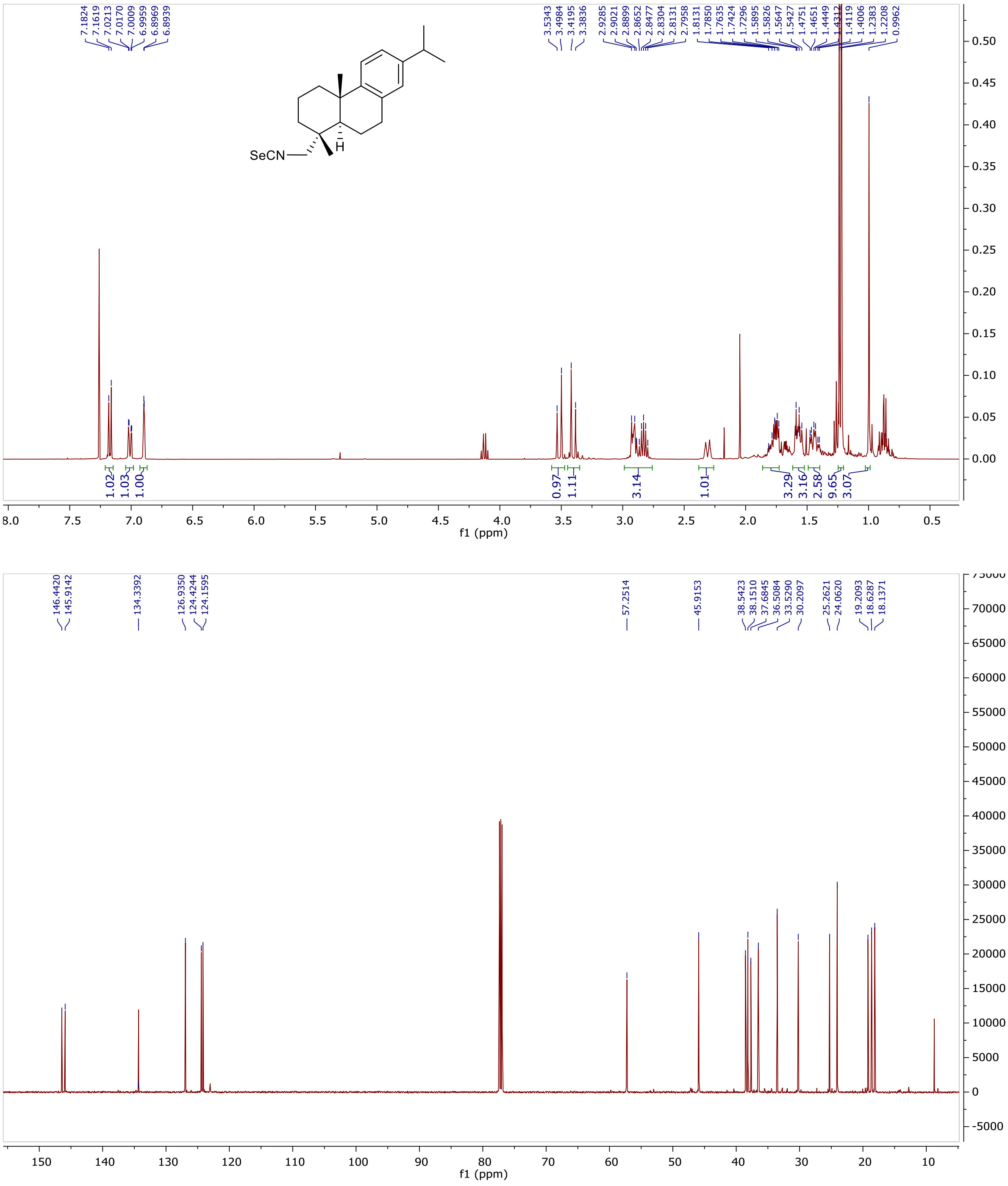

Figure S2. ^{1}H and ^{13}C NMR spectra of (1*R*,4a*S*,10a*R*)-1,4a-dimethyl-7-isopropyl-1-(isoselenocyanatomethyl)-1,2,3,4,4a,9,10,10a-octahydrophenanthrene **3e**.

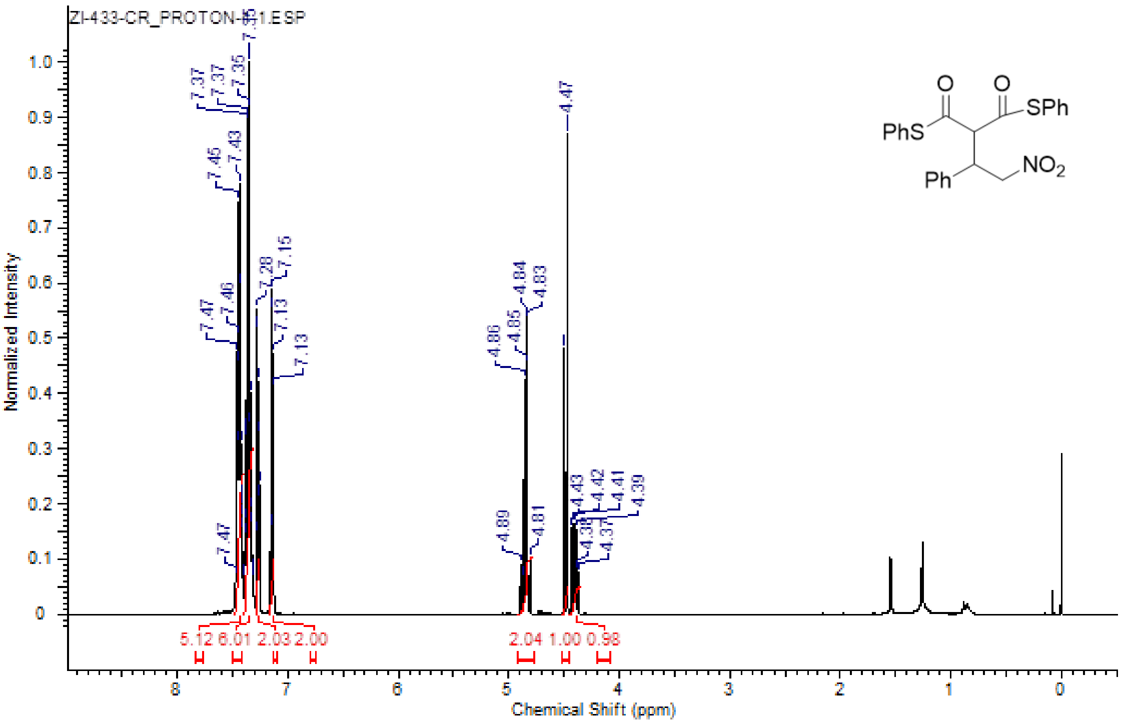

Figure S3. ^{1}H NMR (400 MHz) spectrum of 2-(2-nitro-1-phenylethyl)-1,3-bis(phenylsulfanyl)propane-1,3-dione **9** in $CDCl_3$. The signal at 1.26 ppm refers to residual hexane and the signal at 1.56 ppm refers to residual water.

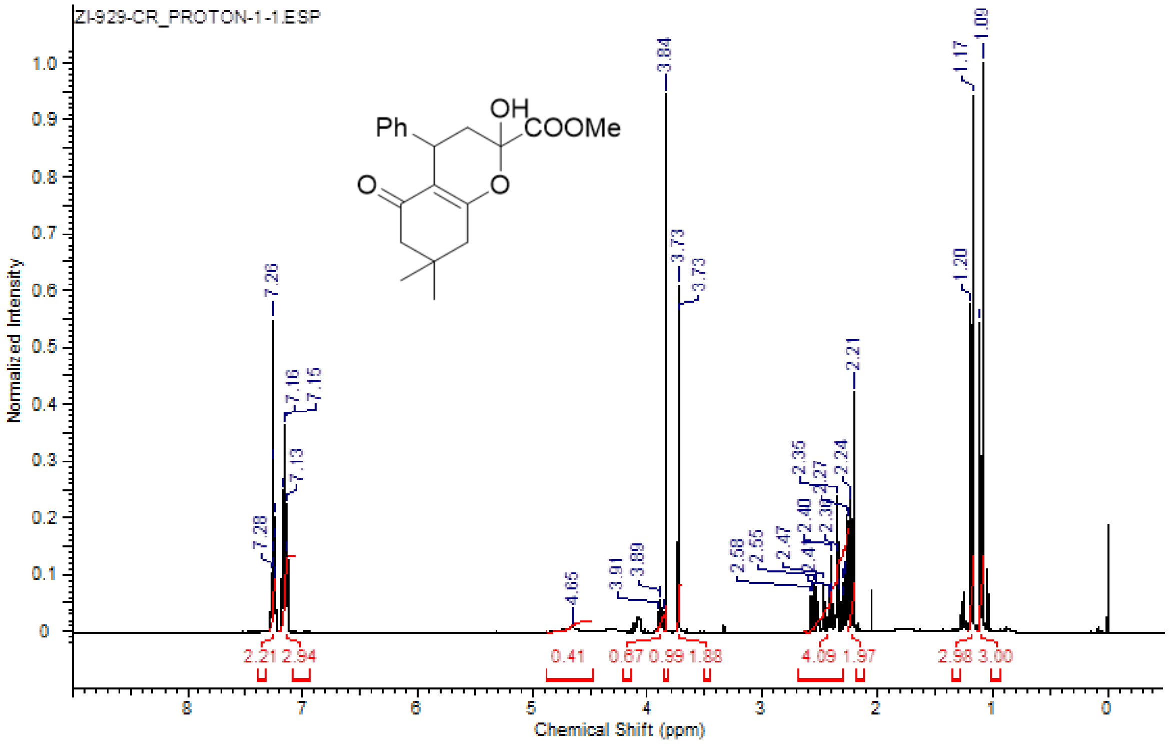

Figure S4. ^{1}H NMR (400 MHz) spectra of 4-methyl-2-hydroxy-7,7-dimethyl-5-oxo-4-phenyl-3,4,5,6,7,8-hexahydro-2*H*-chromene-2-carboxylate **12** in $CDCl_3$. The signal at 2.04 ppm refers to residual ethyl acetate.

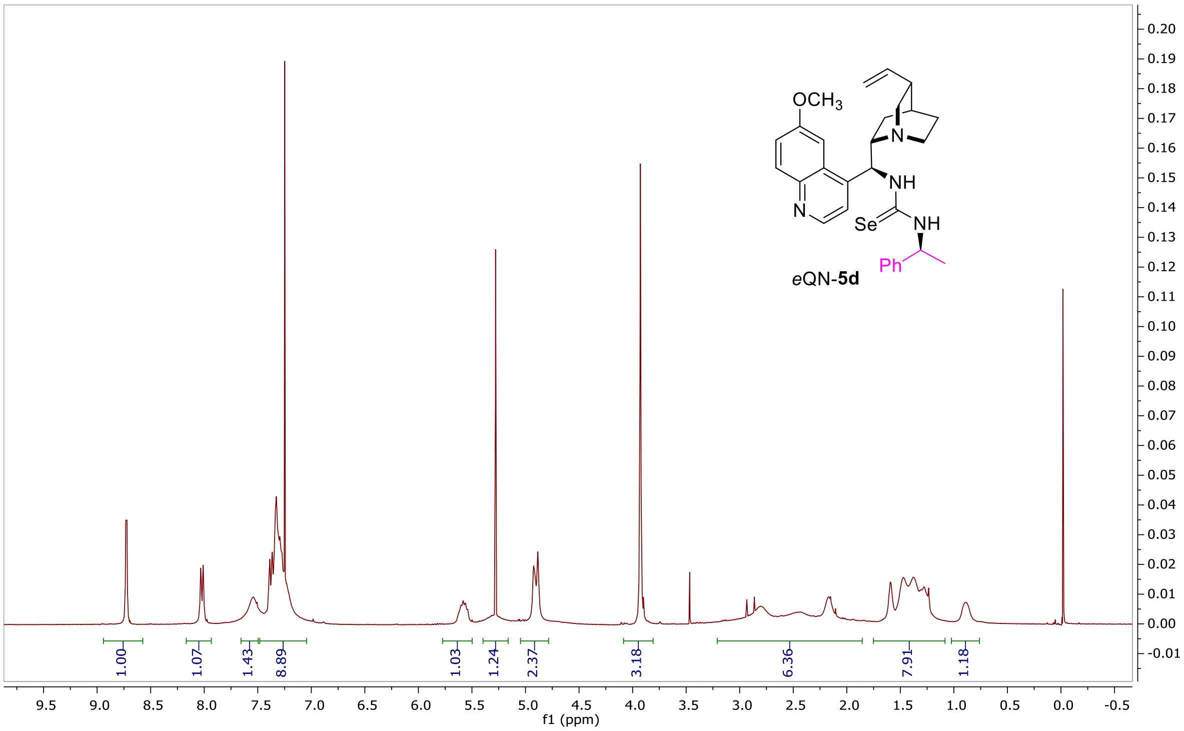

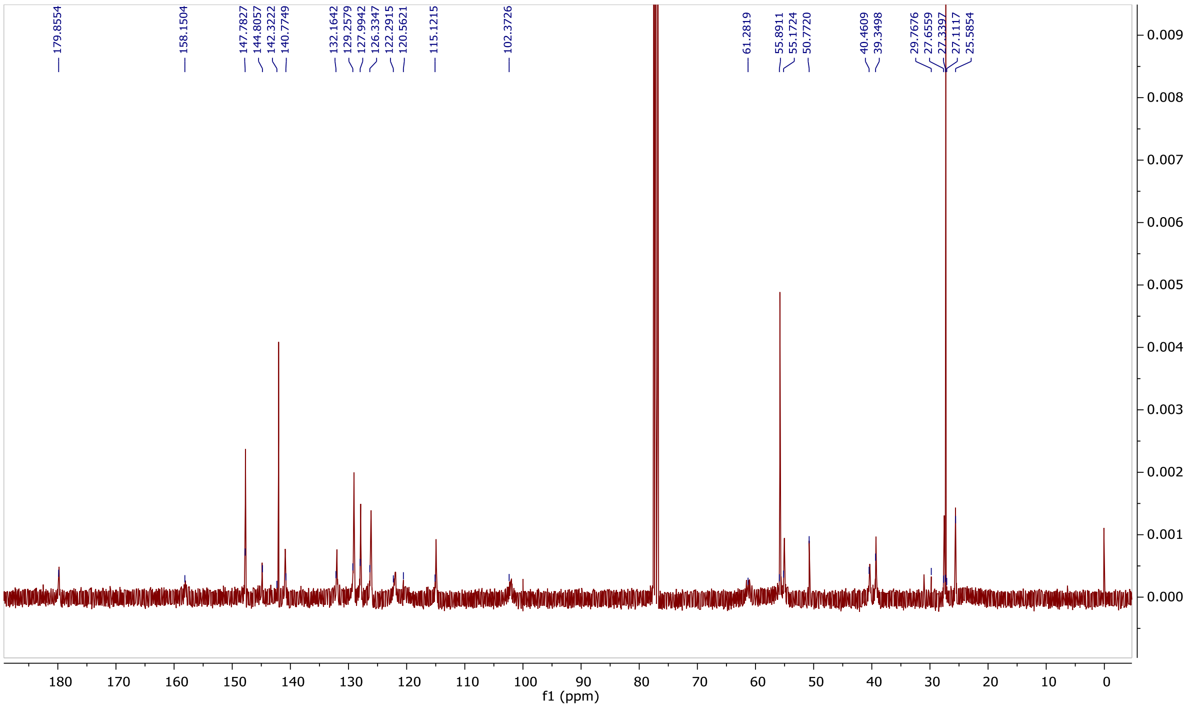

Figure S5. ^{1}H and ^{13}C NMR spectra of *N*-[(8*S*,9*S*)-6′-methoxycinchonan-9-yl]-*N*′-[(*S*)-1-phenylethyl]selenourea *e*QN-**5d.**

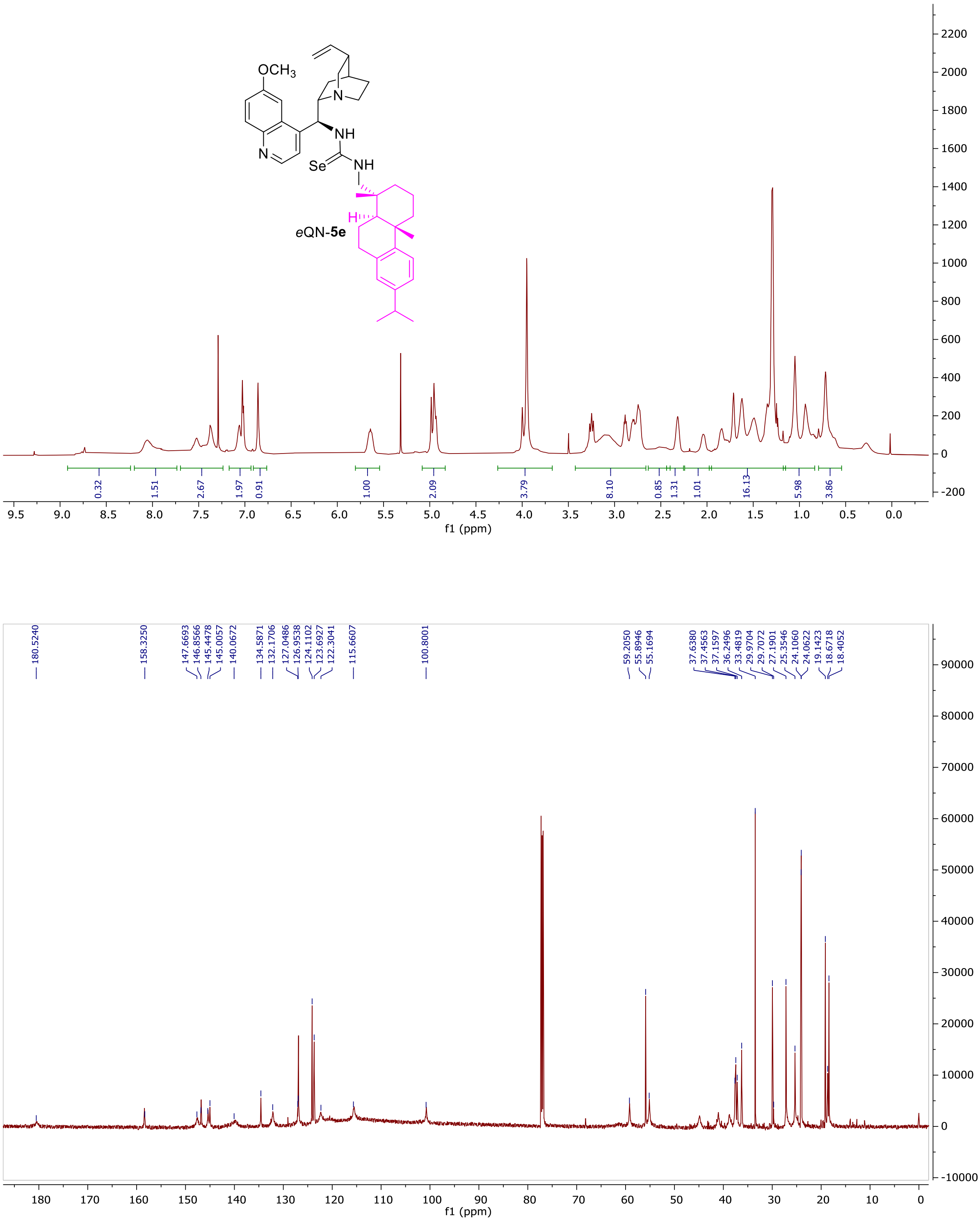

Figure S6. ^{1}H and ^{13}C NMR spectra of *N*-{[(1*R*,4a*S*,10a*R*)-1,4a-dimethyl-7-izopropyl-1,2,3,4,4a,9,10,10a-octahydrophenanthrene-1-yl]methyl}-*N′*-[(8*S*,9*S*)-6′-methoxycinchonan-9-yl]selenourea *e*QN-**5e.**

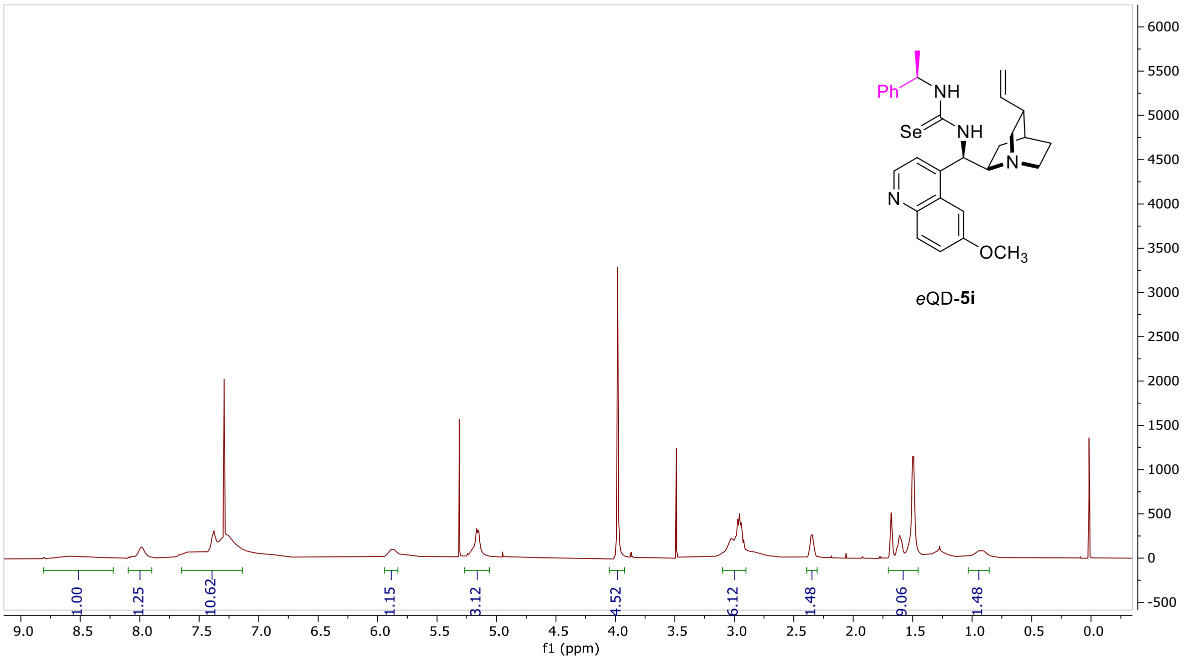

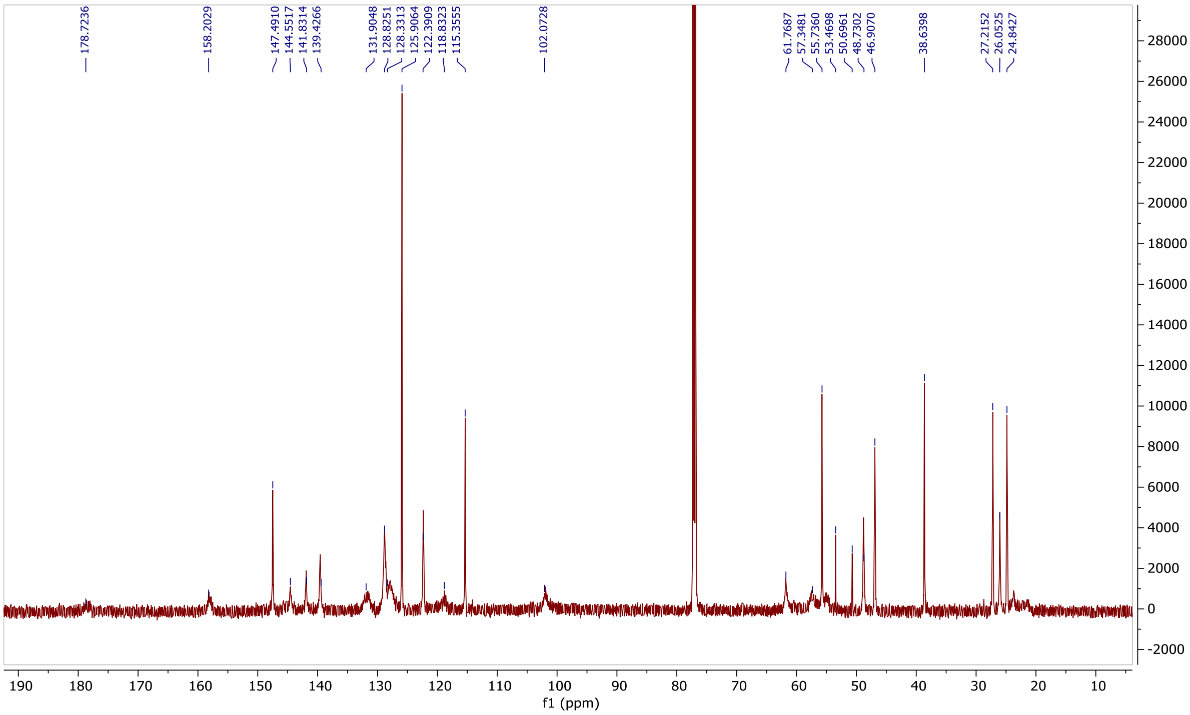

Figure S7. ^{1}H and ^{13}C NMR spectra of *N*-[(8*R*,9*R*)-6′-methoxycinchonan-9-yl]-*N*′-[(*S*)-1-phenylethyl]selenourea *e*QD-**5i.**

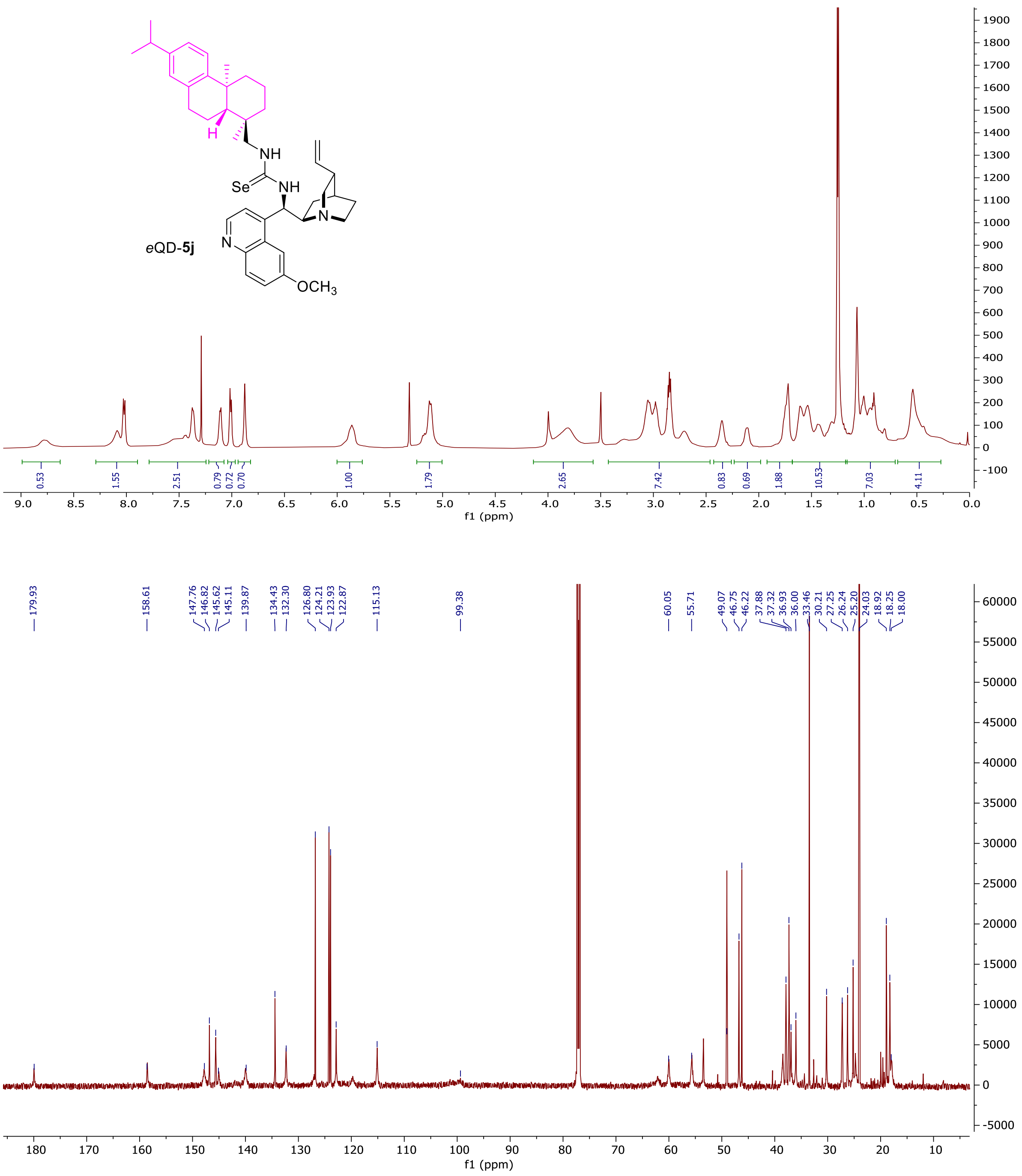

Figure S8. ^{1}H and ^{13}C NMR spectra of *N*-{[(1*R*,4a*S*,10a*R*)-1,4a-dimethyl-7-izopropyl-1,2,3,4,4a,9,10,10a-octahydrophenanthrene-1-yl]methyl}-*N'*-[(8*R*,9*R*)-6'-methoxycinchonan-9-yl]selenourea *e*QD-**5j.**

S4. HPLC chromatograms

S4.1.Michael Addition of S, S'-diphenyl dithiomalonate to trans-β-nitrostyrene.

(Chiralcel IB-3, *n*-hexane/2-propanol, 9:1, flow rate 1mL/min, λ = 205 nm)

<Chromatogram>

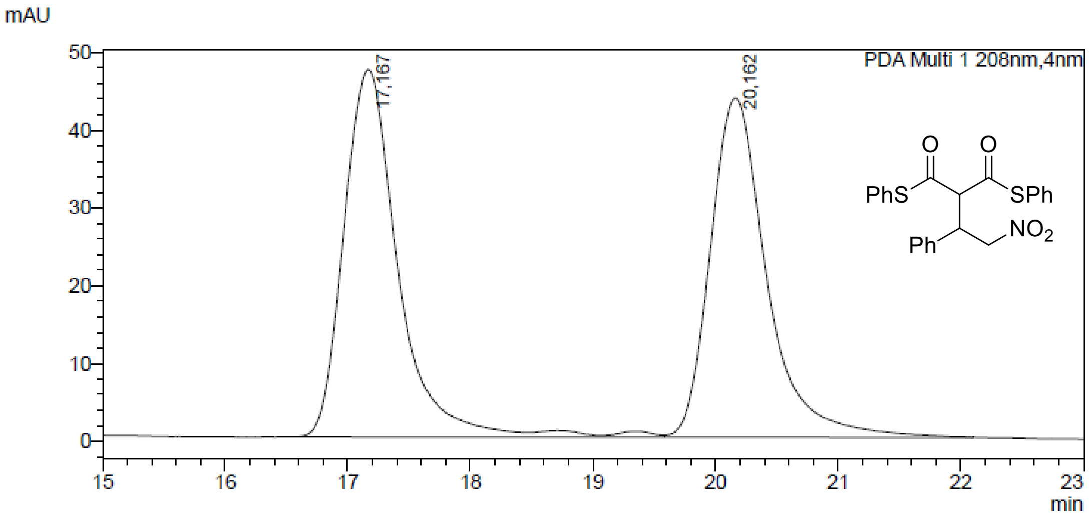

<Peak Table>

PDA Ch1 208nm

Peak#	Ret. Time	Area	Height	Area%
1	17,167	1451949	47230	50,915
2	20,162	1399774	43618	49,085
Total		2851722	90848	100,000

Figure S9. HPLC chromatogram for racemic 2-(2-nitro-1-phenylethyl)-1,3-bis(phenylsulfanyl)propane-1,3-dione **9**.

<Chromatogram>

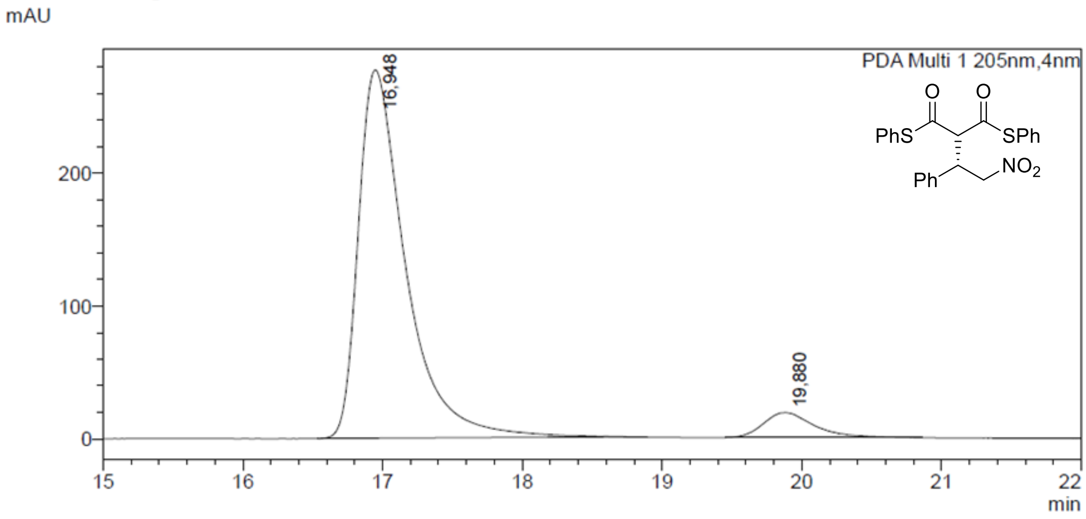

<Peak Table>

PDA Ch1 205nm

Peak#	Ret. Time	Height	Area	Area%
1	16,948	277480	6540106	93,241
2	19,880	18800	474053	6,759
Total		296281	7014159	100,000

Figure S10. HPLC chromatogram for *S*-**9**: sample obtained with catalyst *e*QN-**5a** in a solution at 273 K (87% ee).

<Chromatogram>

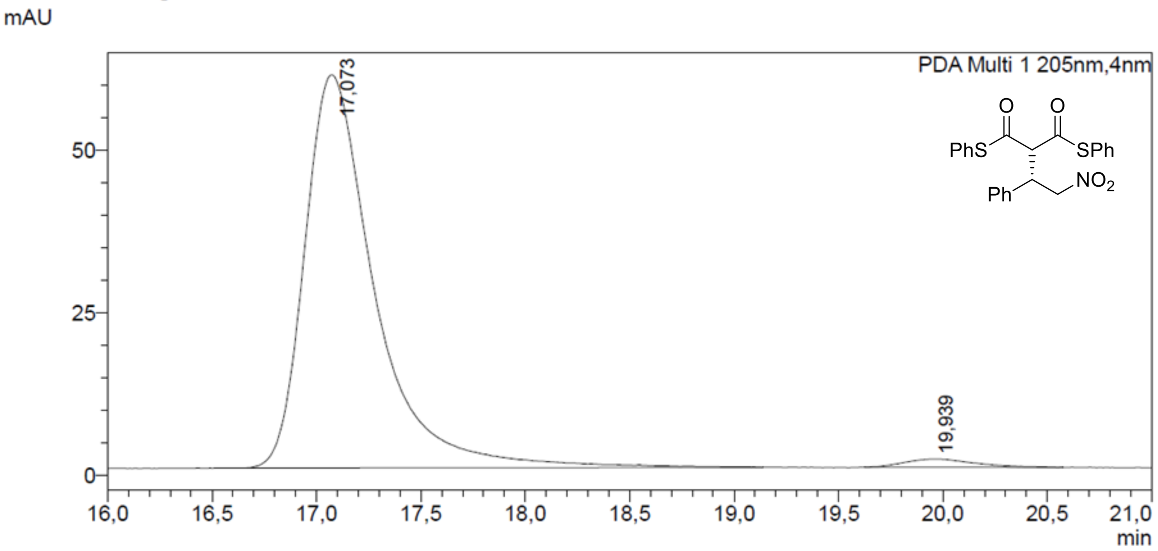

<Peak Table>

PDA Ch1 205nm

Peak#	Ret. Time	Height	Area	Area%
1	17,073	60341	1405426	98,020
2	19,939	1245	28389	1,980
Total		61587	1433816	100,000

Figure S11. HPLC chromatogram for *S*-**9**: sample obtained with catalyst *e*QN-**5b** in a solution at 273 K (96% ee).

<Chromatogram>

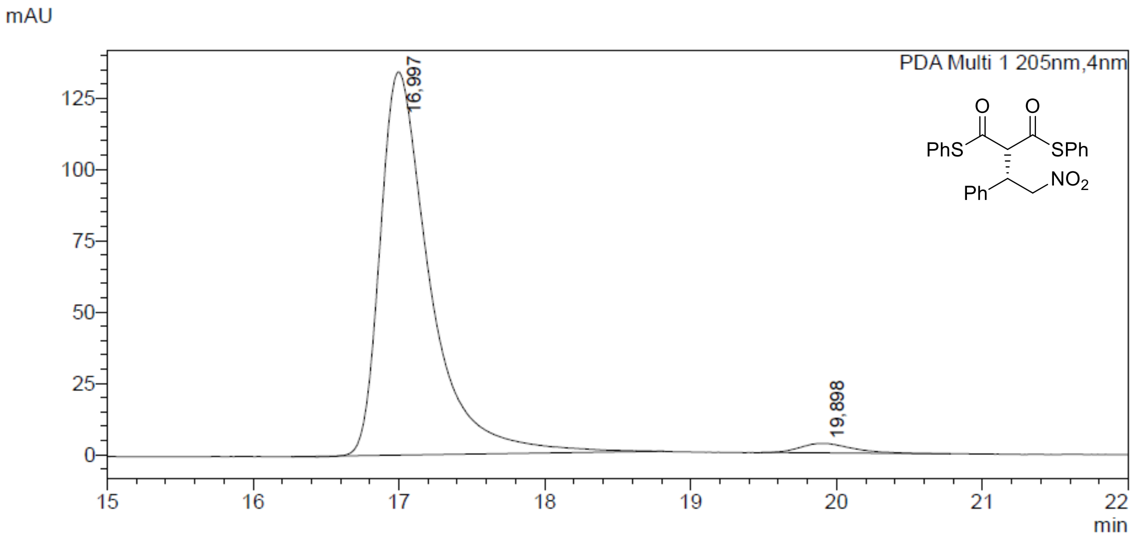

<Peak Table>

PDA Ch1 205nm

Peak#	Ret. Time	Height	Area	Area%
1	16,997	134255	3187870	97,395
2	19,898	3368	85276	2,605
Total		137622	3273146	100,000

Figure S12. HPLC chromatogram for *S*-**9**: sample obtained with catalyst *e*QN-**5c** in a solution at 273 K (95% ee).

<Chromatogram>

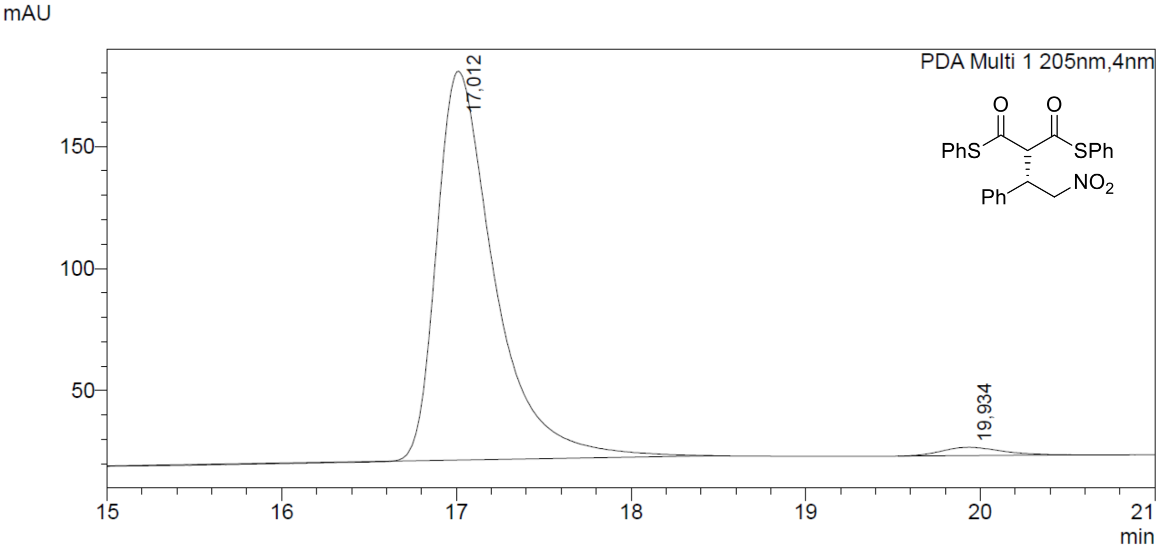

<Peak Table>

PDA Ch1 205nm

Peak#	Ret. Time	Height	Area	Area%
1	17,012	159270	3647067	97,839
2	19,934	3420	80554	2,161
Total		162689	3727621	100,000

Figure S13. HPLC chromatogram for *S*-**9**: sample obtained with catalyst *e*QN-**5d** in a solution at 273 K (96% ee).

<Chromatogram>

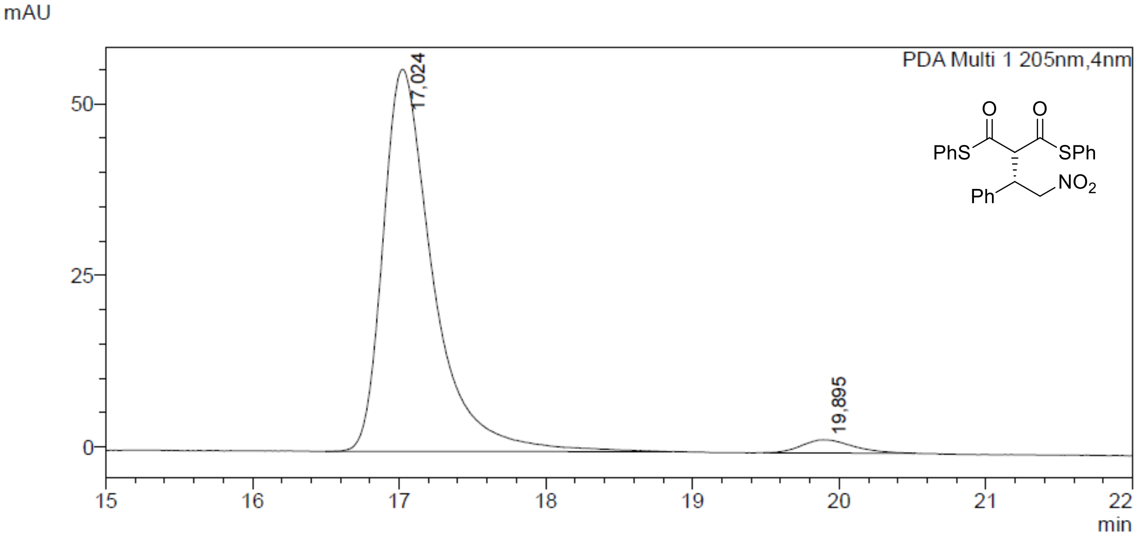

<Peak Table>

PDA Ch1 205nm

Peak#	Ret. Time	Height	Area	Area%
1	17,024	55701	1321649	96,572
2	19,895	1918	46921	3,428
Total		57619	1368570	100,000

Figure S14. HPLC chromatogram for *S*-**9**: sample obtained with catalyst *e*QN-**5e** in a solution at 273 K (93% ee).

<Chromatogram>

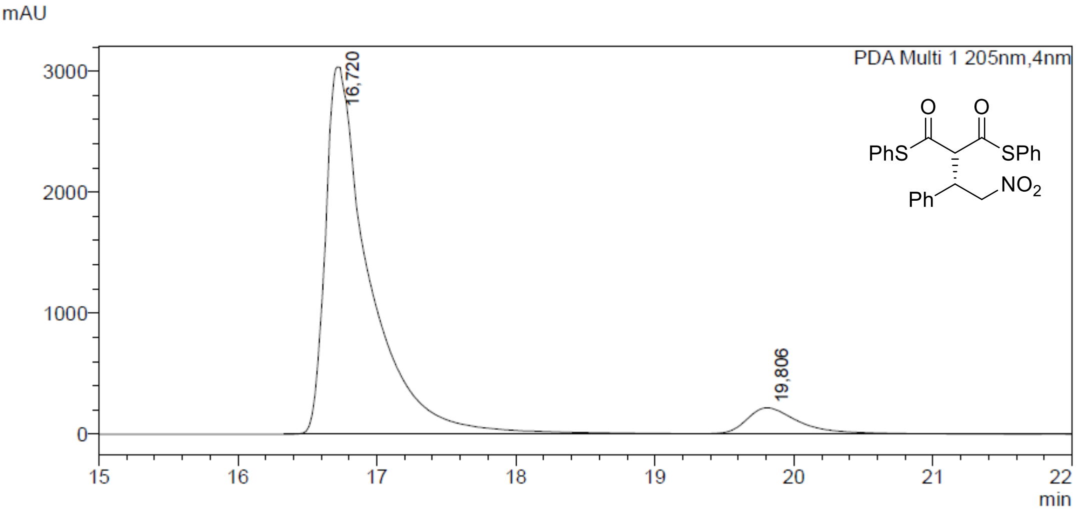

<Peak Table>

PDA Ch1 205nm

Peak#	Ret. Time	Height	Area	Area%
1	16,720	3033117	67223172	92,391
2	19,806	213659	5536411	7,609
Total		3246776	72759584	100,000

Figure S15. HPLC chromatogram for *S*-**9**: sample obtained with catalyst *e*CD-**5f** in a solution at 273 K (85% ee).

<Chromatogram>

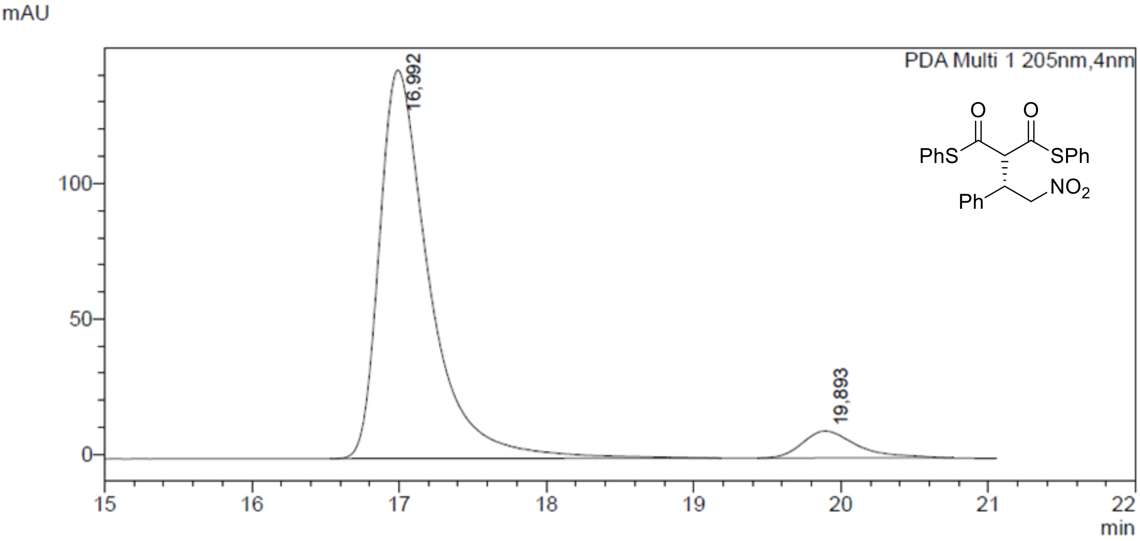

<Peak Table>

PDA Ch1 205nm

Peak#	Ret. Time	Height	Area	Area%
1	16,992	143305	3377740	92,939
2	19,893	9946	256605	7,061
Total		153251	3634345	100,000

Figure S16. HPLC chromatogram for *S*-**9**: sample obtained with catalyst *e*DHQN-**5g** in a solution at 273 K (86% ee).

<Chromatogram>

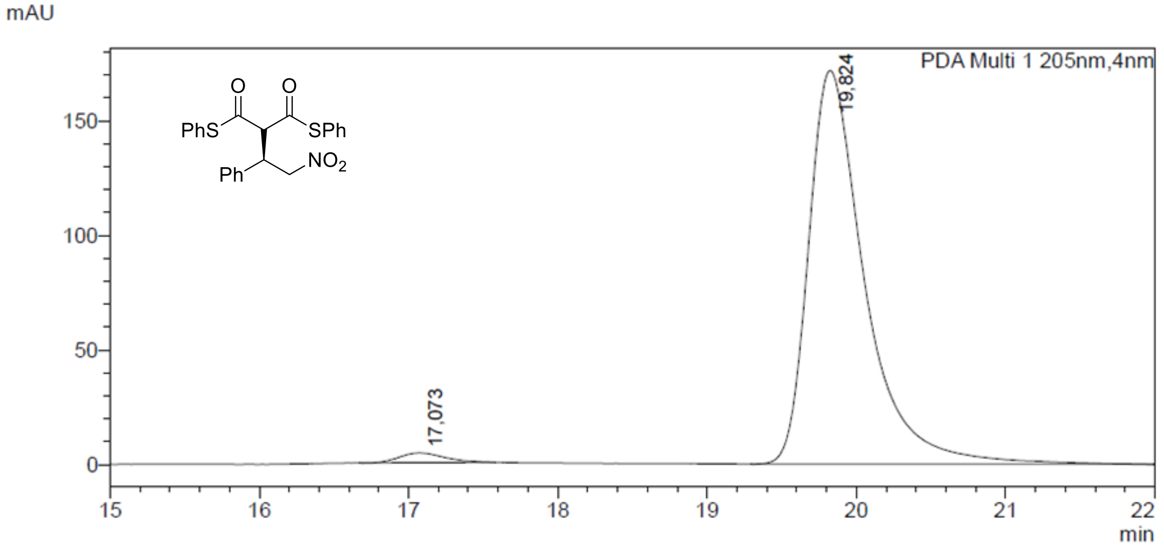

<Peak Table>

PDA Ch1 205nm

Peak#	Ret. Time	Height	Area	Area%
1	17,073	4291	90214	1,968
2	19,824	171787	4492951	98,032
Total		176078	4583165	100,000

Figure S17. HPLC chromatogram for *R*-**9**: sample obtained with catalyst *e*QD-**5h** in a solution at 273 K (96% ee).

<Chromatogram>

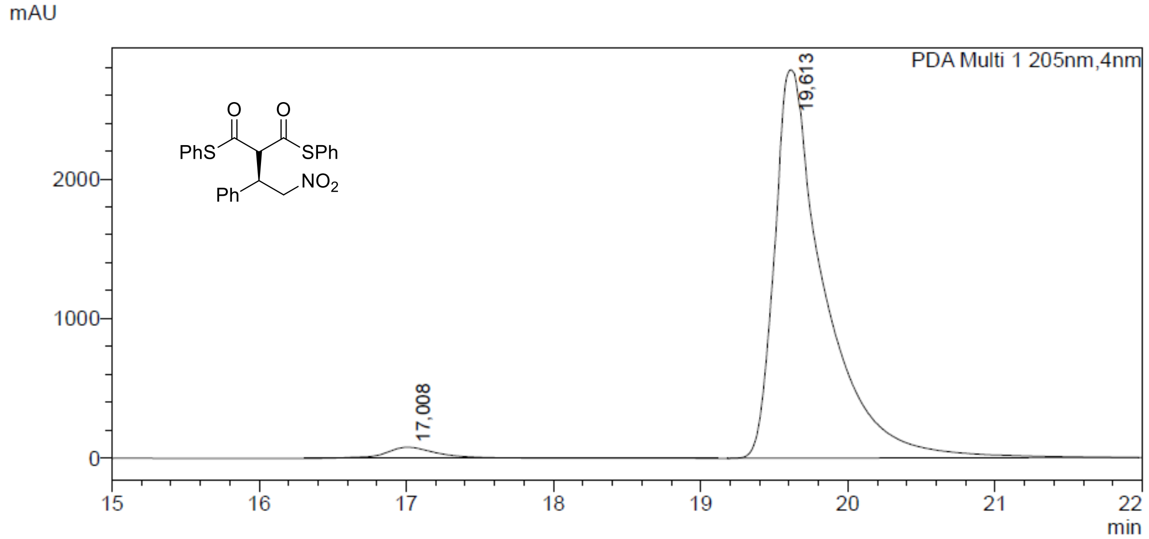

<Peak Table>

PDA Ch1 205nm

Peak#	Ret. Time	Height	Area	Area%
1	17,008	78936	1974337	2,913
2	19,613	2785797	65808578	97,087
Total		2864733	67782915	100,000

Figure S18. HPLC chromatogram for *R*-**9**: sample obtained with catalyst *e*QD-**5i** in a solution at 273 K (94% ee).

<Chromatogram>

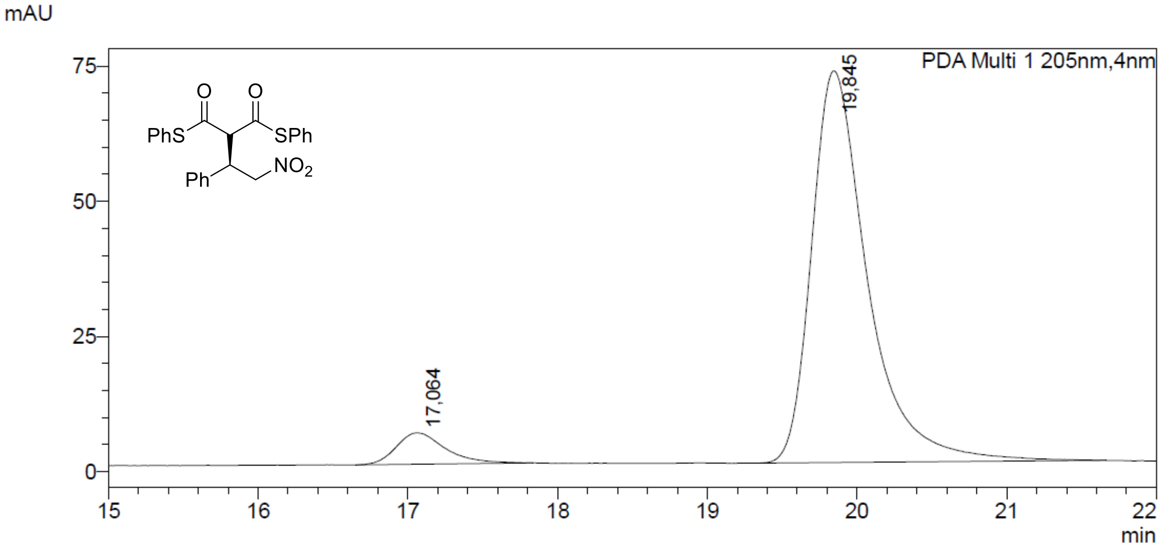

<Peak Table>

PDA Ch1 205nm

Peak#	Ret. Time	Height	Area	Area%
1	17,064	5818	132206	6,546
2	19,845	72434	1887444	93,454
Total		78253	2019650	100,000

Figure S19. HPLC chromatogram for *R*-**9**: sample obtained with catalyst *e*QD-**5j** in a solution at 273 K (87% ee).

<Chromatogram>

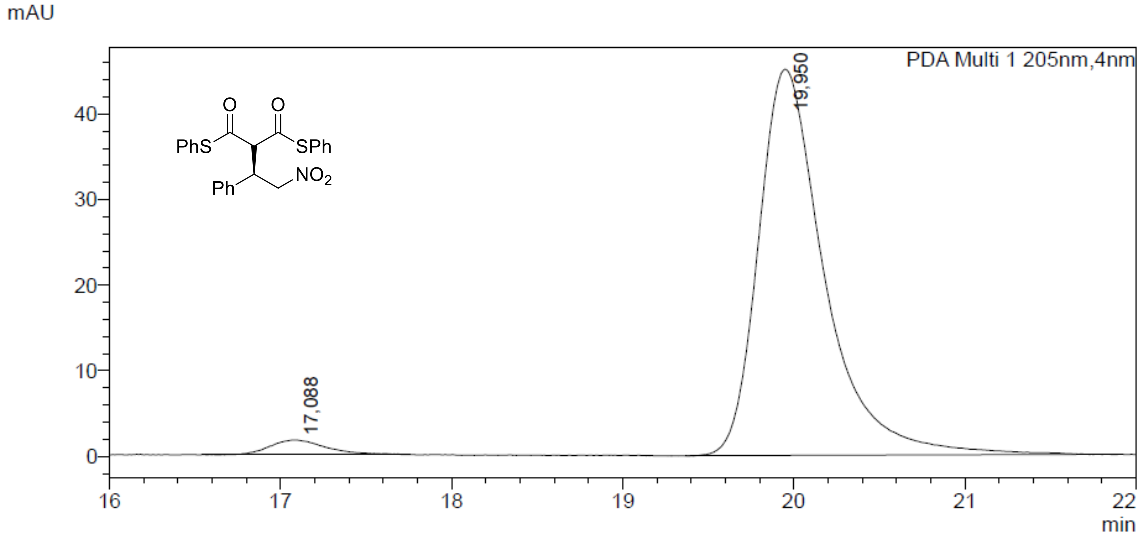

<Peak Table>

PDA Ch1 205nm

Peak#	Ret. Time	Height	Area	Area%
1	17,088	1708	39236	3,124
2	19,950	45123	1216567	96,876
Total		46831	1255803	100,000

Figure S20. HPLC chromatogram for *R*-**9**: sample obtained with catalyst *e*DHQD-**5k** in a solution at 273 K (94% ee).

<Chromatogram>

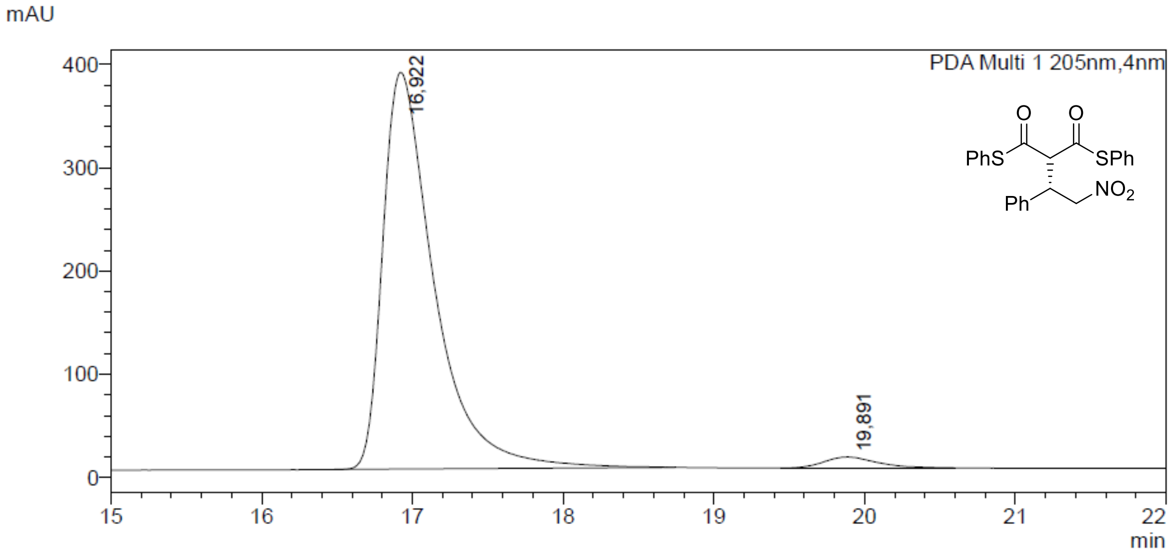

<Peak Table>

PDA Ch1 205nm

Peak#	Ret. Time	Height	Area	Area%
1	16,922	384266	9123943	97,228
2	19,891	10637	260096	2,772
Total		394903	9384039	100,000

Figure S21. HPLC chromatogram for *S*-**9**: sample obtained with catalyst *e*QN-**6a** in a solution at 273 K (95% ee).

<Chromatogram>

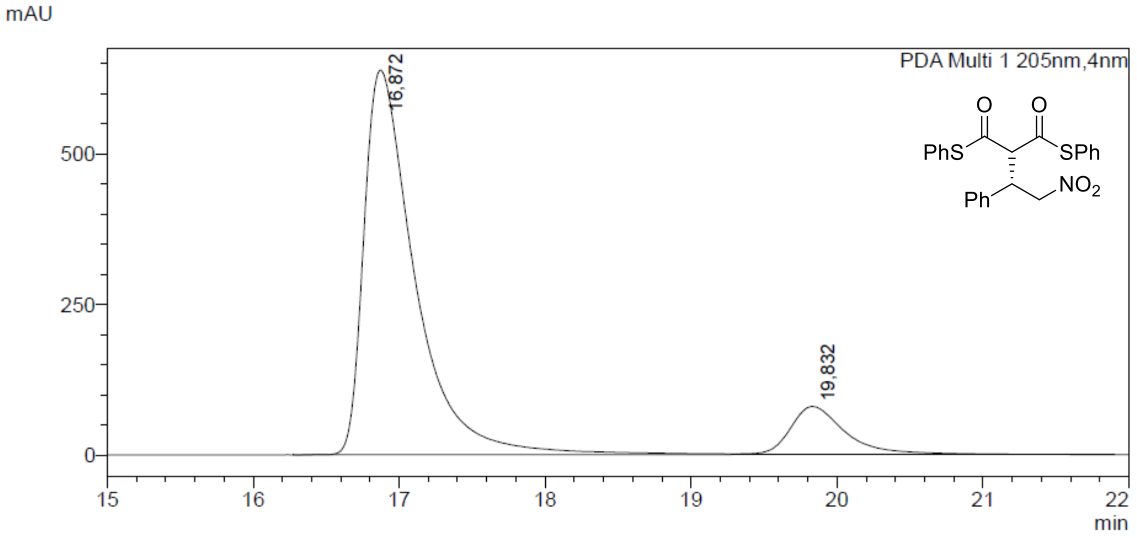

<Peak Table>

PDA Ch1 205nm

Peak#	Ret. Time	Height	Area	Area%
1	16,872	637452	15566419	87,961
2	19,832	79798	2130531	12,039
Total		717251	17696949	100,000

Figure S22. HPLC chromatogram for *S*-**9**: sample obtained with catalyst *e*QN-**6b** in a solution at 273 K (76% ee).

<Chromatogram>

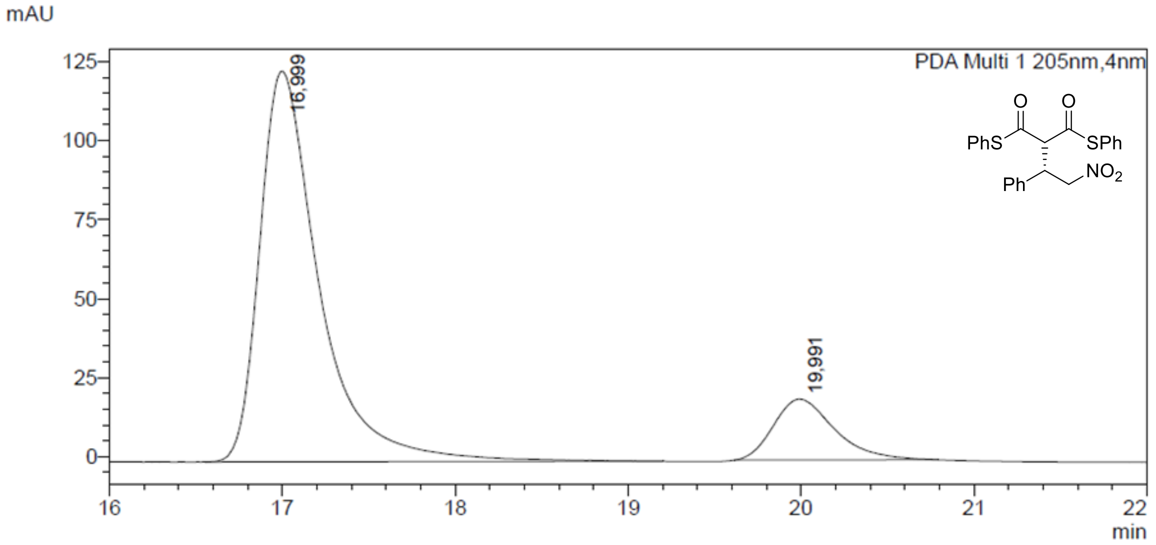

<Peak Table>

PDA Ch1 205nm

Peak#	Ret. Time	Height	Area	Area%
1	16,999	123587	2958165	86,201
2	19,991	19270	473529	13,799
Total		142857	3431694	100,000

Figure S23. HPLC chromatogram for *S*-**9**: sample obtained with catalyst *e*QN-**5c** in a solution at 298 K (72% ee).

<Chromatogram>

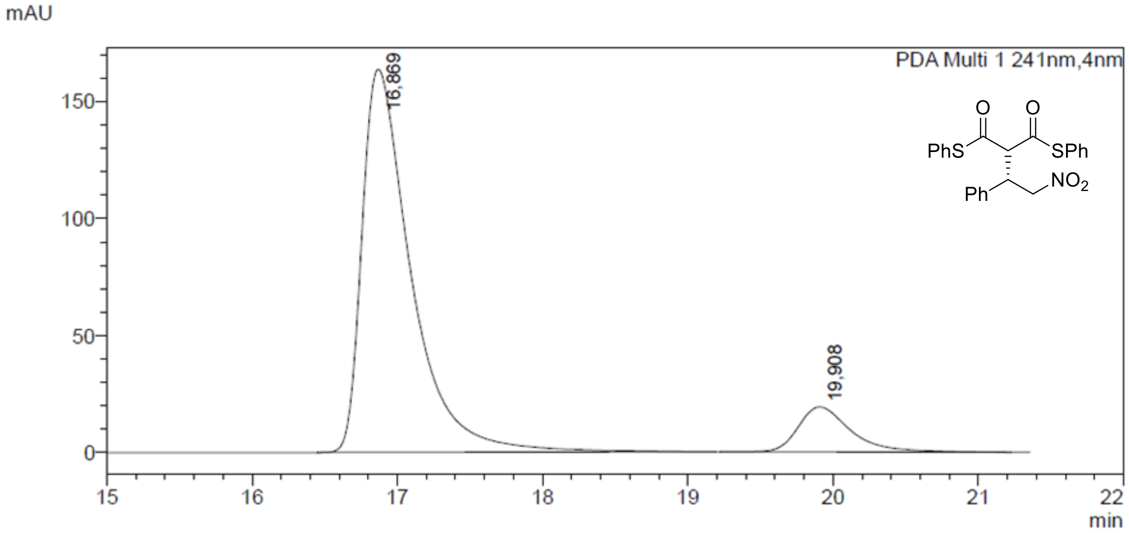

<Peak Table>

PDA Ch1 241nm

Peak#	Ret. Time	Height	Height%	Area	Area%
1	16,869	163734	89,492	3847695	88,827
2	19,908	19225	10,508	483955	11,173
Total		182959	100,000	4331650	100,000

Figure S24. HPLC chromatogram for *S*-**9**: sample obtained with catalyst *e*QN-**5d** in a solution at 298 K (78% ee).

<Chromatogram>

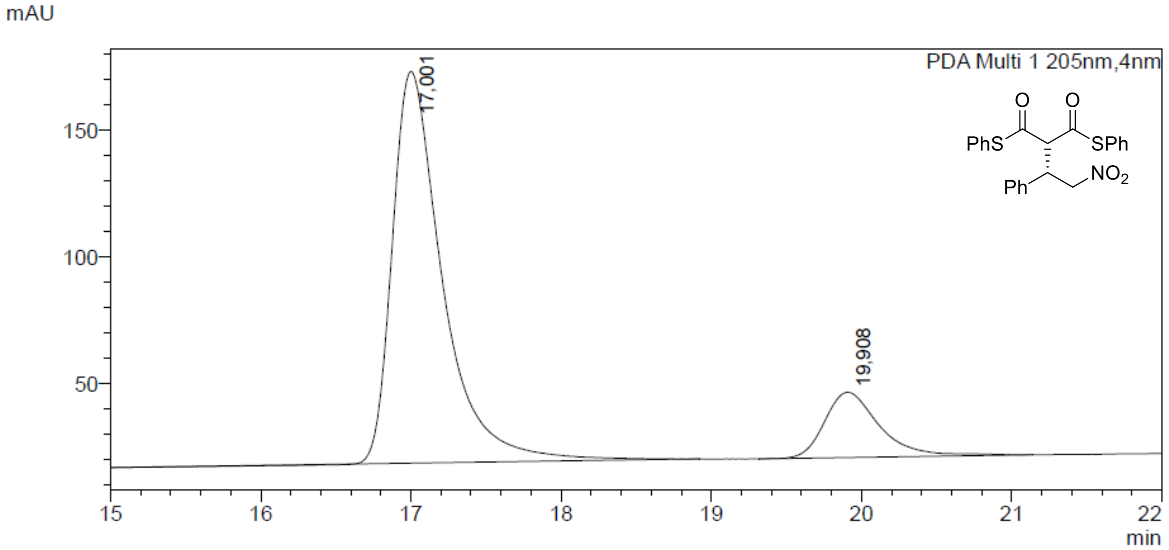

<Peak Table>

PDA Ch1 205nm

Peak#	Ret. Time	Height	Area	Area%
1	17,001	154644	3589373	84,520
2	19,908	25780	657379	15,480
Total		180424	4246751	100,000

Figure S25. HPLC chromatogram for *S*-**9**: sample obtained with catalyst *e*QN-**5e** in a solution at 298 K (69% ee).

<Chromatogram>

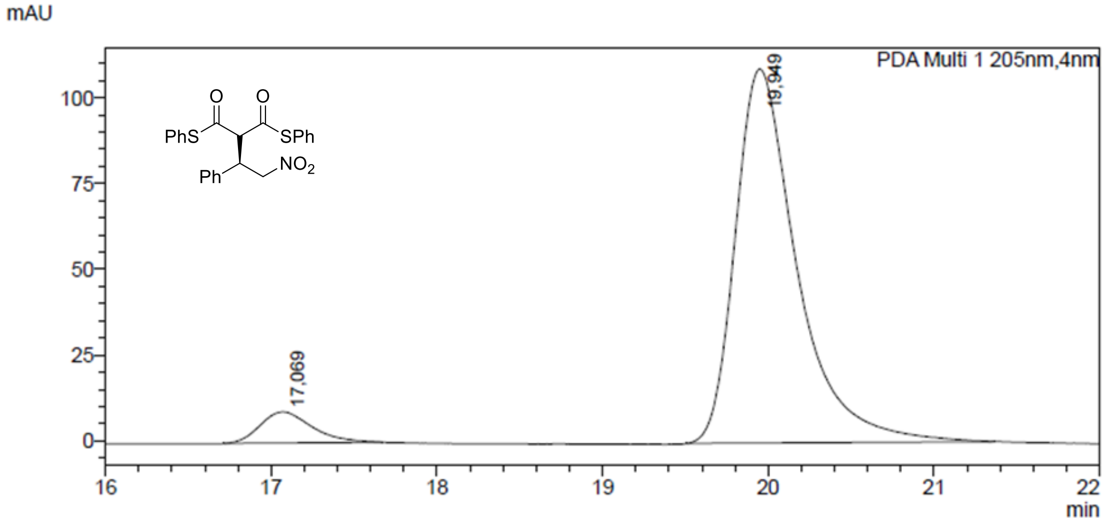

<Peak Table>

PDA Ch1 205nm

Peak#	Ret. Time	Height	Area	Area%
1	17,069	9045	197039	6,535
2	19,949	109101	2817944	93,465
Total		118147	3014983	100,000

Figure S26. HPLC chromatogram for *R*-**9**: sample obtained with catalyst *e*QD-**5h** in a solution at 298 K (87% ee).

<Chromatogram>

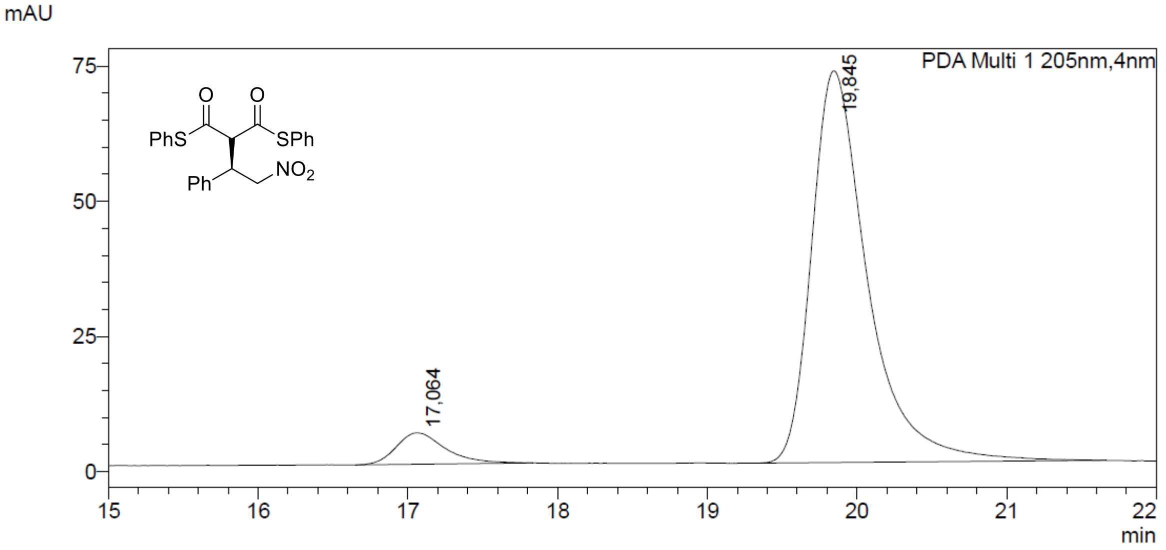

<Peak Table>

PDA Ch1 205nm

Peak#	Ret. Time	Height	Area	Area%
1	17,064	5818	132206	6,546
2	19,845	72434	1887444	93,454
Total		78253	2019650	100,000

Figure S27. HPLC chromatogram for *R*-**9**: sample obtained with catalyst *e*QD-**5i** in a solution at 298 K (89% ee).

<Chromatogram>

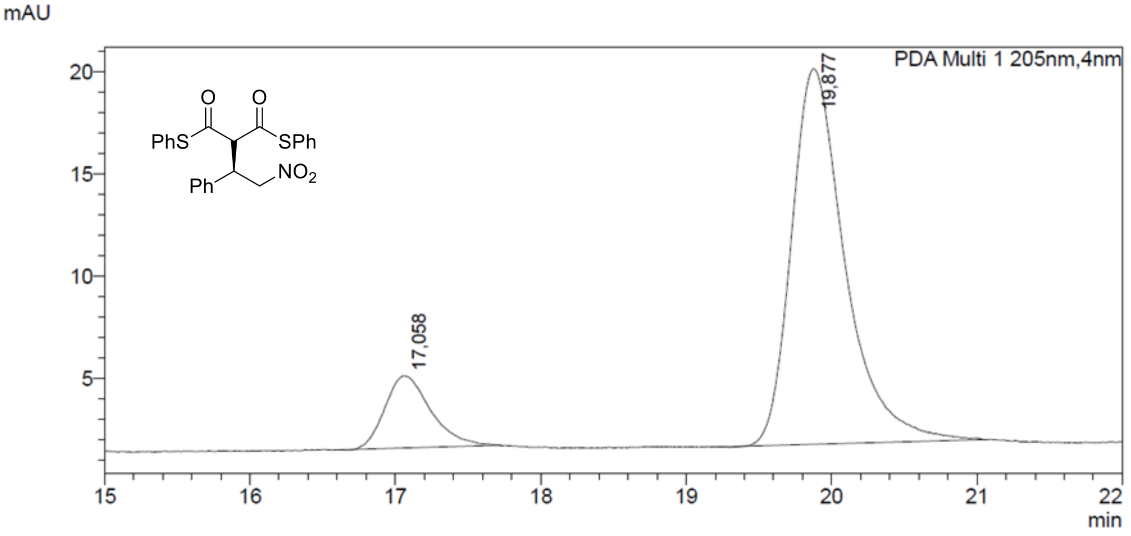

<Peak Table>

PDA Ch1 205nm

Peak#	Ret. Time	Height	Area	Area%
1	17,058	3537	77408	14,109
2	19,877	18372	471229	85,891
Total		21908	548638	100,000

Figure S28. HPLC chromatogram for *R*-**9**: sample obtained with catalyst *e*QD-**5j** in a solution at 298 K (72% ee).

<Chromatogram>

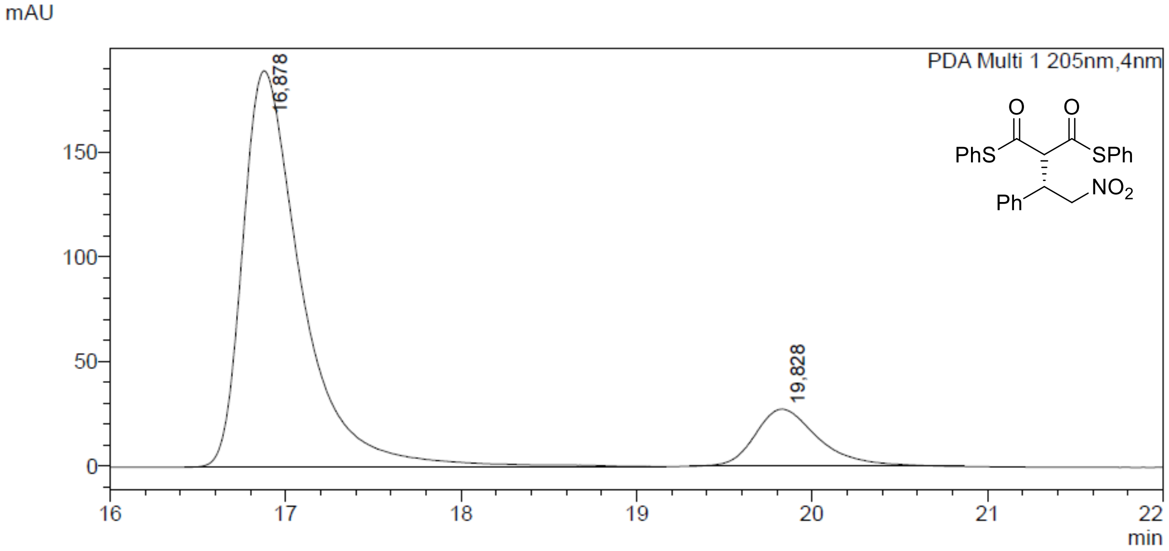

<Peak Table>

PDA Ch1 205nm

Peak#	Ret. Time	Height	Area	Area%
1	16,878	189594	4445119	86,582
2	19,828	27354	688880	13,418
Total		216948	5133999	100,000

Figure S29. HPLC chromatogram for *S*-**9**: sample obtained with catalyst *e*QN-**5a** in a planetary ball mill (73% ee).

<Chromatogram>

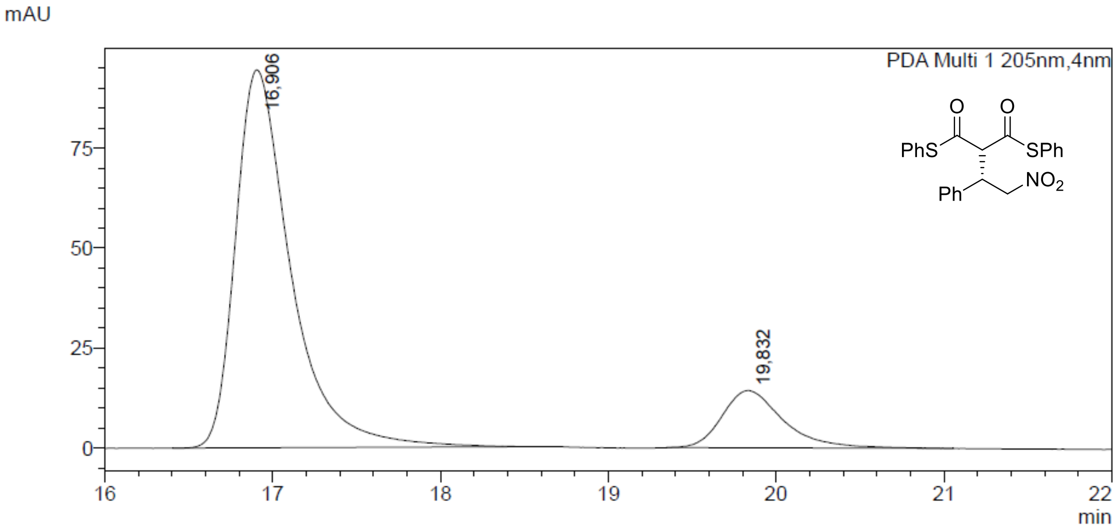

<Peak Table>

PDA Ch1 205nm

Peak#	Ret. Time	Height	Area	Area%
1	16,906	94501	2173387	85,615
2	19,832	14317	365166	14,385
Total		108818	2538553	100,000

Figure S30. HPLC chromatogram for *S*-**9**: sample obtained with catalyst *e*QN-**5b** in a planetary ball mill (71% ee).

<Chromatogram>

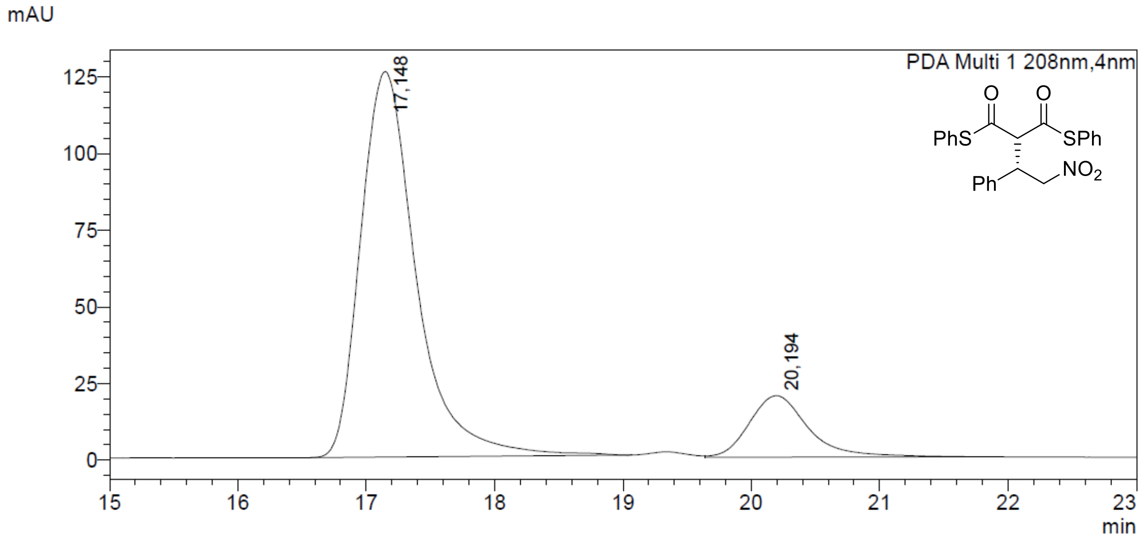

<Peak Table>

PDA Ch1 208nm

Peak#	Ret. Time	Area	Height	Area%
1	17,148	3787395	125716	85,489
2	20,194	642853	20040	14,511
Total		4430248	145756	100,000

Figure S31. HPLC chromatogram for *S*-**9**: sample obtained with catalyst *e*QN-**5c** in a planetary ball mill (71% ee).

<Chromatogram>

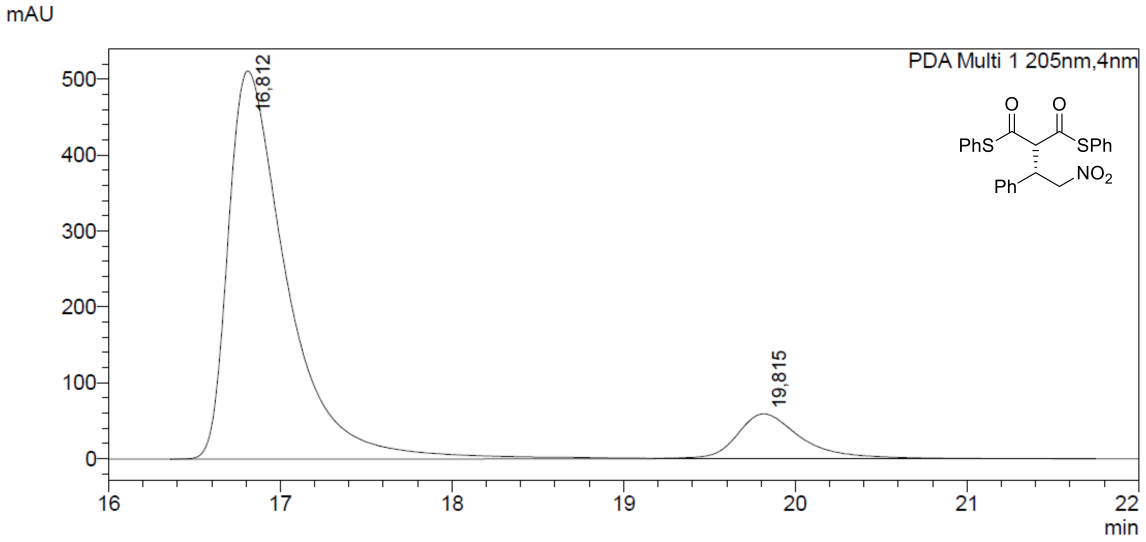

<Peak Table>

PDA Ch1 205nm

Peak#	Ret. Time	Height	Height%	Area	Area%
1	16,812	511710	89,592	12208380	88,469
2	19,815	59444	10,408	1591303	11,531
Total		571154	100,000	13799683	100,000

Figure S32. HPLC chromatogram for *S*-**9**: sample obtained with catalyst *e*QN-**5d** in a planetary ball mill (77% ee).

<Chromatogram>

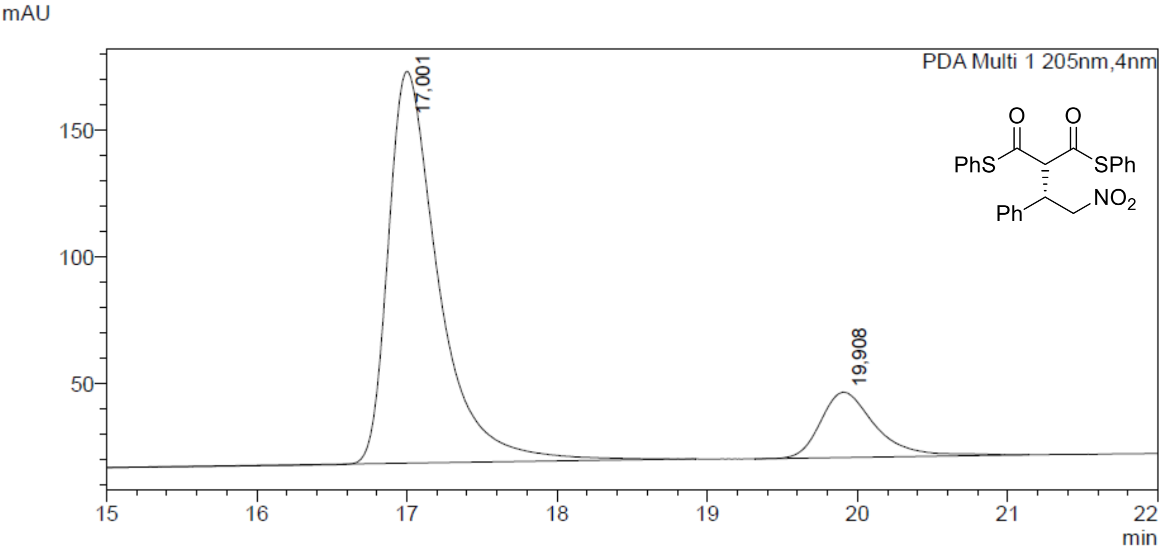

<Peak Table>

PDA Ch1 205nm

Peak#	Ret. Time	Height	Area	Area%
1	17,001	154644	3589373	84,520
2	19,908	25780	657379	15,480
Total		180424	4246751	100,000

Figure S33. HPLC chromatogram for *S*-**9**: sample obtained with catalyst *e*QN-**5e** in a planetary ball mill (69% ee).

<Chromatogram>

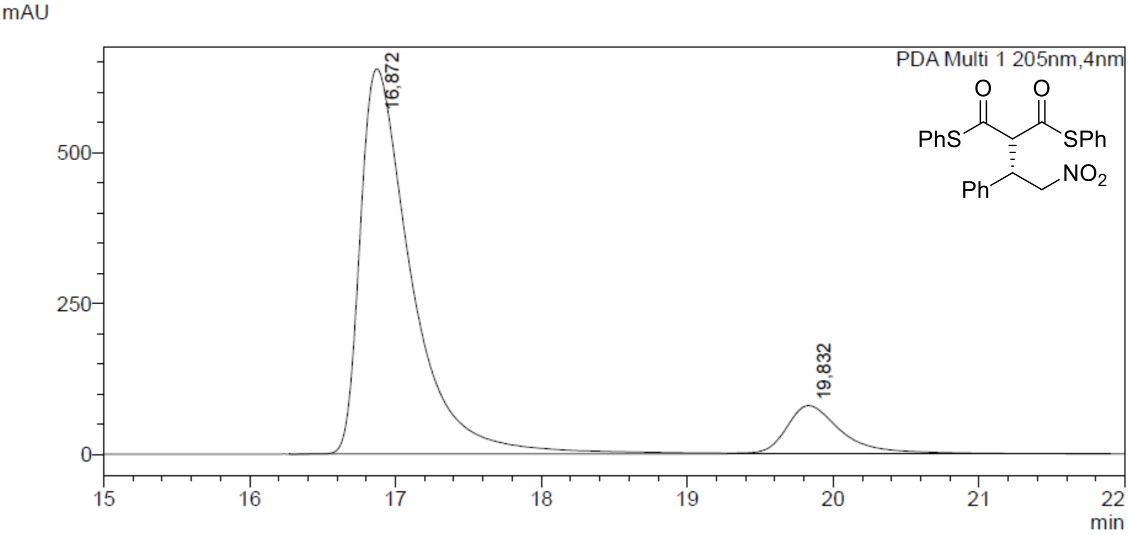

<Peak Table>

PDA Ch1 205nm

Peak#	Ret. Time	Height	Area	Area%
1	16,872	637452	15566419	87,961
2	19,832	79798	2130531	12,039
Total		717251	17696949	100,000

Figure S34. HPLC chromatogram for *S*-**9**: sample obtained with catalyst *e*CD-**5f** in a planetary ball mill (76% ee).

<Chromatogram>

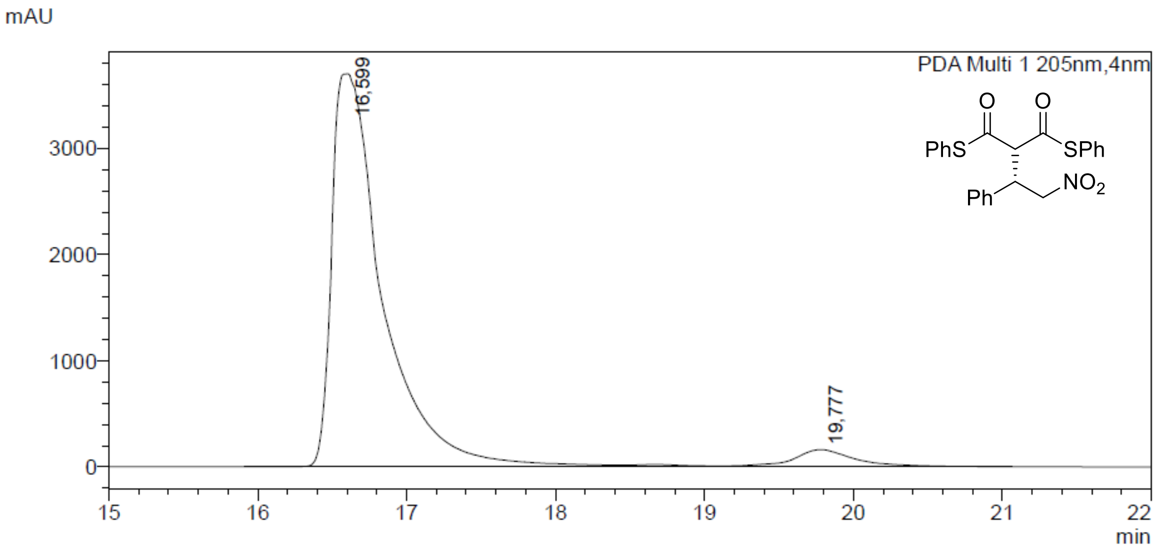

<Peak Table>

PDA Ch1 205nm

Peak#	Ret. Time	Height	Height%	Area	Area%
1	16,599	3700182	95,838	88370769	94,928
2	19,777	160698	4,162	4721399	5,072
Total		3860880	100,000	93092167	100,000

Figure S35. HPLC chromatogram for *S*-**9**: sample obtained with catalyst *e*DHQN-**5g** in a planetary ball mill (90% ee).

<Chromatogram>

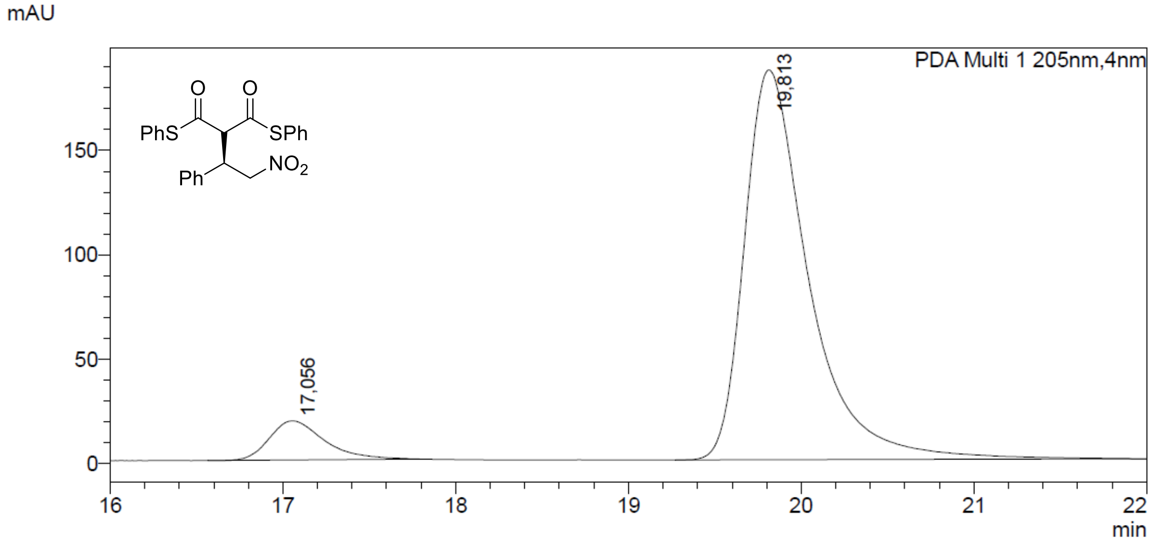

<Peak Table>

PDA Ch1 205nm

Peak#	Ret. Time	Height	Area	Area%
1	17,056	18711	413528	7,865
2	19,813	186635	4844052	92,135
Total		205346	5257580	100,000

Figure S36. HPLC chromatogram for *R*-**9**: sample obtained with catalyst *e*QD-**5h** in a planetary ball mill (84% ee).

<Chromatogram>

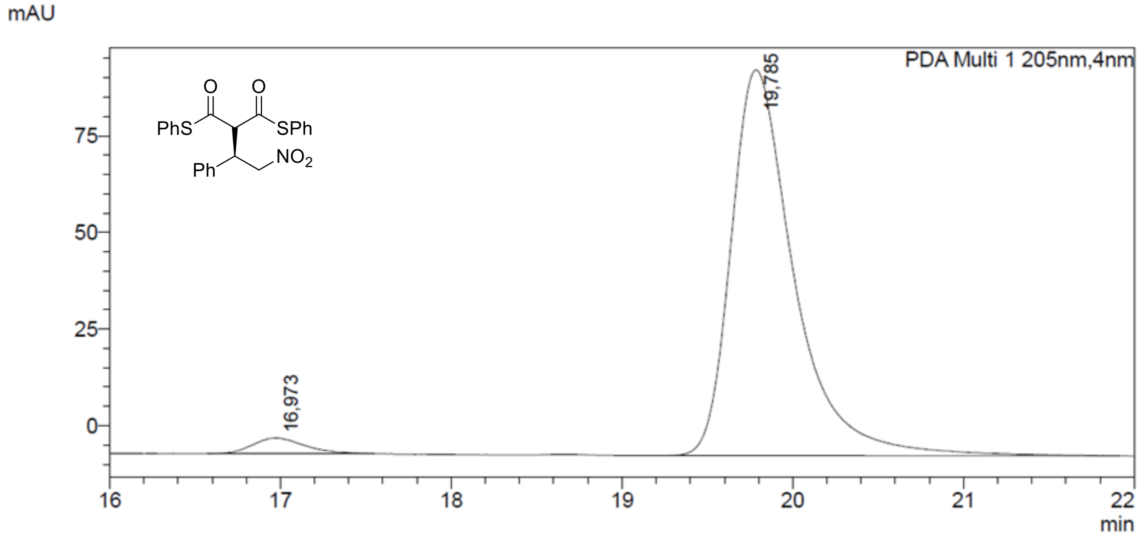

<Peak Table>

PDA Ch1 205nm

Peak#	Ret. Time	Height	Area	Area%
1	16,973	4056	87109	3,282
2	19,785	99873	2566932	96,718
Total		103929	2654041	100,000

Figure S37. HPLC chromatogram for *R*-**9**: sample obtained with catalyst *e*QD-**5i** in a planetary ball mill (93% ee).

<Chromatogram>

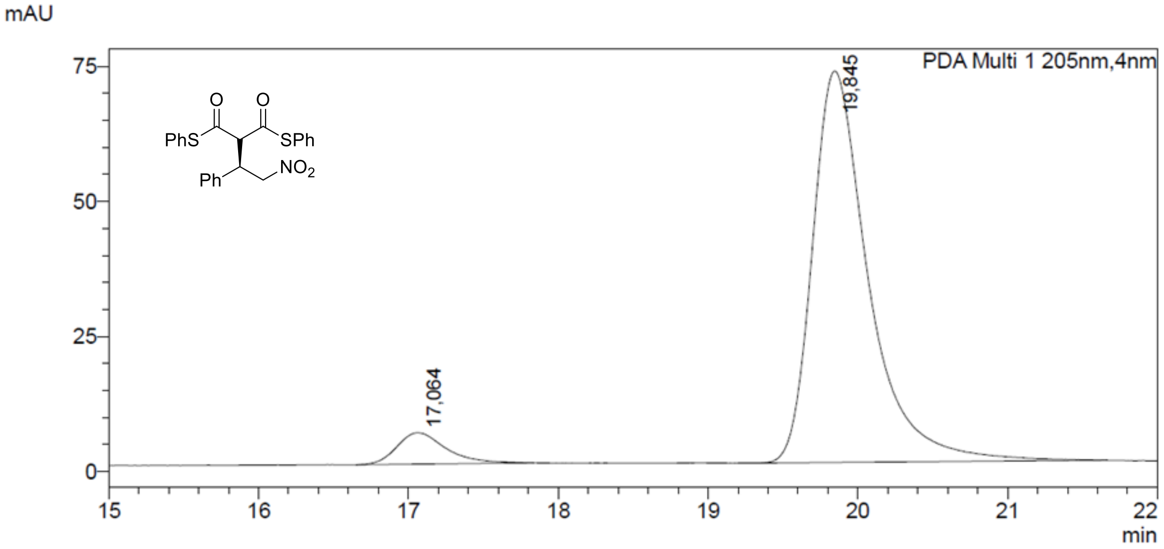

<Peak Table>

PDA Ch1 205nm

Peak#	Ret. Time	Height	Area	Area%
1	17,064	5818	132206	6,546
2	19,845	72434	1887444	93,454
Total		78253	2019650	100,000

Figure S38. HPLC chromatogram for *R*-**9**: sample obtained with catalyst *e*QD-**5j** in a planetary ball mill (87% ee).

<Chromatogram>

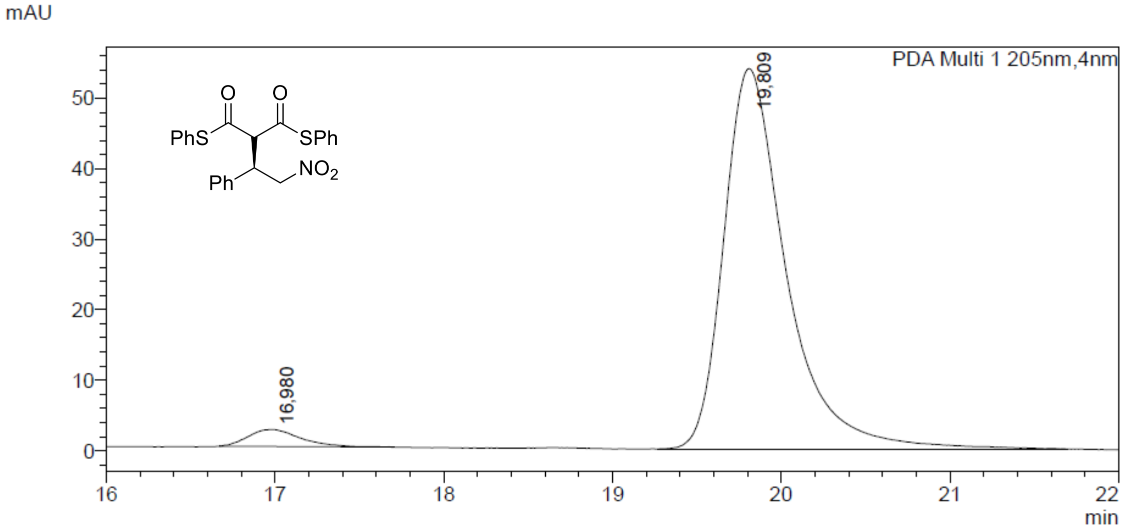

<Peak Table>

PDA Ch1 205nm

Peak#	Ret. Time	Height	Area	Area%
1	16,980	2425	51669	3,578
2	19,809	53946	1392537	96,422
Total		56370	1444206	100,000

Figure S39. HPLC chromatogram for *R*-**9**: sample obtained with catalyst *e*DHQD-**5k** in a planetary ball mill (93% ee).

<Chromatogram>

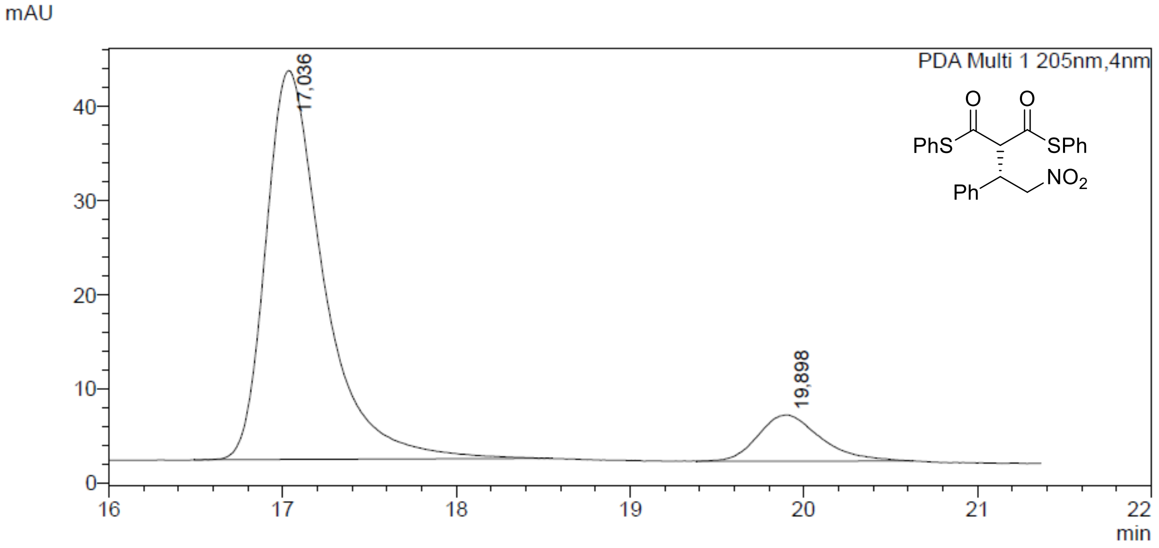

<Peak Table>

PDA Ch1 205nm

Peak#	Ret. Time	Height	Area	Area%
1	17,036	41302	960308	88,670
2	19,898	4904	122704	11,330
Total		46206	1083012	100,000

Figure S40. HPLC chromatogram for *S*-**9**: sample obtained with catalyst *e*QN-**6a** in a planetary ball mill (77% ee).

<Chromatogram>

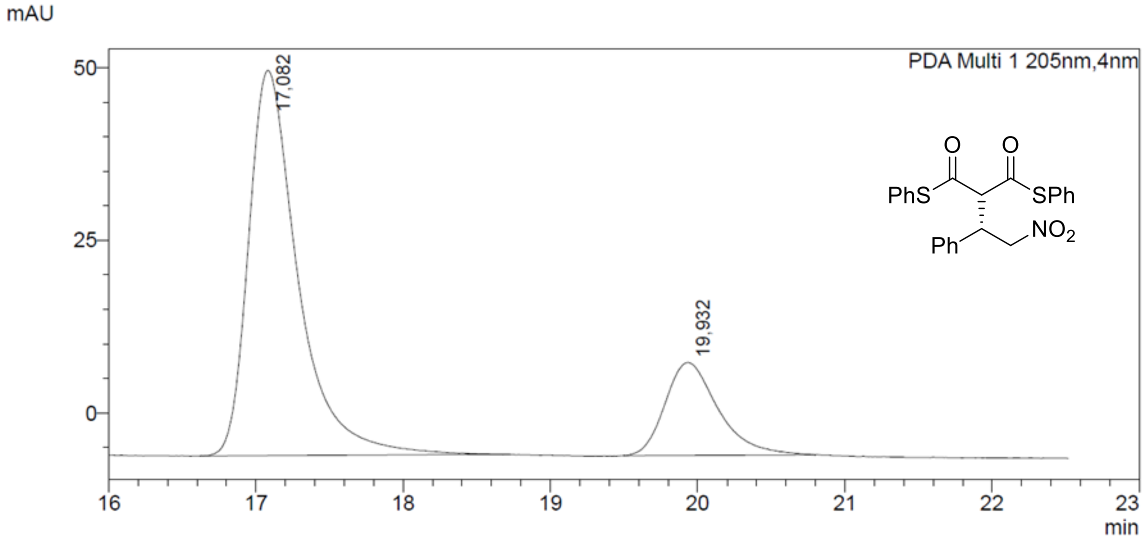

<Peak Table>

PDA Ch1 205nm

Peak#	Ret. Time	Height	Area	Area%
1	17,082	55746	1304014	79,476
2	19,932	13427	336744	20,524
Total		69173	1640759	100,000

Figure S41. HPLC chromatogram for *S*-**9**: sample obtained with catalyst *e*QN-**6b** in a planetary ball mill (59% ee).

S4.2. Asymmetric Michael hemiacetalization reaction of benzylidenepyruvate and dimedone

(Chiralcel IA-3, n-hexane/2-propanol, 7:3, flow rate 1mL/min, λ=254 nm)

<Chromatogram>

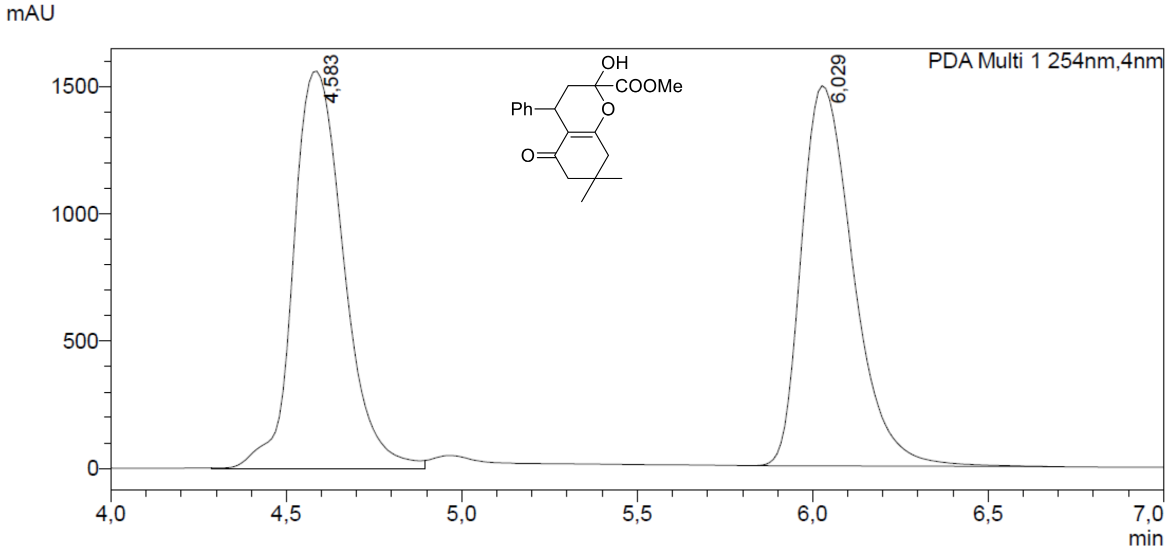

<Peak Table>

PDA Ch1 254nm

Peak#	Ret. Time	Height	Height%	Area	Area%
1	4,583	1559716	51,085	15576963	50,373
2	6,029	1493435	48,915	15346567	49,627
Total		3053151	100,000	30923529	100,000

Figure S42. HPLC chromatogram for racemic 4-methyl-2-hydroxy-7,7-dimethyl-5-oxo-4-phenyl-3,4,5,6,7,8-hexahydro-2*H*-chromene-2-carboxylate **12.**

<Chromatogram>

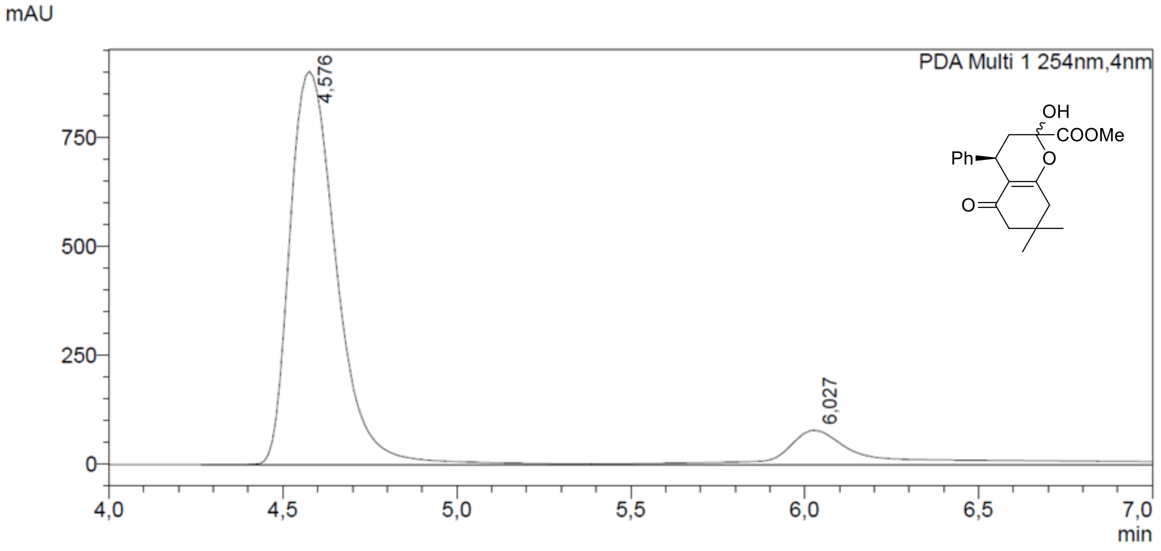

<Peak Table>

PDA Ch1 254nm

Peak#	Ret. Time	Height	Height%	Area	Area%
1	4,576	902212	92,039	8408378	83,272
2	6,027	78042	7,961	1689103	16,728
Total		980254	100,000	10097481	100,000

Figure S43. HPLC chromatogram for *R*-**12**: sample obtained with catalyst *e*QN-**5a** in a solution at 298 K (67% ee).

<Chromatogram>

mAU

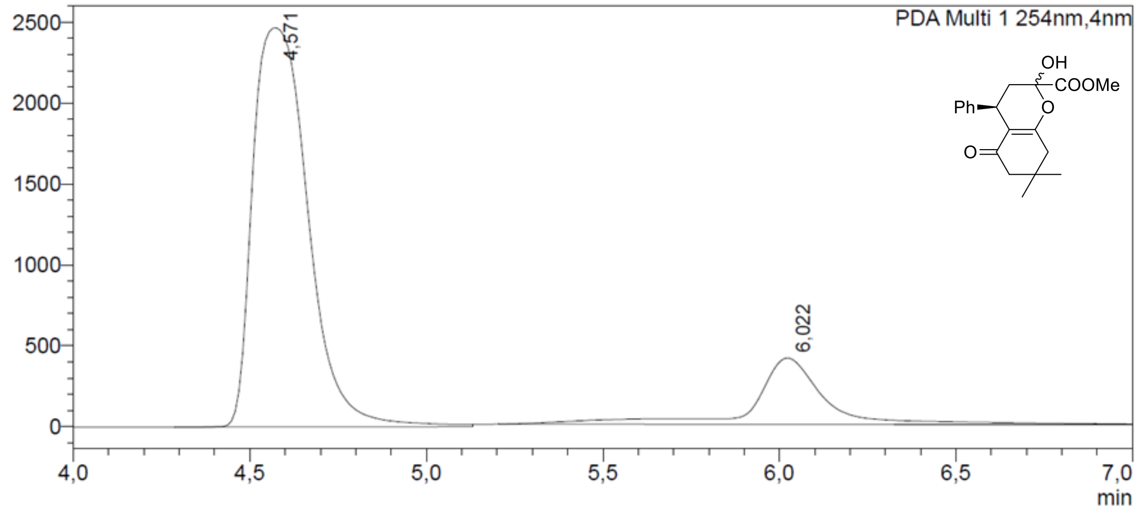

<Peak Table>

PDA Ch1 254nm

Peak#	Ret. Time	Height	Height%	Area	Area%
1	4,571	2465931	85,755	26895481	81,877
2	6,022	409621	14,245	5953244	18,123
Total		2875552	100,000	32848725	100,000

Figure S44. HPLC chromatogram for *R*-**12**: sample obtained with catalyst *e*QN-**5b** in a solution at 298 K (64% ee).

<Chromatogram>

mAU

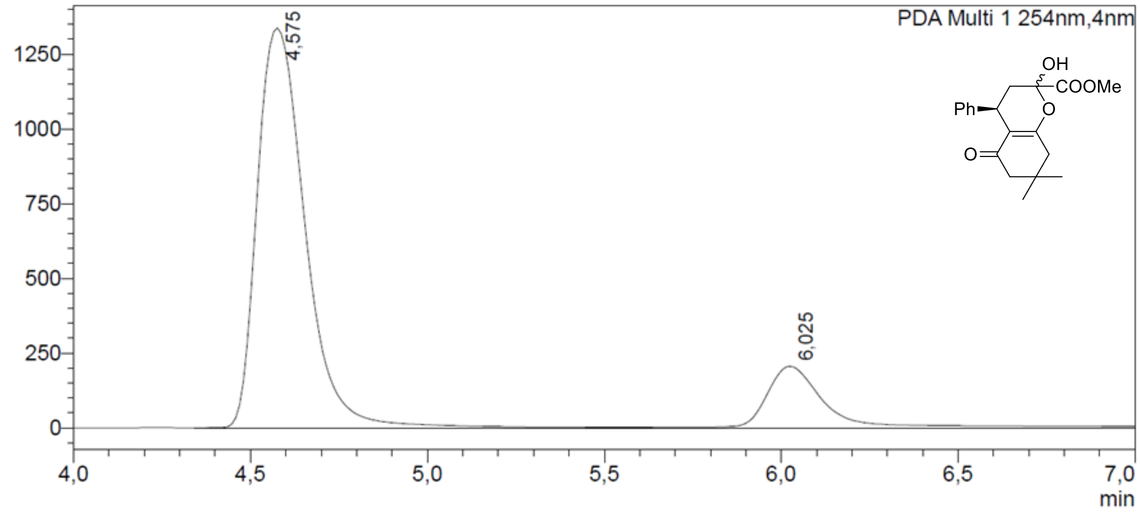

<Peak Table>

PDA Ch1 254nm

Peak#	Ret. Time	Height	Height%	Area	Area%
1	4,575	1337383	86,659	12598230	82,444
2	6,025	205891	13,341	2682791	17,556
Total		1543273	100,000	15281022	100,000

Figure S45. HPLC chromatogram for *R*-**12**: sample obtained with catalyst *e*QN-**5c** in a solution at 298 K (65% ee).

<Chromatogram>

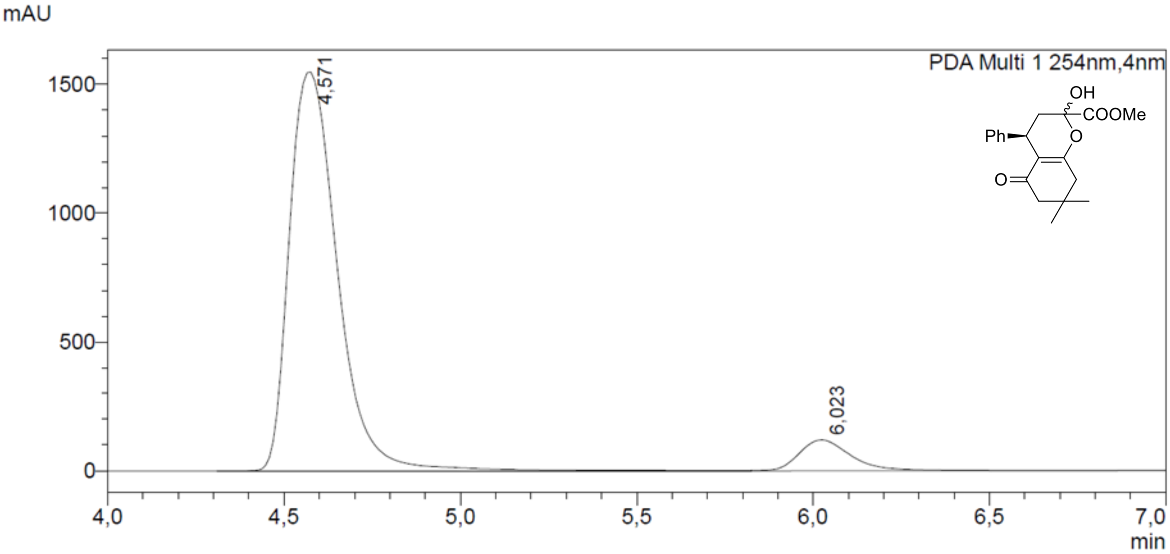

<Peak Table>

PDA Ch1 254nm

Peak#	Ret. Time	Height	Height%	Area	Area%
1	4,571	1547690	92,827	14697718	92,200
2	6,023	119596	7,173	1243343	7,800
Total		1667287	100,000	15941061	100,000

Figure S46. HPLC chromatogram for *R*-**12**: sample obtained with catalyst *e*QN-**5d** in a solution at 298 K (84% ee).

<Chromatogram>

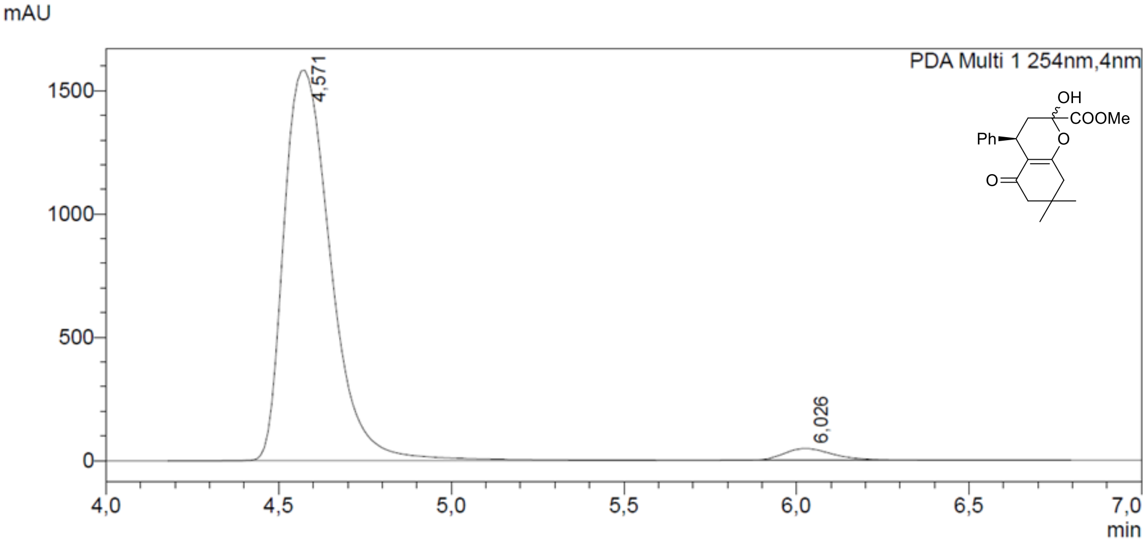

<Peak Table>

PDA Ch1 254nm

Peak#	Ret. Time	Height	Height%	Area	Area%
1	4,571	1582090	97,053	14985419	96,856
2	6,026	48037	2,947	486491	3,144
Total		1630128	100,000	15471910	100,000

Figure S47. HPLC chromatogram for *R*-**12**: sample obtained with catalyst *e*QN-**5e** in a solution at 298 K (94% ee).

<Chromatogram>

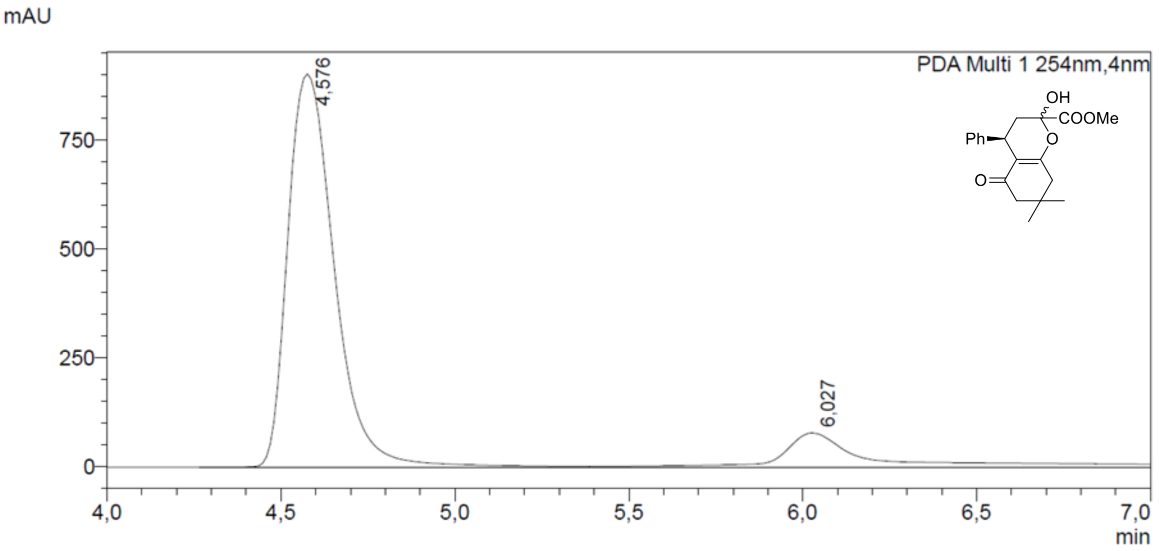

<Peak Table>

PDA Ch1 254nm

Peak#	Ret. Time	Height	Height%	Area	Area%
1	4,576	902212	92,039	8408378	83,272
2	6,027	78042	7,961	1689103	16,728
Total		980254	100,000	10097481	100,000

Figure S48. HPLC chromatogram for *R*-**12**: sample obtained with catalyst *e*CD-**5f** in a solution at 298 K (66% ee).

<Chromatogram>

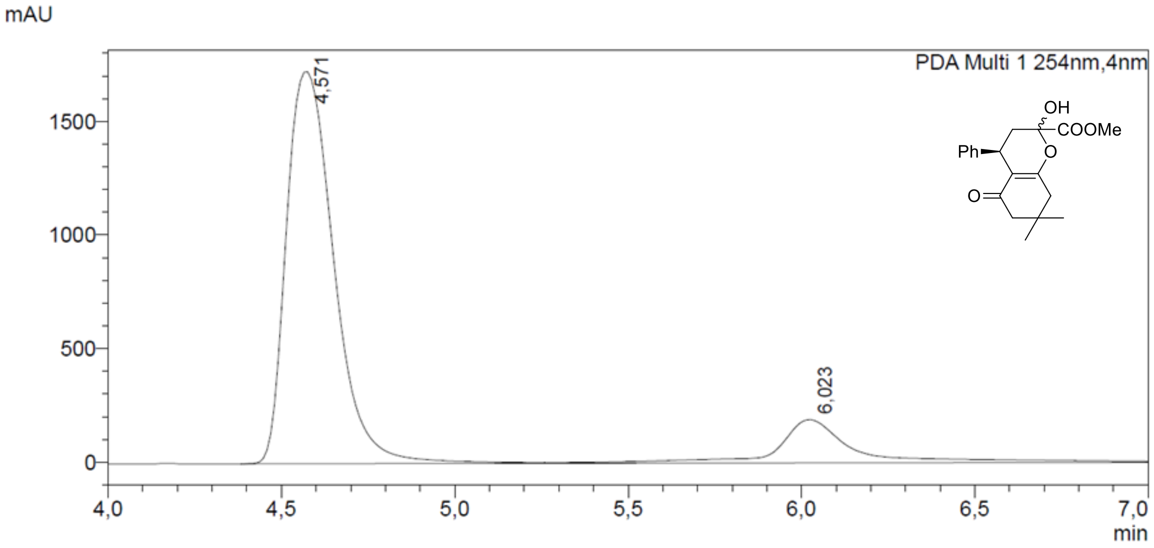

<Peak Table>

PDA Ch1 254nm

Peak#	Ret. Time	Height	Height%	Area	Area%
1	4,571	1724053	90,063	16464213	84,351
2	6,023	190219	9,937	3054525	15,649
Total		1914272	100,000	19518738	100,000

Figure S49. HPLC chromatogram for *R*-**12**: sample obtained with catalyst *e*DHQN-**5g** in a solution at 298 K (69% ee).

<Chromatogram>

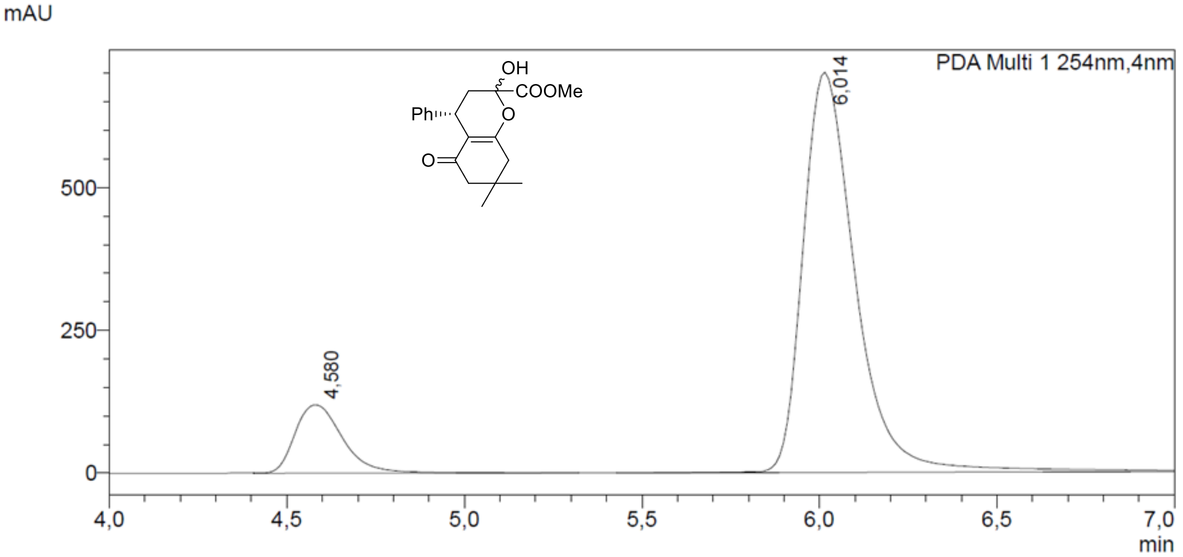

<Peak Table>

PDA Ch1 254nm

Peak#	Ret. Time	Height	Height%	Area	Area%
1	4,580	119665	14,577	1120225	13,329
2	6,014	701251	85,423	7283971	86,671
Total		820917	100,000	8404196	100,000

Figure S50. HPLC chromatogram for *S*-**12**: sample obtained with catalyst *e*QD-**5h** in a solution at 298 K (73% ee).

<Chromatogram>

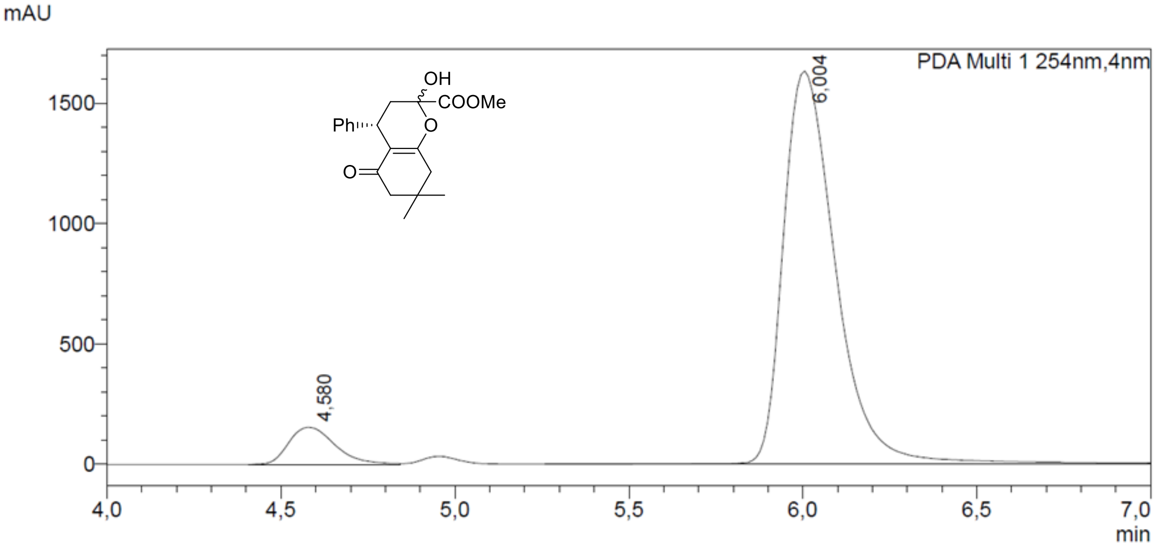

<Peak Table>

PDA Ch1 254nm

Peak#	Ret. Time	Area	Area%
1	4,580	1424606	7,567
2	6,004	17401201	92,433
Total		18825806	100,000

Figure S51. HPLC chromatogram for *S*-**12**: sample obtained with catalyst *e*QD-**5i** in a solution at 298 K (85% ee).

<Chromatogram>

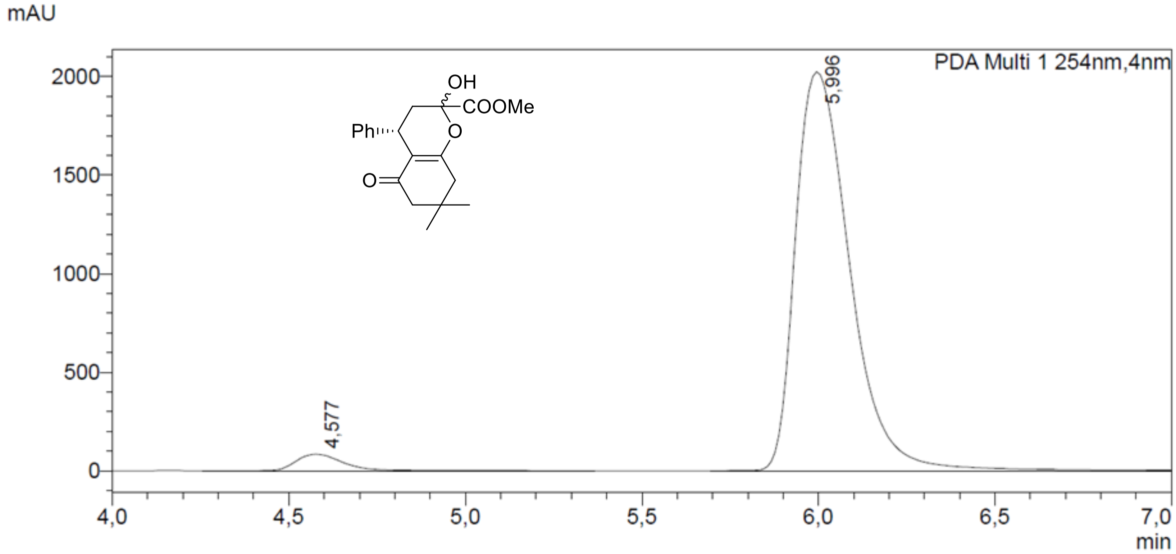

<Peak Table>

PDA Ch1 254nm

Peak#	Ret. Time	Height	Height%	Area	Area%
1	4,577	83457	3,958	780476	3,454
2	5,996	2025155	96,042	21815039	96,546
Total		2108613	100,000	22595515	100,000

Figure S52. HPLC chromatogram for *S*-**12**: sample obtained with catalyst *e*QD-**5j** in a solution at 298 K (93% ee).

<Chromatogram>

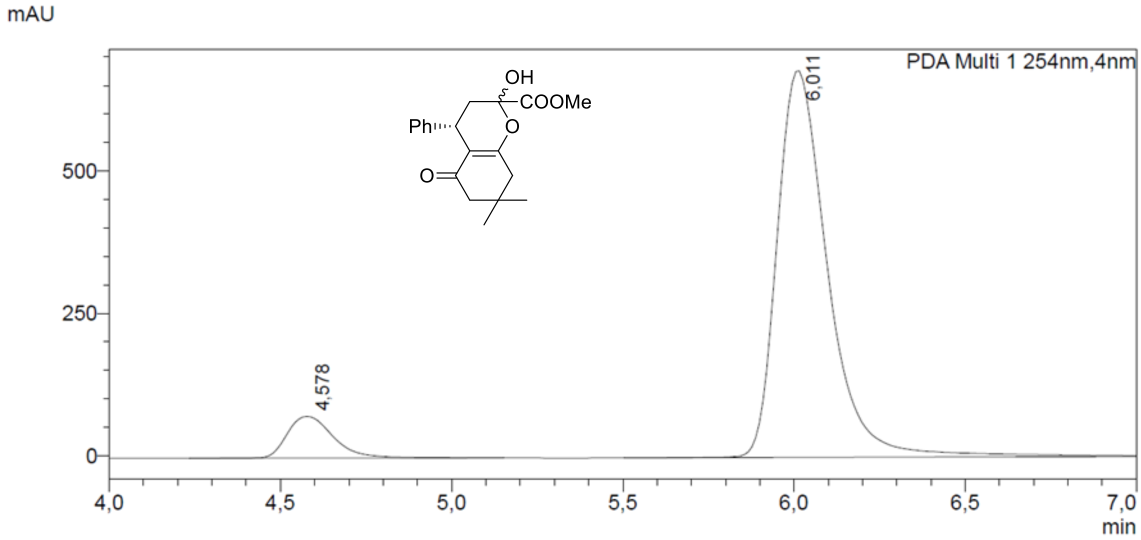

<Peak Table>

PDA Ch1 254nm

Peak#	Ret. Time	Height	Height%	Area	Area%
1	4,578	73209	9,738	684076	8,888
2	6,011	678538	90,262	7012609	91,112
Total		751746	100,000	7696686	100,000

Figure S53. HPLC chromatogram for *S*-**12**: sample obtained with catalyst *e*DHQD-**5k** in a solution at 298 K (82% ee).

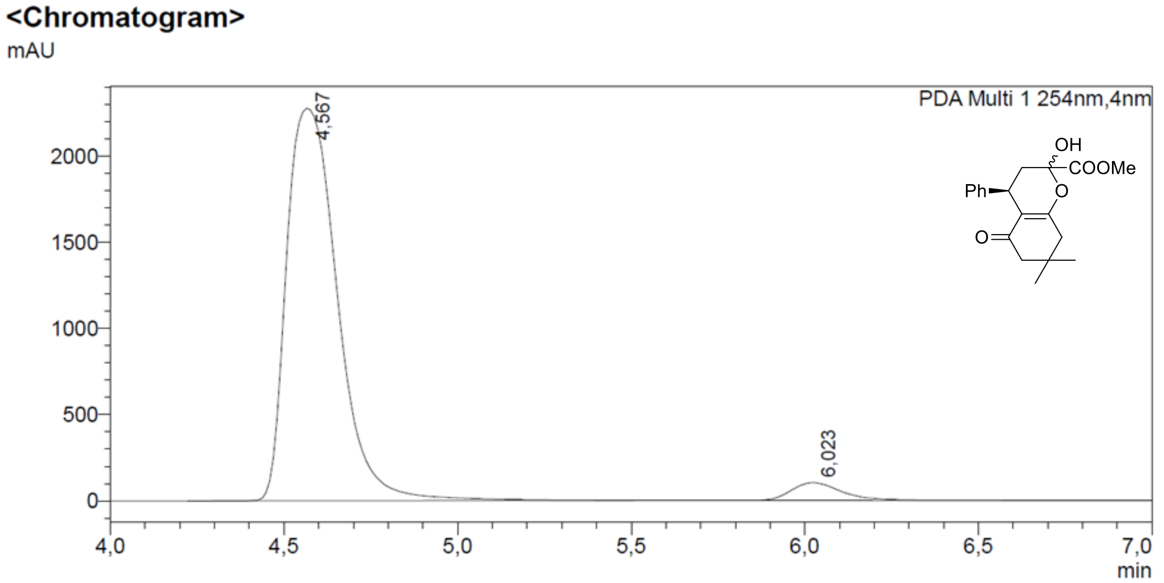

<Peak Table>

PDA Ch1 254nm

Peak#	Ret. Time	Height	Height%	Area	Area%
1	4,567	2277554	95,652	23245479	95,345
2	6,023	103522	4,348	1135007	4,655
Total		2381075	100,000	24380486	100,000

Figure S54. HPLC chromatogram for *R*-**12**: sample obtained with catalyst *e*QN-**6a** in a solution at 298 K (91% ee).

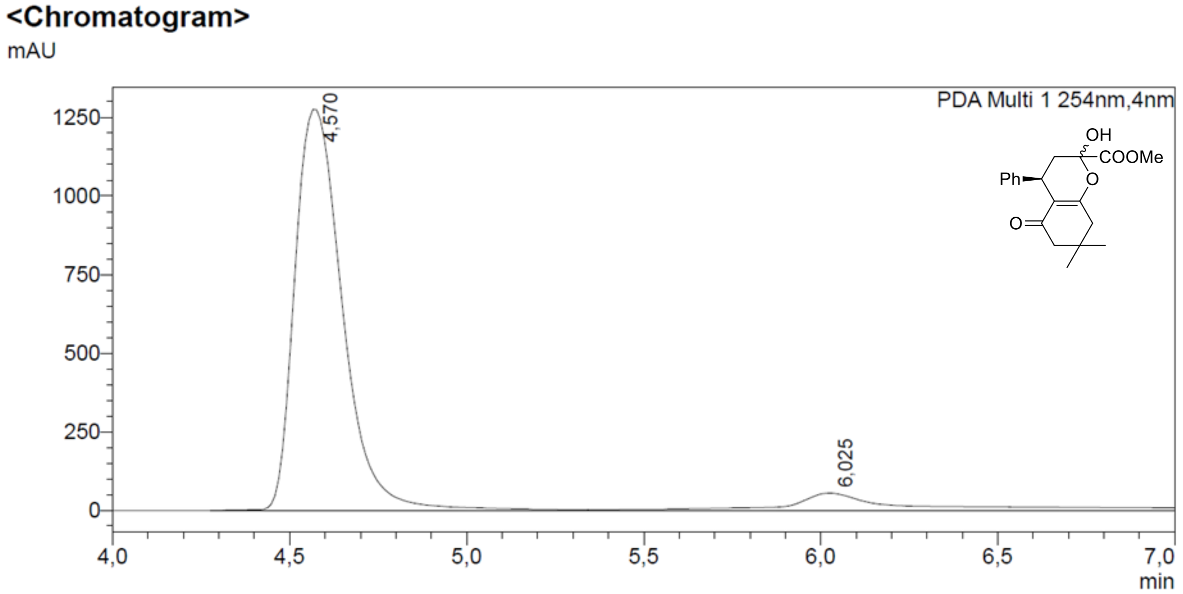

<Peak Table>

PDA Ch1 254nm

Peak#	Ret. Time	Height	Height%	Area	Area%
1	4,570	1274846	95,895	11964344	87,764
2	6,025	54568	4,105	1668130	12,236
Total		1329414	100,000	13632473	100,000

Figure S55. HPLC chromatogram for *R*-**12**: sample obtained with catalyst *e*QN-**6b** in a solution at 298 K (76% ee).

<Chromatogram>
mAU

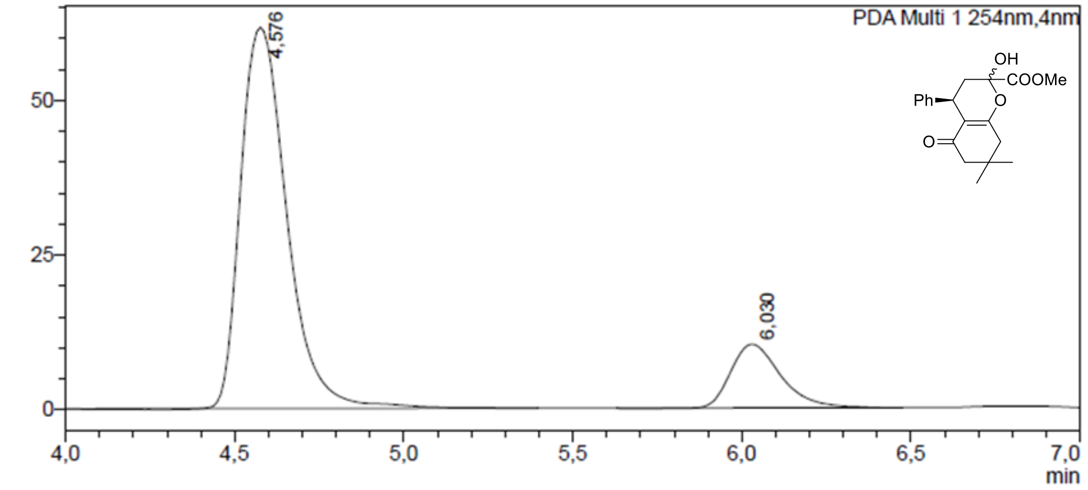

<Peak Table>

PDA Ch1 254nm

Peak#	Ret. Time	Height	Height%	Area	Area%
1	4,576	61671	85,698	586220	84,703
2	6,030	10292	14,302	105870	15,297
Total		71963	100,000	692091	100,000

Figure S56. HPLC chromatogram for *R*-**12**: sample obtained with catalyst *e*QN-**5c** in a solution at 273 K (69% ee).

<Chromatogram>
mAU

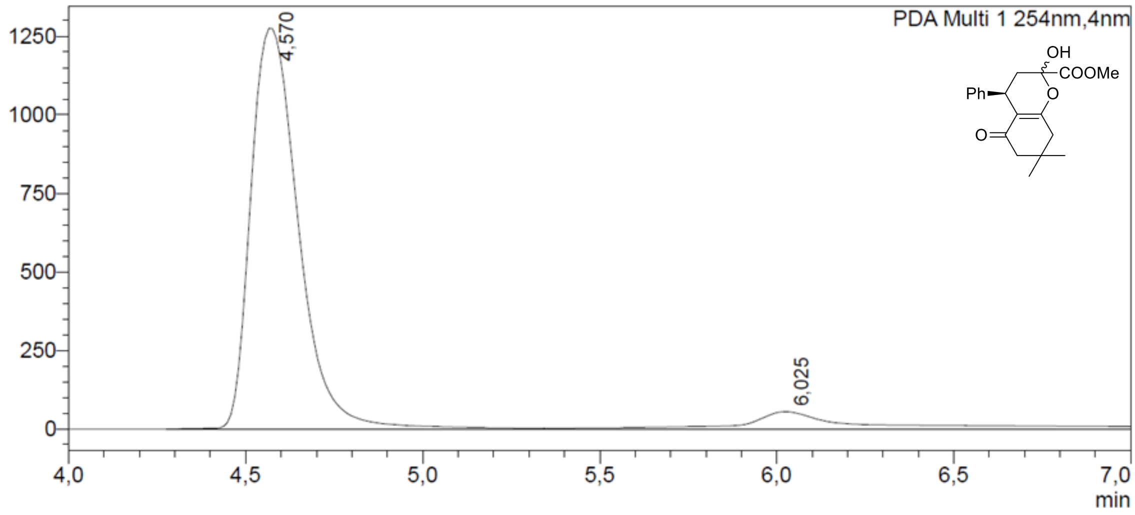

<Peak Table>

PDA Ch1 254nm

Peak#	Ret. Time	Height	Height%	Area	Area%
1	4,570	1274846	95,895	11964344	87,764
2	6,025	54568	4,105	1668130	12,236
Total		1329414	100,000	13632473	100,000

Figure S57. HPLC chromatogram for *R*-**12**: sample obtained with catalyst *e*QN-**5d** in a solution at 273 K (76% ee).

<Chromatogram>

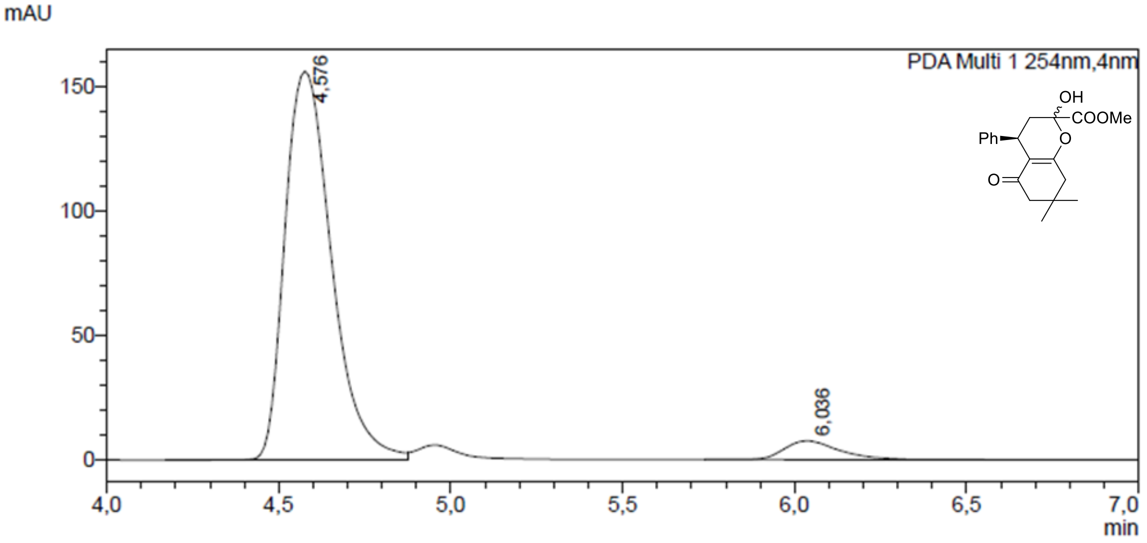

<Peak Table>

PDA Ch1 254nm

Peak#	Ret. Time	Height	Height%	Area	Area%
1	4,576	156343	95,391	1475819	94,623
2	6,036	7554	4,609	83869	5,377
Total		163897	100,000	1559688	100,000

Figure S58. HPLC chromatogram for *R*-**12**: sample obtained with catalyst *e*QN-**5e** in a solution at 273 K (89% ee).

<Chromatogram>

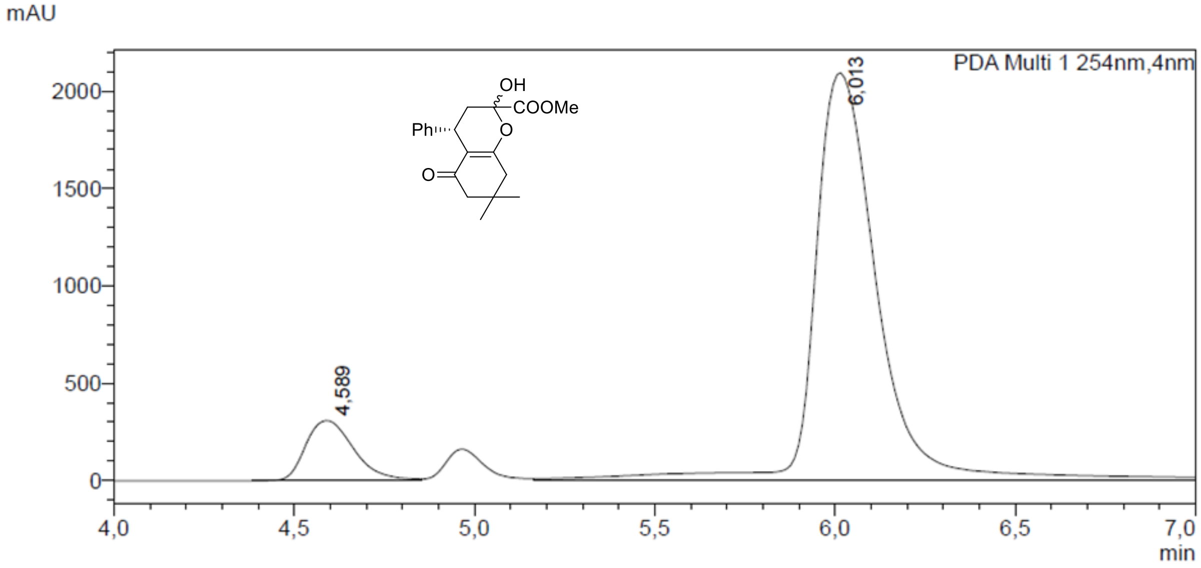

<Peak Table>

PDA Ch1 254nm

Peak#	Ret. Time	Height	Height%	Area	Area%
1	4,589	308434	12,831	2858669	9,778
2	6,013	2095348	87,169	26377099	90,222
Total		2403782	100,000	29235768	100,000

Figure S59. HPLC chromatogram for *S*-**12**: sample obtained with catalyst *e*QD-**5h** in a solution at 273 K (80% ee).

<Chromatogram>

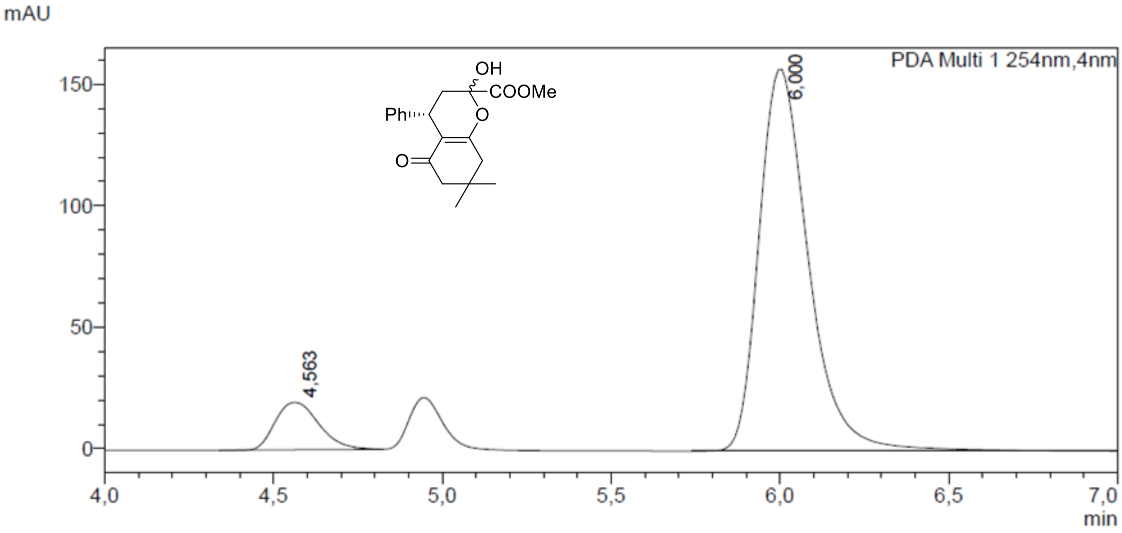

<Peak Table>

PDA Ch1 254nm

Peak#	Ret. Time	Height	Area	Area%
1	4,563	19552	175414	9,873
2	6,000	157113	1601303	90,127
Total		176665	1776716	100,000

Figure S60. HPLC chromatogram for *S*-**12**: sample obtained with catalyst *e*QD-**5i** in a solution at 273 K (80% ee).

<Chromatogram>

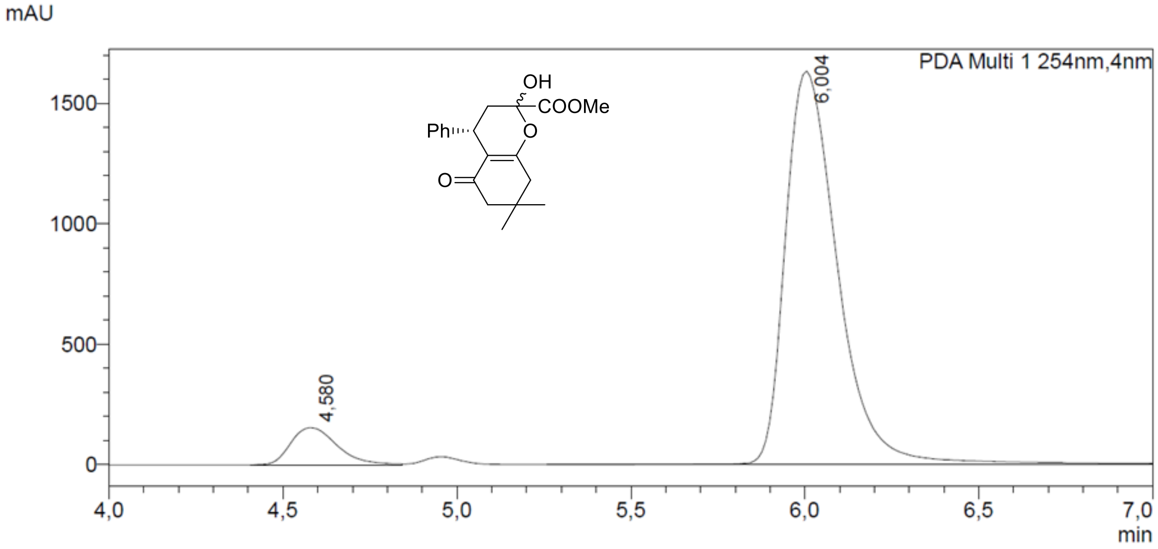

<Peak Table>

PDA Ch1 254nm

Peak#	Ret. Time	Area	Area%
1	4,580	1424606	7,567
2	6,004	17401201	92,433
Total		18825806	100,000

Figure S61. HPLC chromatogram for *S*-**12**: sample obtained with catalyst *e*QD-**5j** in a solution at 273 K (85% ee).

<Chromatogram>

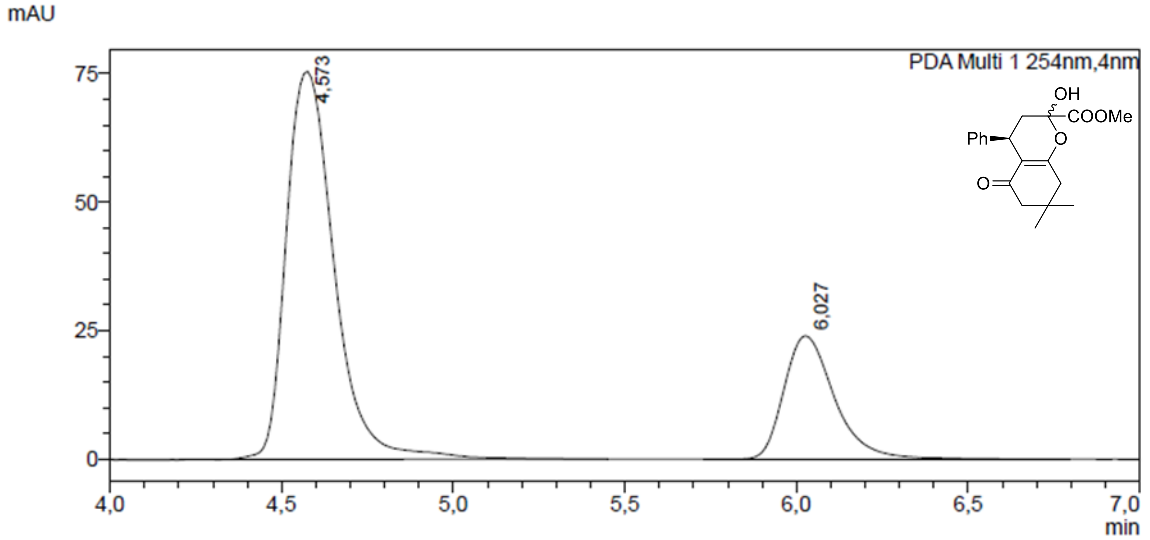

<Peak Table>

PDA Ch1 254nm

Peak#	Ret. Time	Height	Height%	Area	Area%
1	4,573	75418	75,853	735167	74,876
2	6,027	24009	24,147	246680	25,124
Total		99427	100,000	981847	100,000

Figure S62. HPLC chromatogram for *R*-**12**: sample obtained with catalyst *e*QN-**5a** in a planetary ball mill (50% ee).

<Chromatogram>

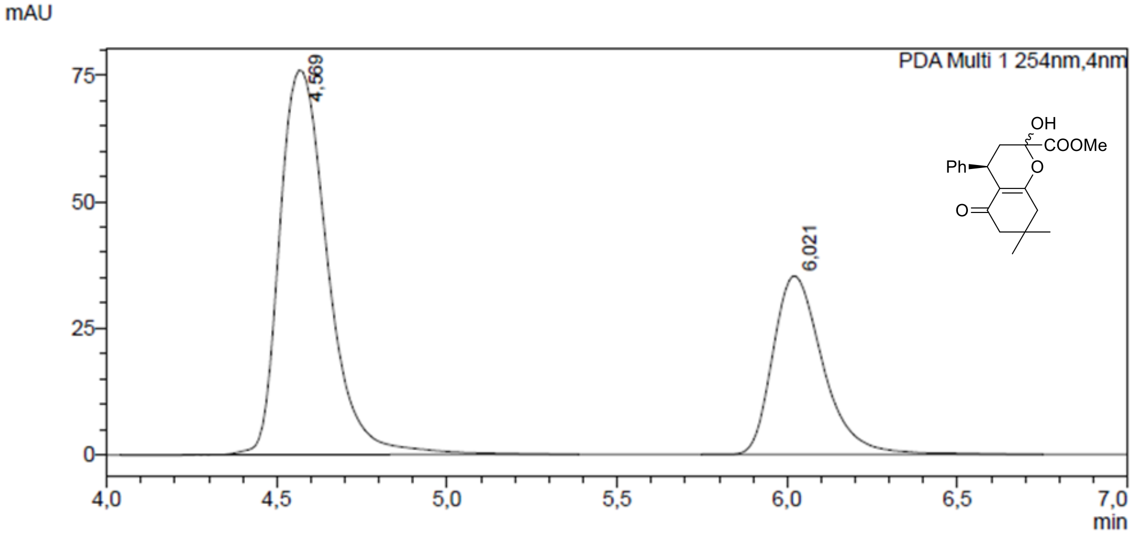

<Peak Table>

PDA Ch1 254nm

Peak#	Ret. Time	Height	Height%	Area	Area%
1	4,569	76073	68,320	746696	67,304
2	6,021	35275	31,680	362746	32,696
Total		111348	100,000	1109442	100,000

Figure S63. HPLC chromatogram for *R*-**12**: sample obtained with catalyst *e*QN-**5b** in a planetary ball mill (35% ee).

<Chromatogram>

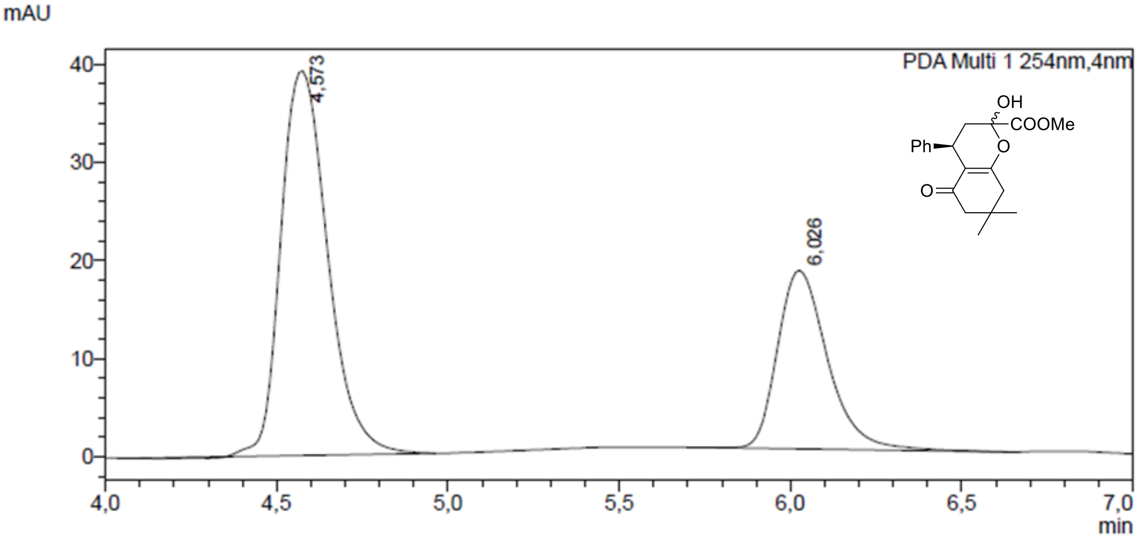

<Peak Table>

PDA Ch1 254nm

Peak#	Ret. Time	Height	Height%	Area	Area%
1	4,573	39171	68,272	372081	66,775
2	6,026	18204	31,728	185132	33,225
Total		57375	100,000	557213	100,000

Figure S64. HPLC chromatogram for *R*-**12**: sample obtained with catalyst *e*QN-**5c** in a planetary ball mill (34% ee).

<Chromatogram>

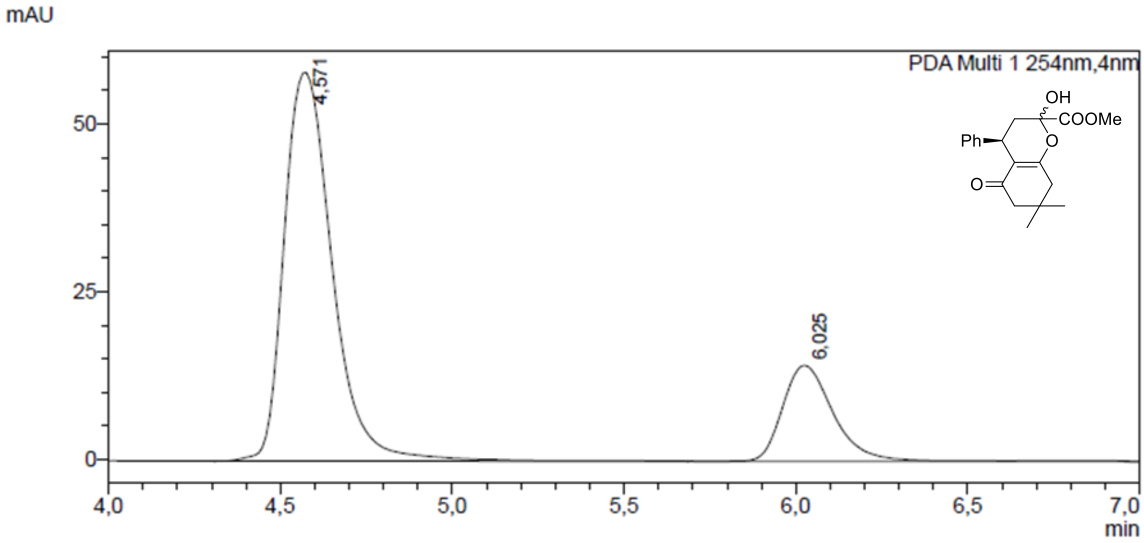

<Peak Table>

PDA Ch1 254nm

Peak#	Ret. Time	Height	Height%	Area	Area%
1	4,571	57853	80,176	565868	79,073
2	6,025	14305	19,824	149757	20,927
Total		72158	100,000	715625	100,000

Figure S65. HPLC chromatogram for *R*-**12**: sample obtained with catalyst *e*QN-**5d** in a planetary ball mill (58% ee).

<Chromatogram>

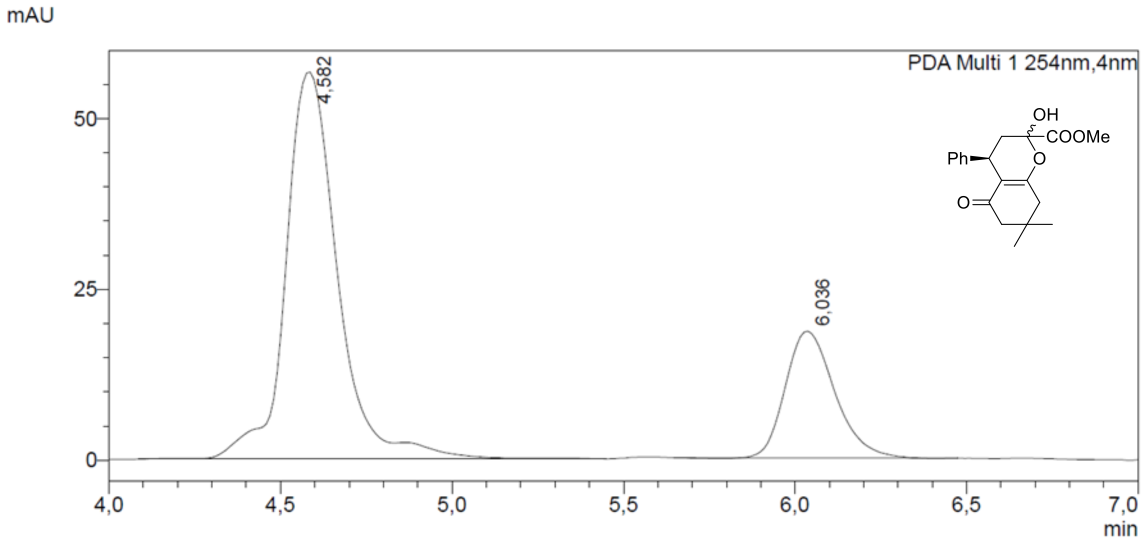

<Peak Table>

PDA Ch1 254nm

Peak#	Ret. Time	Height	Height%	Area	Area%
1	4,582	56624	75,278	599438	76,311
2	6,036	18595	24,722	186087	23,689
Total		75219	100,000	785525	100,000

Figure S66. HPLC chromatogram for *R*-**12**: sample obtained with catalyst *e*QN-**5e** in a planetary ball mill (53% ee).

<Chromatogram>

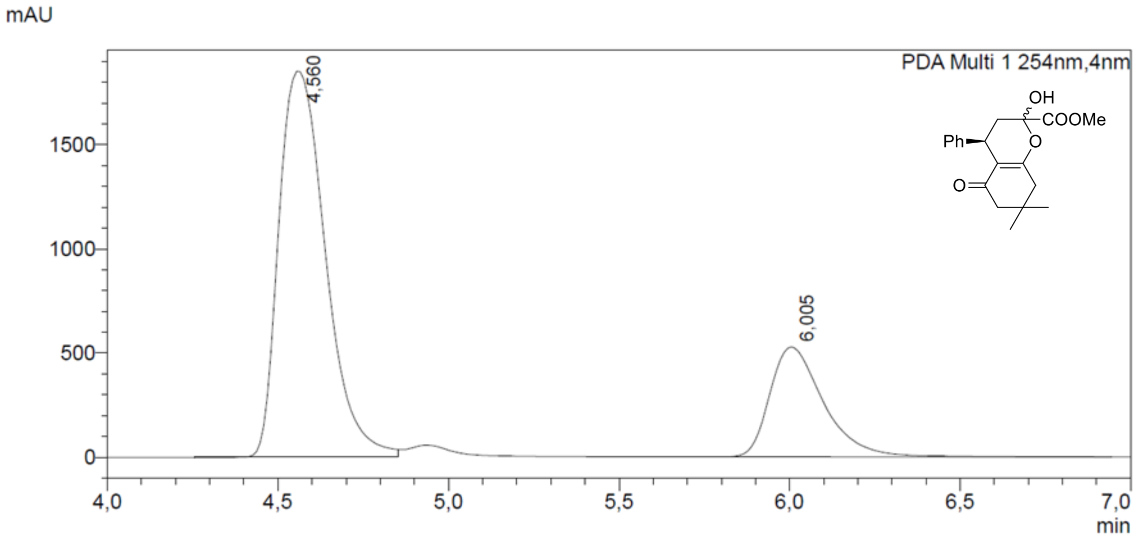

<Peak Table>

PDA Ch1 254nm

Peak#	Ret. Time	Height	Height%	Area	Area%
1	4,560	1849248	77,771	17607255	75,155
2	6,005	528551	22,229	5820726	24,845
Total		2377799	100,000	23427982	100,000

Figure S67. HPLC chromatogram for *R*-**12**: sample obtained with catalyst *e*QN-**5f** in a planetary ball mill (50% ee).

<Chromatogram>

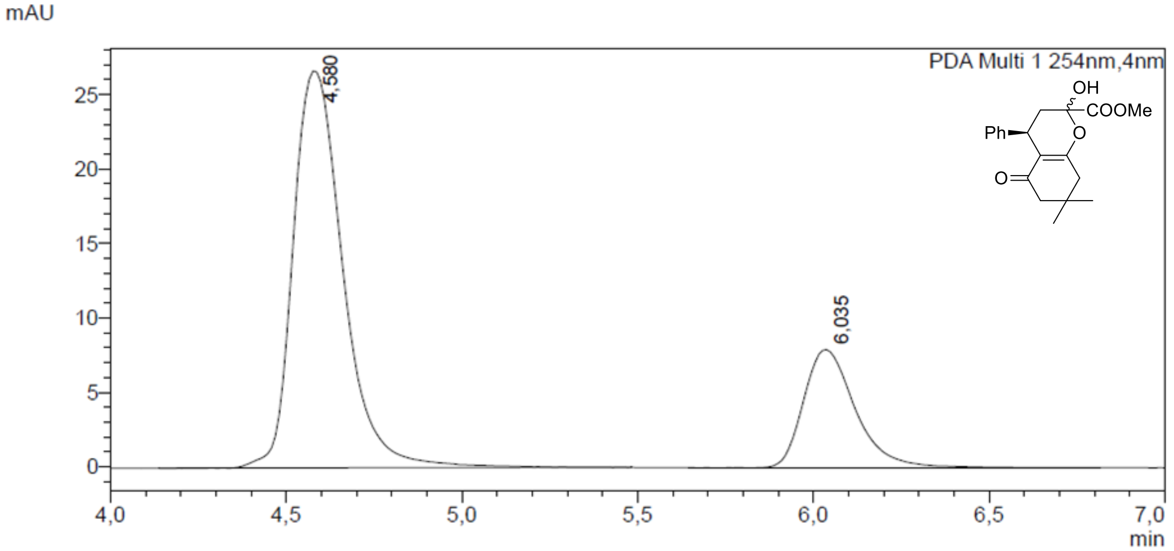

<Peak Table>

PDA Ch1 254nm

Peak#	Ret. Time	Height	Height%	Area	Area%
1	4,580	26656	77,059	263296	76,457
2	6,035	7936	22,941	81073	23,543
Total		34592	100,000	344370	100,000

Figure S68. HPLC chromatogram for *R*-**12**: sample obtained with catalyst *e*DHQN-**5g** in a planetary ball mill (53% ee).

<Chromatogram>

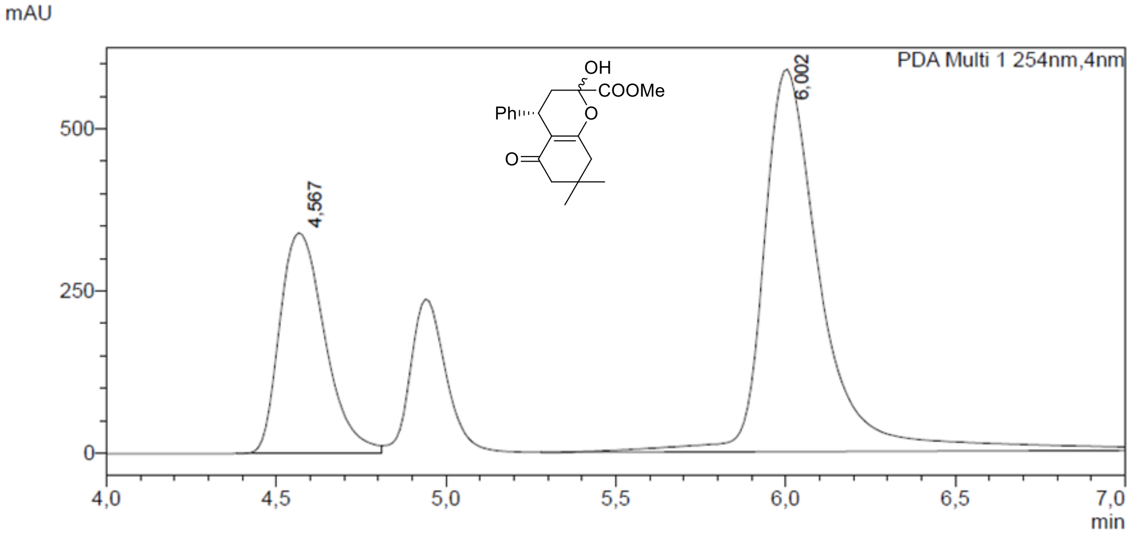

<Peak Table>

PDA Ch1 254nm

Peak#	Ret. Time	Height	Height%	Area	Area%
1	4,567	339511	36,561	3141300	30,669
2	6,002	589100	63,439	7101403	69,331
Total		928611	100,000	10242703	100,000

Figure S69. HPLC chromatogram for *S*-**12**: sample obtained with catalyst *e*QD-**5h** in a planetary ball mill (39% ee).

<Chromatogram>

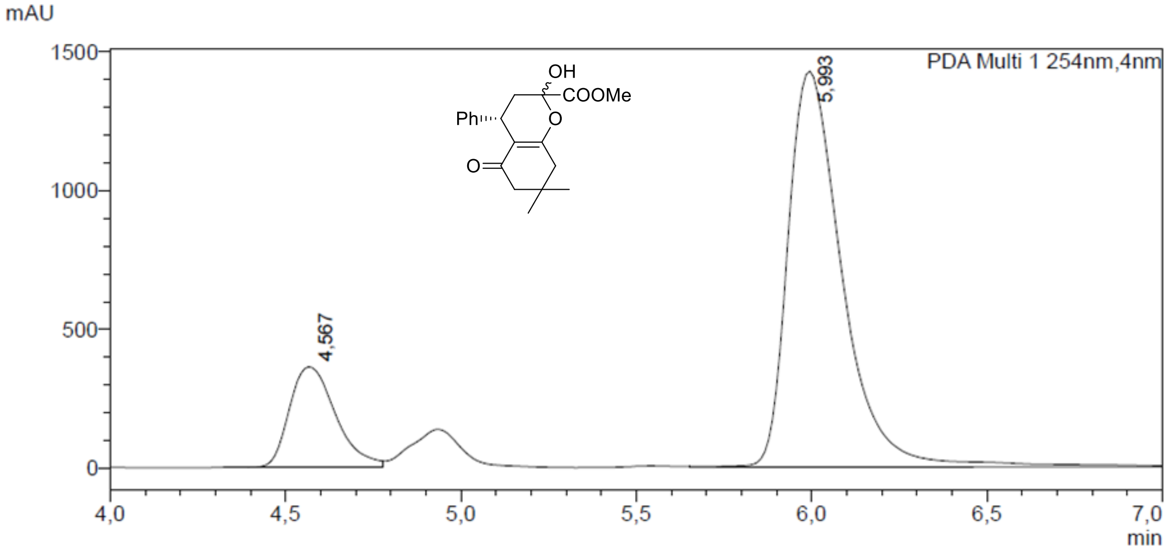

<Peak Table>

PDA Ch1 254nm

Peak#	Ret. Time	Height	Height%	Area	Area%
1	4,567	363379	20,302	3355393	17,791
2	5,993	1426529	79,698	15504737	82,209
Total		1789907	100,000	18860131	100,000

Figure S70. HPLC chromatogram for *S*-**12**: sample obtained with catalyst *e*QD-**5i** in a planetary ball mill (64% ee).

<Chromatogram>

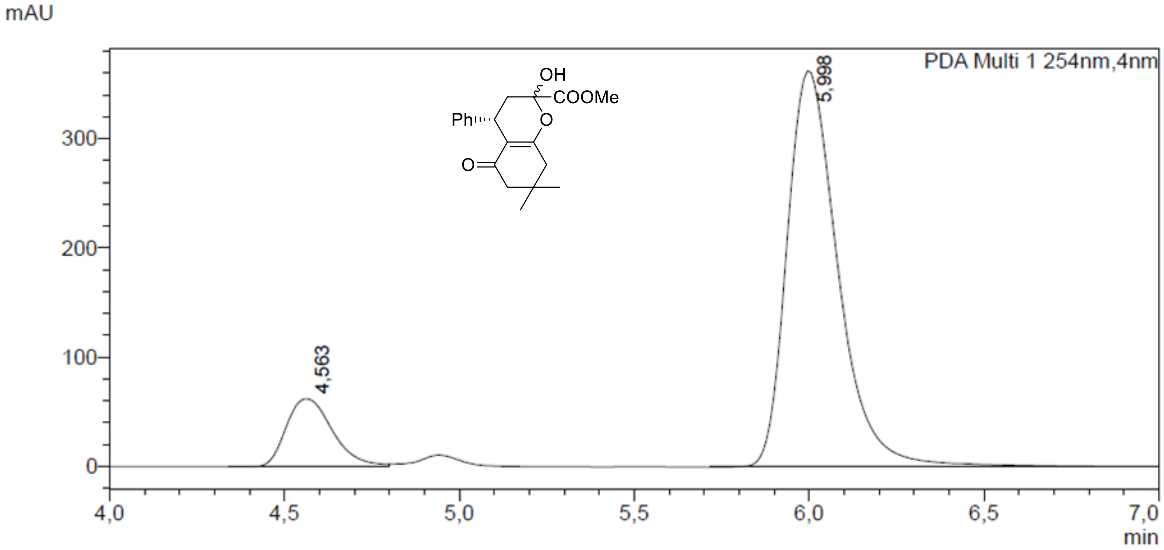

<Peak Table>

PDA Ch1 254nm

Peak#	Ret. Time	Height	Area	Area%
1	4,563	62316	575093	13,492
2	5,998	362447	3687265	86,508
Total		424763	4262358	100,000

Figure S71. HPLC chromatogram for *S*-**12**: sample obtained with catalyst *e*QD-**5j** in a planetary ball mill (73% ee).

<Chromatogram>

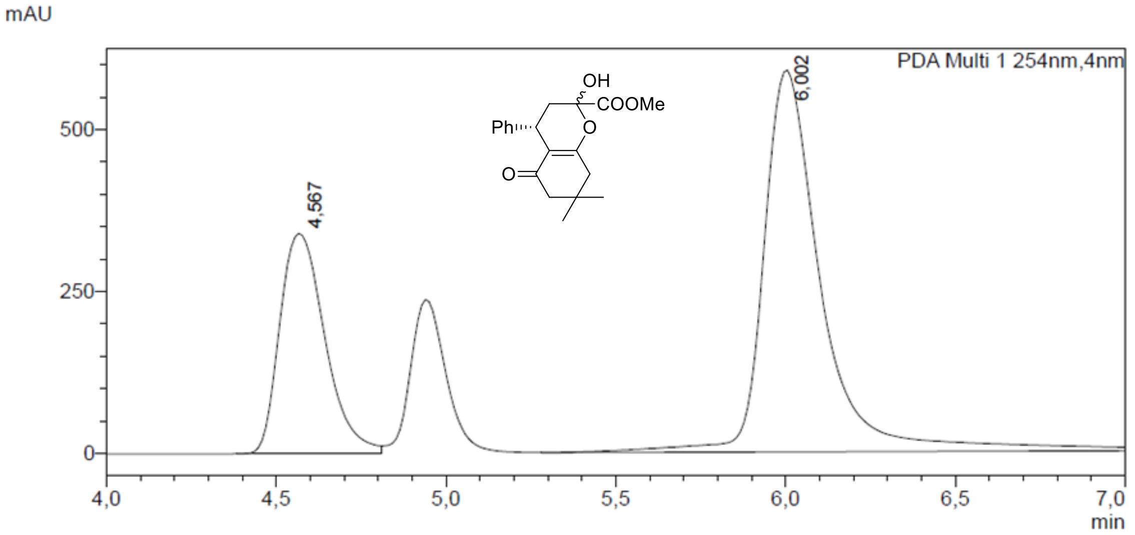

<Peak Table>

PDA Ch1 254nm

Peak#	Ret. Time	Height	Height%	Area	Area%
1	4,567	339511	36,561	3141300	30,669
2	6,002	589100	63,439	7101403	69,331
Total		928611	100,000	10242703	100,000

Figure S72. HPLC chromatogram for *S*-**12**: sample obtained with catalyst *e*DHQD-**5k** in a planetary ball mill (39% ee).

<Chromatogram>

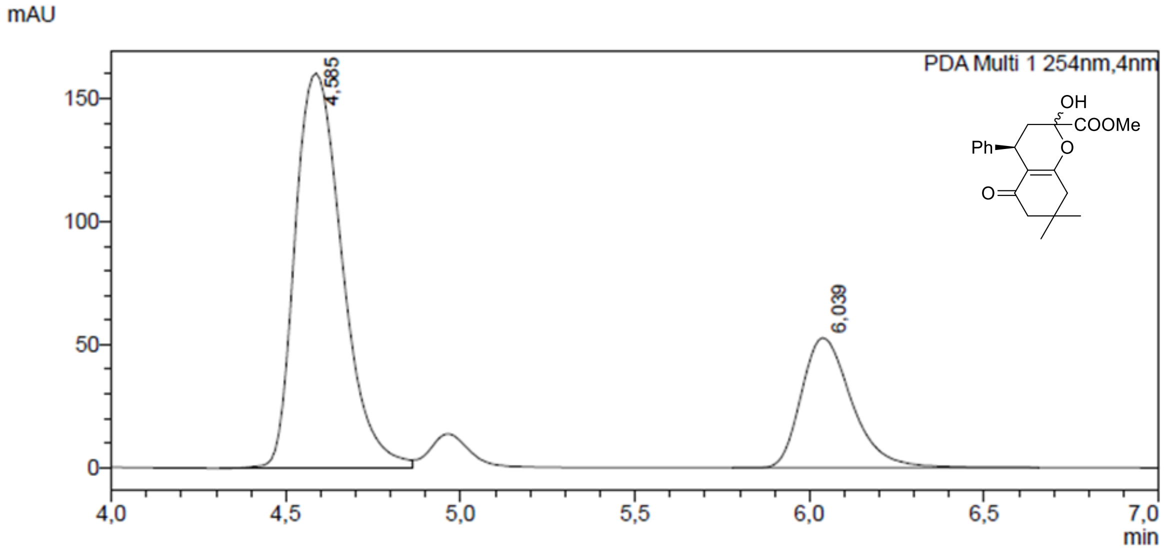

<Peak Table>

PDA Ch1 254nm

Peak#	Ret. Time	Height	Height%	Area	Area%
1	4,585	160406	75,262	1508683	74,003
2	6,039	52724	24,738	529995	25,997
Total		213130	100,000	2038678	100,000

Figure S73. HPLC chromatogram for *R*-**12**: sample obtained with catalyst *e*QN-**6a** in a planetary ball mill (48% ee).

<Chromatogram>

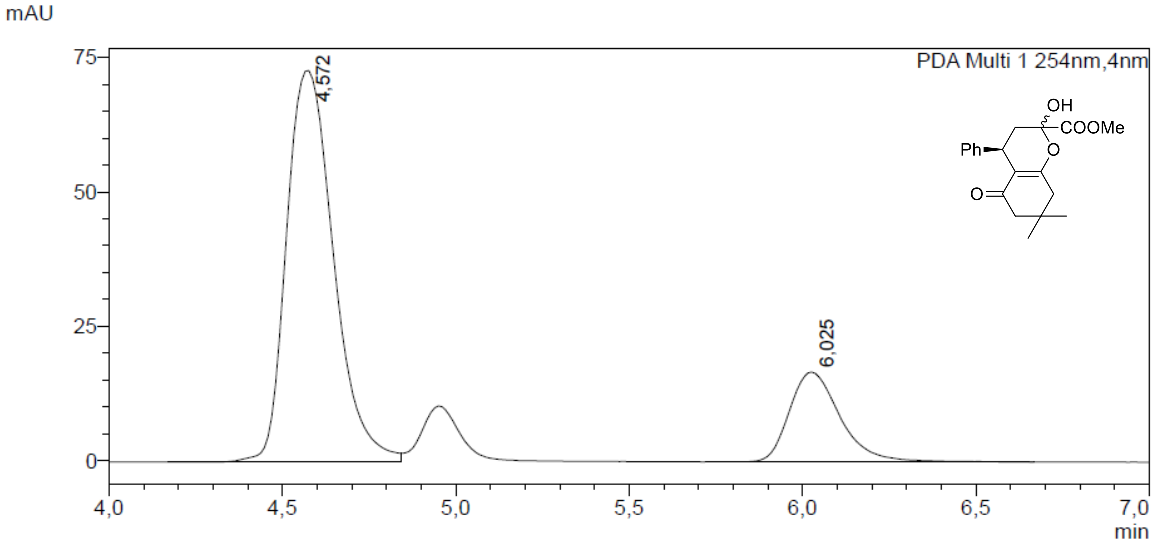

<Peak Table>

PDA Ch1 254nm

Peak#	Ret. Time	Height	Height%	Area	Area%
1	4,572	72814	81,377	689692	80,356
2	6,025	16664	18,623	168603	19,644
Total		89478	100,000	858295	100,000

Figure S74. HPLC chromatogram for *R*-**12**: sample obtained with catalyst *e*QN-**6b** in a planetary ball mill (61% ee).
